# Functional modules for enhanced amorphous composite halide solid electrolytes for low-temperature all-solid-state lithium batteries

**DOI:** 10.1038/s41467-026-71876-0

**Published:** 2026-05-27

**Authors:** Yanlong Wu, Xinmiao Wang, Xingyu Wang, Yulong Cai, Junyi Yue, Simeng Zhang, Xiangzhen Zhu, Shanshan Wang, Meng Li, Xu Han, Yi Duan, Changtai Zhao, Rong Yang, Jianwen Liang, Xiaona Li, Xueliang Sun, Jiantao Wang

**Affiliations:** 1 National Power Battery Innovation Center, China Automotive Battery Research Institute Co., Ltd, Beijing, P.R. China; 2 School of Mathematics and Science, Nanyang Institute of Technology, Nanyang, Henan P.R. China; 3 GRINM (Guangdong) Institute for Advanced Materials and Technology, Foshan, Guangdong P.R. China; 4 Eastern Institute for Advanced Study, Eastern Institute of Technology, Ningbo, Zhejiang P.R. China; 5 Beijing Advanced Innovation Center for Materials Genome Engineering, Beijing Key Laboratory for Magneto-Photoelectrical Composite and Interface Science, School of Materials Science and Engineering, University of Science and Technology Beijing, Beijing, P.R. China; 6 College of Chemistry and Environmental Engineering, Shenzhen University, Shenzhen, Guangdong P.R. China

**Keywords:** Batteries, Energy, Batteries

## Abstract

Solid-state electrolytes (SSEs) are the essential component of all-solid-state batteries (ASSBs). Designing better SSEs holds the key to the success of the ASSBs. Here, this study effectively realises the design of SSEs through functional modules. Various functional designs have been achieved by incorporating different functional models. Here, we initially introduce LaCl_3_, which possesses a UCl_3_ structure, as a functional module to demonstrate the feasibility of our approach. The Li_2_O-1.8TaCl_5_-0.2LaCl_3_ (LTLOC) SSE enable the ASSB with LiNi_0.88_Co_0.09_Mn_0.03_O_2_ (NCM88) to exhibit stable cycling and stable operation at low temperature (−30 °C). Additionally, various types of functional modules, including chloride, oxide, and fluoride, have been successfully introduced, further supporting the universality of amorphous functional module design. Furthermore, the incorporation of low-cost and low-density AlF_3_ highlights the benefits of this design approach, as it allows for a high proportion of fluoride to be introduced without compromising ionic conductivity. Li_2_O-1.8TaCl_5_-5AlF_3_ (LTOC-5AlF_3_) exhibits stability in humid conditions, resistance to high voltage, and compatibility with lithium metal simultaneously. The key strength of this design approach is its ability to maintain advantages and make up for the shortcomings.

## Abstract (web summary)

Designing improved halide solid electrolytes holds the key to the success of all-solid-state batteries. Here, authors present the design of SSEs through functional modules, introducing LaCl3, which possesses a UCl3 structure, as well as low-cost and low-density AlF3, to enable low-temperature application and improve moisture and high-voltage stability in halide-based solid-state battery systems.

## Introduction

Lithium-ion battery technology has revolutionised the energy sector^1^. The increasing demand for energy storage has raised concerns about the safety and energy density of these batteries^2,3^. All-solid-state batteries (ASSBs) are designed to address these issues by replacing flammable organic electrolytes with noncombustible inorganic solid electrolytes^4^.

Solid-state electrolytes (SSEs), the essential component of ASSBs, must meet specific criteria such as high ionic conductivities, a wide electrochemical window, good mechanical properties, and electrochemical stability^3,5^. Sulfide SSEs demonstrate high ionic conductivities, but their narrow electrochemical window complicates direct use with commercial positive electrode materials^6,7^. Conversely, oxide SSEs provide a wide electrochemical window but lack sufficient ionic conductivities and mechanical properties, hindering their development^8^. In 2018, Tetsuya Asano et al. introduced the halide SSEs (Li_3_YCl_6_ and Li_3_YBr_6_) with good overall performance, attracting significant interest due to their electrochemical properties^9^. Subsequently, researchers have developed various halide SSEs, including Li_x_ScCl_3+x_ with high ionic conductivity^10^, Li_3_InCl_6_ suitable for large-scale synthesis^11^, Li_2_ZrCl_6_ with cost-effective^12^, SmCl_3_·0.5LiCl and Li_0.388_Ta_0.238_La_0.475_Cl_3_ with the UCl_3_ framework^13,14^, and doped halide SSEs with comparable characteristics^14–17^. Doping with Ta^5+^ leads to the formation of a three-dimensional migration network within the one-dimensional Li^+^ diffusion channels of the LaCl_3_ structure, effectively enhancing ionic transport^14^. Furthermore, doping can regulate ionic conductivity by lowering the migration barrier. This can be achieved either by tuning the mixing entropy of the material system or by modulating the covalency of the bonds between metals and halides^15,16^. In addition to improving ionic conductivity, doping is essential for regulating the chemical and electrochemical stability of the electrolyte. Research has demonstrated that the O-doping strategy can partially substitute Cl in Li_3_InCl_6_, thereby simultaneously enhancing its oxidative stability and interfacial stability with sulfide electrolytes^17^.

Regarding ion transport, amorphous halide (or oxyhalide) SSEs have shown notable progress, with ionic conductivity reaching 10^−2^ S/cm, comparable to that of liquid electrolytes^18,19^. Their high ionic conductivities mainly result from their long-range disordered aperiodic structure and unique short-range ordered segments or groups^20,21^. Li et al. highlighted the critical role of the amorphous phase in ion transport for the SSEs design^22^. However, ionic conductivity is just one aspect of an electrolyte material, and an ideal electrolyte must possess a combination of properties^4^. Many modification methods prioritise one property over another, often sacrificing ionic conductivity^23^. This approach underscores the importance of optimising all properties of an electrolyte while maintaining high ionic conductivity^24,25^.

Amorphous SSEs offer several advantages, including reduced structural limitations, a broader range of component modifications, and an expanded material design space^26^. The compositional design of amorphous halide (or oxyhalide) SSEs with high ionic conductivities facilitates the optimisation of SSE performance. This study introduces a strategy for SSE design by categorising SSEs into two modules: the transport module (amorphous matrix) and the functional module. This approach allows for functional customisation through functional modules while preserving the high ionic conductivity of the amorphous component. The modular design enables diverse functionalities by incorporating various functional modules. The introduction of LaCl_3_, which possesses a large-sized UCl_3_-type structure with a *P6*_*3*_*/m* space group, aims to validate the feasibility of the functional module design concept. Incorporating LaCl_3_ enhances ionic transport at the interface between the amorphous region and the functional module, thereby sustaining high ionic conductivity. Furthermore, the Li_2_O-1.8TaCl_5_-0.2LaCl_3_ (LTLOC) SSE containing La elements forms a highly stable interfacial layer with good kinetics in situ with LiNi_0.88_Co_0.09_Mn_0.03_O_2_ (NCM88), allowing the ASSBs to demonstrate great rate capability, cycling stability, and low-temperature performance. This study demonstrates the ability to achieve stable cycling and low-temperature operation (−30 °C) in ASSBs. A variety of functional modules, including chloride, oxide, and fluoride, can be integrated into the SSEs to preserve the amorphous state while maintaining high ionic conductivities. This finding further substantiates the universality of the design of amorphous functional modules. The inclusion of low-cost and low-density AlF_3_ underscores the advantages of this design approach, as it permits a substantial proportion of fluoride to be introduced without compromising ionic conductivity. The composition Li_2_O-1.8TaCl_5_-5AlF_3_ (LTOC-5AlF_3_) demonstrates high ionic conductivity, stability under humid conditions, resistance to high voltage, and compatibility with lithium metal. This research utilises various functional modules in material design to effectively regulate multiple functions, including ionic conductivity, electrochemical stability, interface design, humidity stability, high-pressure stability and lithium metal stability, thereby showcasing the progress and versatility of functional module design.

## Results

### Design and synthesis of solid-state electrolytes

**Fig. 1:**
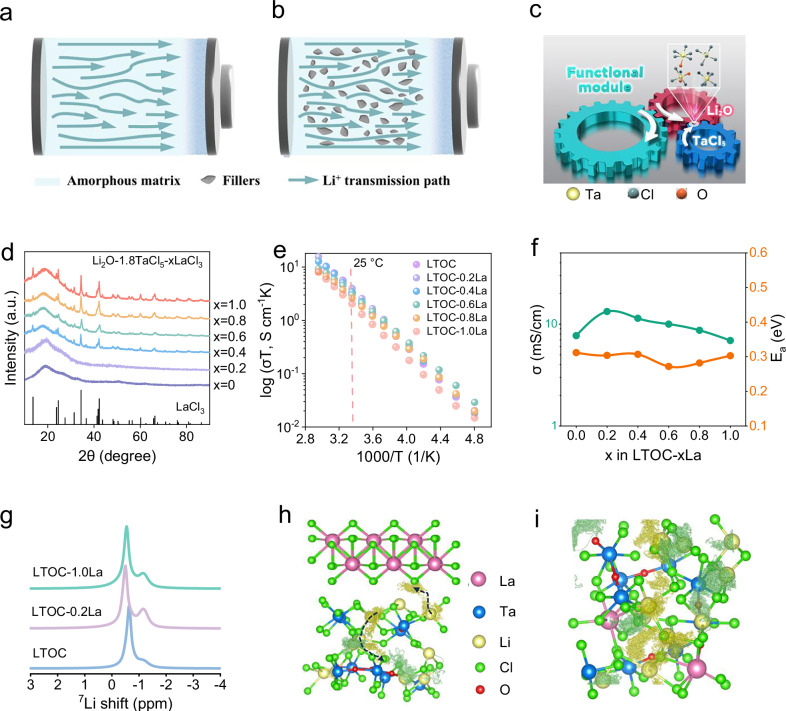
Design and synthesis of solid-state electrolytes. **a**,** b** Schematic diagram of Li^+^ conduction in amorphous solid-state electrolytes. **c** Schematic diagram of function module design. **d** XRD patterns of LTOC-xLa with different compositions. **e** Arrhenius plots of LTOC-xLa. **f** Variation of the ionic conductivities (σ) at 25 °C and the activation energy (E_a_) with x of LTOC-xLa. **g** ^7^Li 1D NMR spectra of LTOC, LTOC-0.2La, and LTOC-1.0La. **h**, **i** The structure and potential isosurface of LTOC-0.2La are visualised using VESTA.

Unlike crystalline materials with rigid structures, amorphous oxyhalide SSEs benefit from long-range disorderliness^27^. This eliminates dependence on a fixed structure for ion transmission, leading to a more flexible and efficient path for ion movement (Fig. 1a). Many amorphous SSEs exhibit comparable ionic conductivities^18,27,28^. The greater flexibility of the transmission path of lithium ions in amorphous allows it to overcome the limitations of the crystal structure framework^21,27^. The ion transport path can be likened to river tributaries, where the obstruction of a branch has an insignificant effect on the flow rate. Inspired by this concept, incorporating functional modules into amorphous electrolytes may create obstacles, but does not significantly hinder ion conductivity as long as the amorphous transport network remains intact (Fig. 1b). Therefore, we proposed regulating the properties of amorphous SSEs through a functional module design. The designed SSEs comprise two components: the ion transport part (amorphous LTOC) and the functional module part (Fig. 1c). This unique structure ensures efficient ion transmission and allows for artificial control using functional modules based on specific requirements. Importantly, the function module emphasises that the addition of the module has a minimal impact on the conduction mechanism of the amorphous material; however, it can serve a great role in functional design. The functional modules can promote the formation of either a homogeneous amorphous phase or a composite structure comprising an amorphous matrix and a secondary phase, a characteristic commonly observed in polymer electrolytes^29^. While functional modules may somewhat affect ion transport, the focus will be on the module’s functional design and key role rather than delving too deeply into its impact on ion transport. This approach will not compromise the overall advanced design concept, as an excellent electrolyte material must possess a well-rounded set of properties that warrant thorough evaluation.

The SSEs were designed with LTOC serving as the amorphous ion-conductive framework and LaCl_3_ as a functional module. Different compositions of LaCl_3_ were tested (0.2LaCl_3_, 0.4LaCl_3_, 0.6LaCl_3_, 0.8LaCl_3_ and 1.0LaCl_3_) and labelled as LTOC-0.2La, LTOC-0.4La, LTOC-0.6La, LTOC-0.8La and LTOC-1.0La. The X-ray diffraction (XRD) analysis of the ball-milling synthesised LTOC revealed no distinct characteristic peaks, confirming its amorphous characteristic (Fig. 1d). The ionic conductivity of LTOC was measured at 7.7 mS/cm (Fig. S1). Introducing a small amount of LaCl_3_ in LTOC markedly improved ionic conductivity to 13.3 mS/cm, indicating compatibility between the LTOC structure and LaCl_3_. Further increase in LaCl_3_ content led to partial LaCl_3_ crystallisation as observed in XRD, resulting in decreased lithium-ion conductivity, possibly due to ion transport path hindrance (Fig. 1e, f, Fig. S2 and Table S1). Additionally, lattice fringes corresponding to LaCl_3_ were also observed using high-resolution transmission electron microscopy (HRTEM) (Fig. S3). The combination of XRD and HRTEM provides strong evidence for the existence of LaCl_3_.

To elucidate the enhanced ionic conductivity of LTOC-0.2La (LTLOC) compared to LTOC, solid-state nuclear magnetic resonance (SSNMR) was utilised to analyse the Li^+^ chemical environments in SSEs^30^. In the 1D ^7^Li NMR spectra of LTOC, two chemical shift peaks at −0.59 and −1.22 ppm (referred to as Li1 and Li2, respectively) were observed, indicating Li1 in the amorphous phase and Li2 in a Li structure/chemical coordination environment similar to LiCl sites within the LTOC lattice (Fig. 1g)^20,27^. The chemical shift of Li towards the left in LTOC-0.2La and LTOC-1.0La suggested a modified Li environment in the amorphous phase due to LaCl_3_ introduction^14,27^. The presence of a higher number of Cl^-^ ions surrounding La sites led to lower electron density, resulting in a weaker shielding effect and downfield shifting of Li^+^ resonance. Additionally, the relative intensity of the peak at −1.22 ppm increased. Analysis using ^7^Li-^7^Li 2D exchange NMR spectroscopy (2D-EXSY) demonstrated the interexchange strength of Li^+^ between Li1 and Li2. The two self-correlation peaks along the diagonal in 2D-EXSY represented Li^+^ chemical exchange or spin diffusion (Fig. S4)^14,31^. Through both experimental and theoretical calculations, we have gained a deeper understanding of the mechanism by which the introduction of LaCl_3_ enhances the ionic conductivity of LTOC-xLa. We identified two primary reasons for this enhancement. LaCl_3_ interacts with the amorphous phase of LTOC, facilitating lithium-ion transport at the interface, and the doping effect of LaCl_3_, particularly the introduction of La^3+^, plays a crucial role in enhancing ionic conductivity (Fig. 1h, i and Figs. S5 and S6). Detailed analysis content can be found in the supporting information.

However, overall ionic conductivity remained high due to the unique migration mode of the LTOC’s amorphous structure. Decreasing TaCl_5_ content while keeping LaCl_3_ constant markedly reduced ionic conductivity, highlighting the crucial role of LTOC’s amorphous component in ion transport (Fig. S7). Detailed information is available in the Supplementary Information (Table S1). The electronic conductivities of LTOC and LTLOC were 6.1 × 10^−9^ and 3.0 × 10^−10^ S/cm, respectively, evaluated by the direct current polarisation (DC) method (Fig. S8). The LTLOC synthesised using high-energy ball milling exhibited relatively small particles. Scanning electron microscopy (SEM) observation revealed a particle size distribution of 1–10 μm, with a predominant size of 2–3 μm and an irregular polyhedral shape (Fig. S9). The energy dispersive spectroscopy (EDS) analysis shows a uniform distribution of elements such as Ta, La, O and Cl, suggesting homogeneity in the material (Fig. S10). It was further confirmed by the cryo-transmission electron microscopy (Cryo-TEM) that LTLOC was amorphous, and the elements were evenly distributed (Fig. S11). We explored the influence of the amount of functionalized modules on the physical properties of solid-state electrolyte materials, such as morphology, particle size, and Young’s modulus (Fig. S12-14).

### Electrochemical characterisations of the SSEs at 25 °C

**Fig. 2:**
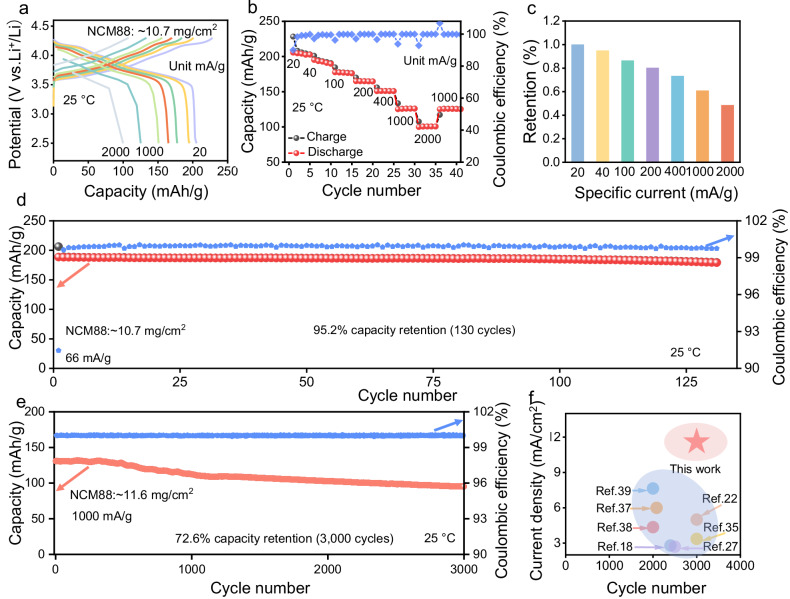
Electrochemical performance of Li-In|LPSC|LTLOC|NCM88 (LTLOC|NCM88) at 25 °C. **a** Discharge/charge curves and **b** rate performance of the LTLOC|NCM88 ASSB at different specific currents (20, 40, 100, 200, 400, 1000, and 2000 mA/g). **c** The average discharge capacity retention rate. **d** Long-term cycling performance and coulombic efficiency at 66 mA/g. **e** Long-term cycling performance and coulombic efficiency at 1000 mA/g (3000 cycles). **f** Comparison of cycling number and current density of ASSBs with different solid electrolytes (1C = 200 mA/g and the cell loading >4 mg/cm^2^).

The electrochemical stability of the SSEs was evaluated using linear sweep voltammetry (LSV) with an asymmetric ASSB^32^. The thermodynamic electrochemical stability window of LTLOC was 2.42–4.11 V (vs. Li^+^/Li) (Fig. S15). Commercial uncoated single-crystal LiNi_0.88_Co_0.09_Mn_0.03_O_2_ (NCM88) was used as the positive electrode active material to evaluate the compatibility between the oxide positive electrode and LTLOC (or LTOC). The ion transport performance of the SSE was also reflected in ASSBs, especially in high rate and long cycles. The Li-In|Li_6_PS_5_Cl (LPSC)|LTLOC|NCM88 (LTLOC|NCM88) ASSB exhibited good rate performance, as shown in Fig. 2a, b, better than the Li-In|LPSC|LTOC|NCM88 (LTOC|NCM88) ASSB (Fig. S16a, b). The charge-discharge curves depict high reversible capacities of 205.6, 195.2, 177.6, 165, 150.8, 125.2 and 99.9 mAh/g at 20, 40, 100, 200, 400, 1000 and 2000 mA/g, respectively. The average discharge capacity retention rate at different rates is shown in Fig. 2c (based on the discharge specific capacity of the specific current 20 mA/g). The high ionic conductivity of LTLOC SSE is a key factor contributing to its good rate performance. In particular, the ASSB exhibited cycling durability even at 1000 mA/g, as demonstrated in the rate capability evaluation (Fig. 2e). The rate capability of the LTLOC | NCM88 ASSB stemmed from its high ionic conductivity and great interfacial diffusion kinetics. The cycle stability of the LTOC|NCM88 ASSB was poor, as evidenced by a capacity retention rate of only 66.4% after 200 cycles, which was significantly lower than the 94.7% observed for the LTLOC|NCM88 ASSB (Fig. S16c). To assess the relationship between LTOC/LTLOC and LPSC, separate Li-In|Li_2.5_Y_0.5_Hf_0.5_Cl_6_ (LYHC)|LTOC (or LTLOC)|NCM88 ASSBs were assembled for comparison^33^. The results showed that the cycle stability of Li-In|LPSC|LTOC|NCM88 ASSBs was significantly lower than that of Li-In|LYHC|LTOC|NCM88 ASSBs, while there was no obvious difference between Li-In|LPSC|LTLOC|NCM88 and Li-In|LYHC|LTOC|NCM88 (Fig. S16d). This difference may be attributed to the instability of LTOC and LPSC, which consequently hinders the widespread use of LTOC at high rates^34^. Given the stability of LTLOC and LPSC, further analysis of the long-cycle performance of LTLOC ASSB is warranted. The cycling number and current density of the LTLOC | NCM88 ASSBs are comparable to those of previously reported halogen-based ASSBs (Fig. 2f)^18,22,27,35–39^. This further improves the fast charging cycle life limit and fast discharge of halogen-based ASSBs at large rates.

Although the ASSBs mentioned above exhibited comparable electrochemical performance (compared with previously reported results) at a high rate and long cycling, good performance under other conditions is also desirable^40,41^. Energy density can be effectively improved by increasing the proportion of positive electrode active material and the loading. For comparison, we fabricated an NCM88 ASSB with a positive electrode composite containing 85 wt% NCM88 and 15 wt% SSE. The ASSB exhibited a high discharge capacity (202 mAh/g) and good cycling behaviour (94.7% capacity retention over 200 cycles) (Fig. S17a, d). Figure. S17b, e show the results for high-loading ASSBs (19.5 mg/cm^2^, NCM88; 3.9 mAh/cm^2^) cycled at 25 ± 0.1 °C. They delivered stable capacity retention and high specific capacity (>3 mAh/cm^2^ and >170 mAh/g over 100 cycles). Additionally, an NCM88 ASSB was fabricated with a positive electrode composite comprising 85 wt% NCM88 and 15 wt% SSE, alongside high-loading ASSBs. The ASSB exhibits slightly lower discharge capacity (164 mAh/g) and cycling behaviour (87.8% capacity retention over 60 cycles) (Fig. S17c, f) compared to the ASSB with only 85 wt% NCM88 or high-loading ASSBs. We attribute this to poor ionic conduction percolation within the positive electrode composite due to the lower SSE and higher positive electrode content, which may be improved by further SSE nanostructuring.

### Electrochemical characterisations of the SSE at low temperature (−30 °C)

**Fig. 3:**
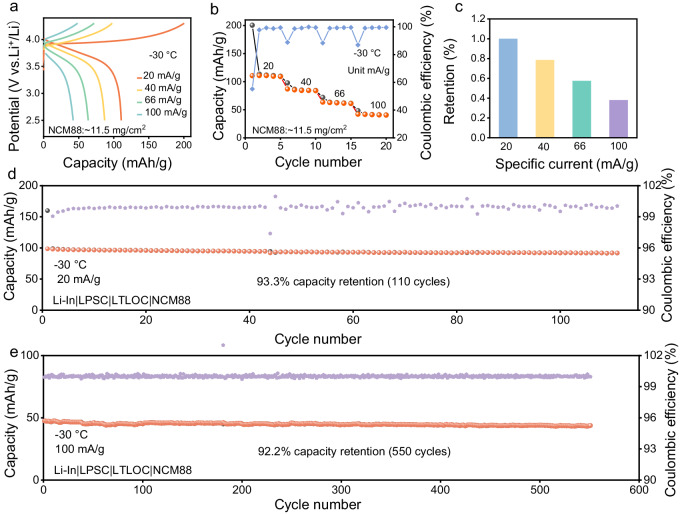
Electrochemical performance of Li-In|LPSC|LTLOC|NCM88 (LTLOC|NCM88) at −30 °C. **a** Discharge/charge curves and **b** rate performance of the LTLOC|NCM88 ASSB at specific currents (20, 40, 66 and 100 mA/g). **c** The average discharge capacity retention rate. **d** Long-term cycling performance and coulombic efficiency at 20 mA/g. **e** Long-term cycling performance and coulombic efficiency at 100 mA/g (550 cycles) (1C = 200 mA/g).

Low-temperature electrochemical performance is critical for ASSB applications. The LTLOC|NCM88 ASSB exhibits comparable electrochemical performance at low temperatures, delivering a high specific capacity of 110 mAh/g at −30 °C with an initial coulombic efficiency of 55.2% (Fig. 3a). The low coulomb efficiency may be related to ion diffusion difficulty at low temperatures. Figure 3b shows the rate performance at various rates. The average discharge capacity retention rate at different rates is shown in Fig. 3c (based on the discharge specific capacity of 0.1 C). In addition, the LTLOC|NCM88 ASSB exhibited long-term cycle stability at low temperatures. As depicted in Fig. 3d–f, a capacity retention of 93.3% was observed after 110 cycles at 20 mA/g (−30 °C) and a capacity retention of 92.2% was observed after 550 cycles at 100 mA/g (−30 °C (Fig. 3e). These results underscore its low-temperature electrochemical performance, which is attributed to the LTLOC SSE’s high ionic conductivity and favourable interface contact and transport properties at low temperatures. The subsequent section extensively discusses interfacial diffusion kinetics.

### Origin of good electrochemical performance

The positive electrode’s morphology and element distribution were examined after cycling using SEM and EDS. SEM analysis revealed that NCM88 and LTLOC remained in close contact without any visible cracks in the composite positive electrode after 200, 1000 and 50,000 cycles, respectively (Fig. S19a–c). This indicates satisfactory mechanical compatibility between NCM88 and LTLOC during extended cycling, contributing to the enhanced stability of the LTLOC|NCM88 ASSB. Furthermore, EDS analysis revealed an even distribution of Ta and La elements (Fig. S19d–i). The diffusion coefficient during charge and discharge was evaluated using GITT, as shown in Fig. S20, which presents the charge and discharge GITT curves. Assemble batteries using the same positive electrode material while varying the SSEs to investigate the effects of different SSEs on the composite positive electrode material interface and the overall battery performance. Compared to LTOC|NCM88 ASSB, LTLOC|NCM88 ASSB has higher ion diffusion. A higher lithium-ion diffusion coefficient suggests a lower transmission barrier at the interface between the LTLOC and the positive electrode.

Complex electrochemical processes within ASSB systems markedly contribute to ASSB cycling but are difficult to identify. Hence, clarifying Li kinetics poses a great challenge, especially for next-generation battery systems. The comparable electrochemical performance of the LTLOC|NCM88 ASSB highlights the critical role of intricate electrochemical processes within the ASSB system throughout cycling. Electrochemical impedance spectroscopy (EIS) is a valuable tool to reveal the electrochemical process on a time scale, offering high accuracy and non-destructive capabilities^42^. Understanding the “black box” system through EIS is essential for effective battery diagnosis. The distribution of relaxation time (DRT) directly identifies the time constants of key electrochemical processes, streamlining impedance analysis and enhancing kinetic interpretation accuracy over various time scales^43^. This study employed EIS and DRT to intensively uncover LTLOC|NCM88 ASSB dynamic processes.

**Fig. 4:**
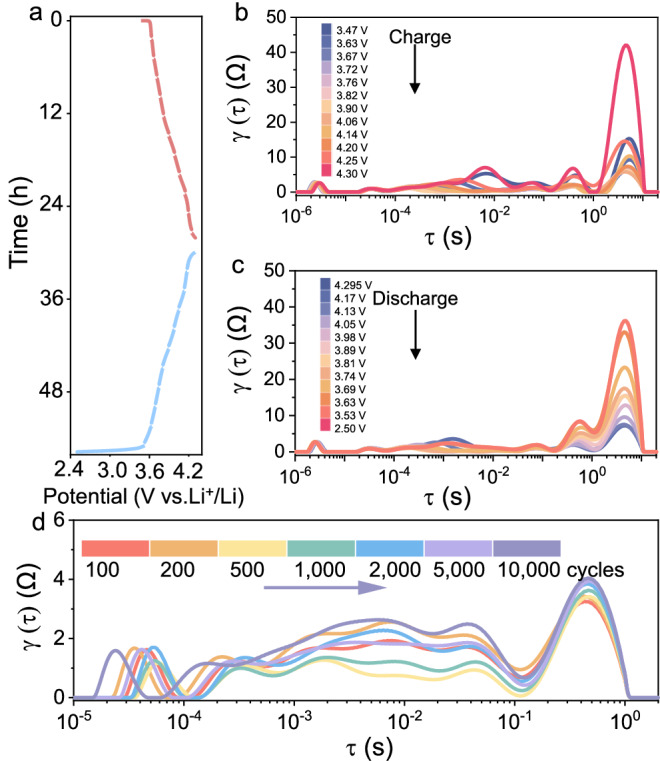
Dynamic analysis of LTLOC|NCM88 by electrochemical impedance spectroscopy (EIS) and distribution of relaxation time (DRT). **a** The in situ charge-discharge curve and the discontinuous point align with the EIS testing point. **b** DRT is calculated from the EIS measurements in charge. **c** DRT is calculated from EIS measurements during discharge. **d** DRT calculated from EIS measurements at different cycles.

Figure 4a illustrates the in situ charge-discharge curve, with red representing the charge curve and green representing the discharge curve. The discontinuous point aligns with the EIS testing point. Detailed information is available in the methods section. Broadly categorised from low to high frequencies, the curve can be segmented into conduction-based processes, charge transfer-based processes, physical contact, and diffusion processes contact impedance. These correspond to the impedance of SSE, Li^+^ impedance through solid electrolyte interphase (SEI), Li^+^ impedance through chemical-electrochemical interface (CEI), and charge transfer impedance in positive electrode material^43,44^. In situ EIS shows the impedance change during charging and discharging (Fig. S21). Li^+^ migrates from the positive electrode during the charging process with relatively stable impedance in the low-frequency region, indicating minimal electrolyte decomposition and stability. Changes in the middle-frequency region suggest the gradual formation of the SEI and CEI layers. The high-frequency region initially decreases and then increases, reflecting variations in the Li^+^ diffusion coefficient of the positive electrode material with lithium content (Fig. 4b and Fig. S22a). The discharge process mirrors the charging process but exhibits notable differences in the high-frequency region. As discharge progresses, impedance gradually rises, slowing the charge transfer within the positive electrode (Fig. 4c and Fig. S22b).

To further evaluate the dynamic behaviour behind the electrochemical properties, the change in ASSBs impedance under varying cycle numbers was analysed by DRT. Following numerous cycles, impedances at different frequencies were consistently low (Fig. 4d and Fig. S22c). Despite fluctuations in impedance values at mid and low frequencies, they remained small throughout, suggesting good diffusion kinetics at the interface of the positive electrode material and SSE during cycling. Overall impedance showed minimal changes with increasing cycle numbers, indicating a highly stable interface between the positive electrode and SSE over extended cycles. This stable interface layer, facilitating Li^+^ migration, markedly contributes to the long-cycle life of LTLOC|NCM88 ASSB.

**Fig. 5:**
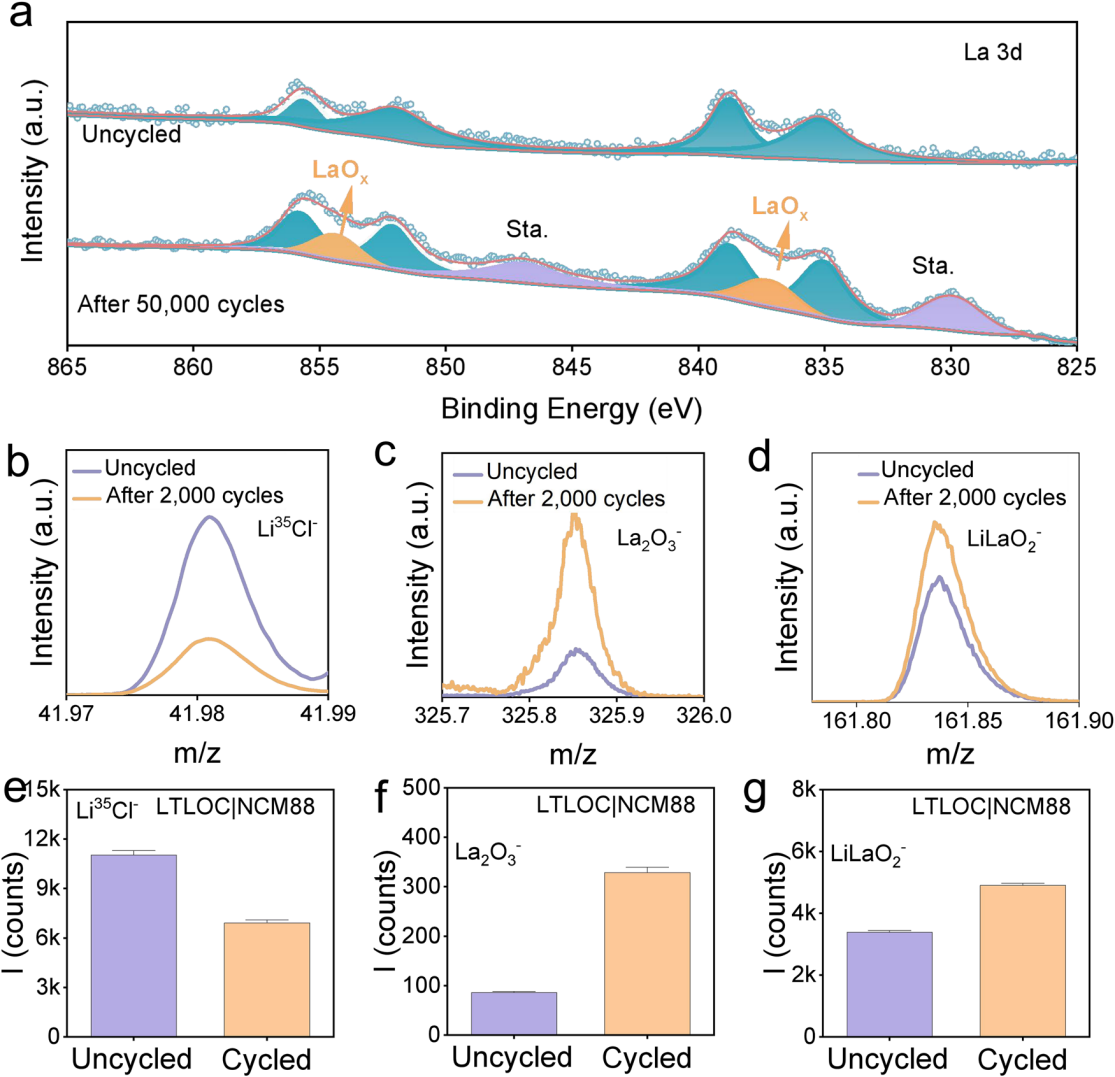
Interface evolution between NCM88 and LTLOC after long cycling. **a** XPS spectra of La 3 d at uncycled and after 50,000 cycles (based on the discharge specific capacity of the specific current 2000 mA/g). **b**–**g** ToF-SIMS surface analysis results of **b**, **e** Li^35^Cl^-^, **c**, **f** La_2_O_3_^−^, **d**, **g** LiLaO_2_^−^ at uncycled and after 2000 cycles (based on the discharge specific capacity of the specific current 200 mA/g). The bar chart were obtained based on each sample’s corresponding normalised signal intensities of nine spectra. Error bars are standard errors of the mean, and the bar is the average.

After validating the significance of the interface layer in ASSBs using EIS and DRT, its composition was further elucidated^45^. X-ray photoelectron spectroscopy (XPS) analysis revealed new peaks at binding energies of 853.6 and 856.3 eV after 200 and 50,000 cycles, suggesting a change in the La environment. These peaks resulted from LaO_x_ formation, indicating the development of a clear oxide layer on La after extended cycling (Fig. 5a and Figs. S23–S25)^46,47^. Moreover, the time-of-flight secondary ion mass spectrometry (ToF-SIMS) analysis of the interface products revealed that the concentration of ClO^−^ remained relatively stable after cycling, suggesting minimal chemical degradation (Fig. S28). Conversely, LiCl^-^ decreased while La_2_O_3_ and LiLaO_2_ increased (Fig. 5b–g)^35,48,49^. Consequently, the highly stable interface layer primarily comprises stable oxides like La_2_O_3_ and LiLaO_2_ generated in situ, exhibiting better uniformity and compactness (Figs. S26–S28). Additionally, LiLaO_2_ acts as a lithium-ion conductor, facilitating rapid lithium-ion diffusion at the interface.

### The universality of functional module design

Additional materials have been developed to demonstrate the universality of functional module design. By incorporating small quantities of functional modules, ionic conductivity is enhanced while maintaining the amorphous structure. This approach incorporates various functional module types, such as chlorides, fluorides, and oxyhalides. Notably, all these functional modules effectively contribute to developing materials with high ionic conductivity (Figs. S30–32, 6a and Table S5)^9–12,14,18,27,37,38,50–53^. The findings suggest that the design of high ionic conductivity SSEs extends beyond functional module types, thus expanding the design possibilities and confirming the viability of functional module design. We utilised SSNMR to analyse the changes in the local environment of lithium following the introduction of various types of functional modules. When ZrCl_4_ is introduced, only one peak is observed in the NMR spectrum, which corresponds to lithium in an amorphous environment. Lithium in isolation or a less complex environment exhibits relatively weak signals. The introduction of additional substances causes the lithium peak, indicative of the amorphous environment in LTOC, to shift to a higher position (~−0.62 ppm), while the position of the other peak remains nearly unchanged. The degree of peak shift varies with the introduction of different substances, likely due to the differing interactions between these substances and LTOC. Fluoride exhibits the smallest deviation, followed by oxide and chloride. In summary, the NMR data indicate that the introduction of various substances has minimal impact on the local environment of lithium within the LTOC amorphous module, suggesting that the addition of the functional modules only induces small changes in the material susceptibility (the small shifts) and does not significantly disrupt the amorphous conduction network of the LTOC and the basic ion transport characteristics of the amorphous functional module are preserved (Fig. S33).

**Fig. 6:**
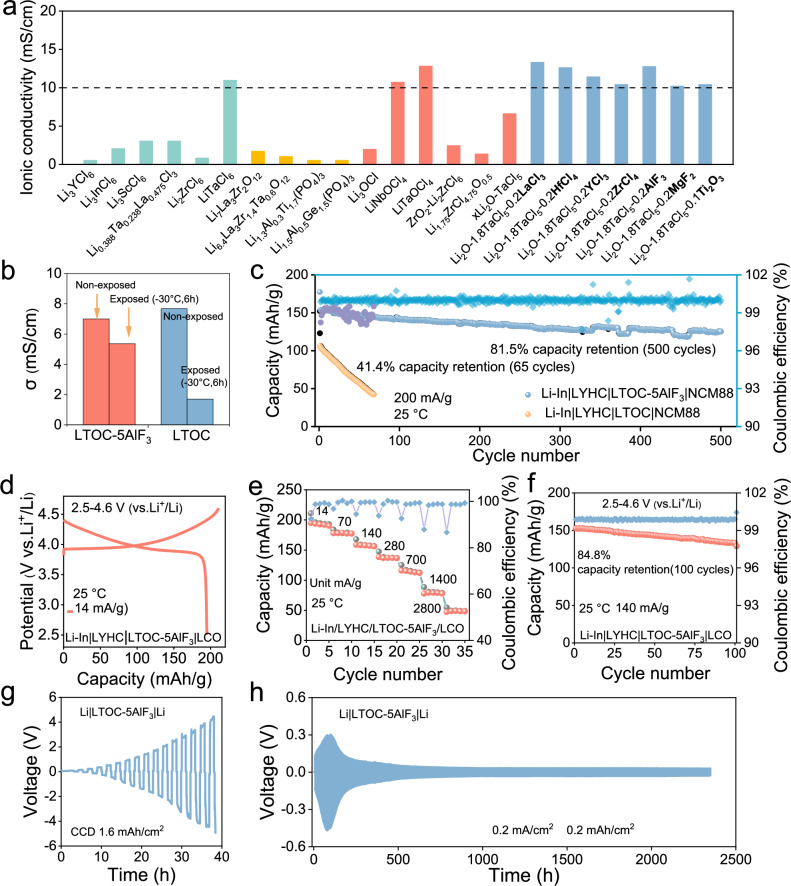
The universality of functional module design. **a** Comparison of functionalized SSEs with Others (Details are in Supplementary information Table S5)^9–12,14,18,27,37,38,50–53^. **b** Comparison of ionic conductivities between LTOC-5AlF_3_ and LTOC after exposure to air 6 h. **c** Long-term cycling performance and coulombic efficiency of LTOC-5AlF_3_ and LTOC in a drying room (dew point of −30 °C) after exposure 6 h (Li-In|LYHC|LTOC-5AlF_3_|NCM88 and Li-In|LYHC|LTOC|NCM88)(1 C = 200 mA/g). **d–f** Electrochemical performance of Li-In|LYHC|LTOC-5AlF_3_|LCO at 4.6 V (1 C = 140 mA/g). **g** The critical current densities (CCD) measurements of Li|LTOC-5AlF_3_|Li. **h** Voltage profile of the Li|LTOC-5AlF_3_|Li symmetric cell Li plating/stripping under a current density of 0.2 mA/cm^2^ and an areal capacity of 0.2 mAh/cm^2^.

The functional module approach offers greater flexibility than traditional doping methods, exhibiting less sensitivity to the phase composition and the amount of functional module dopant used. Introducing a significant amount of low-cost and low-density fluoride can enhance humidity, high voltage and lithium metal stabilities due to its wide electrochemical window and humidity stability^54^. AlF_3_ incorporation initially resulted in a significant enhancement of ionic conductivity, but further addition led to a slight decline. When the ratio of LTOC to AlF_3_ is 1:5, the ionic conductivity of LTOC-5AlF_3_ remained high at 7 mS/cm, close to that of LTOC (7.7 mS/cm) (Fig. S34). The presence of an obvious AlF_3_ phase in the amorphous phase indicated limited amorphous compatibility and the existence of some AlF_3_ as heterogeneous functional modules. In the LTOC-5AlF_3_ SSE, the Cl: O: F ratio was 9:1:15, with F comprising 60% of the total anions. Despite the high F content, maintaining an ionic conductivity of 7 mS/cm was considerable in previous studies. Typically, introducing even a small amount of F would significantly reduce ionic conductivity, making it challenging to introduce high F content due to the strong F-Li bond^55^. In the 1D ^7^Li NMR spectra of LTOC and LTOC-5AlF_3_, two chemical shift peaks were observed, and the chemical shift of Li towards the left in LTOC-5AlF_3_, indicating LTOC-5AlF_3_ and LTOC have similar lithium environments, so AlF_3_ does not completely change ion transport (Fig. S38). The relatively high proportion of AlF_3_ introduced, coupled with its limited solubility in the amorphous phase, renders the presence of crystalline components inevitable. By comparing the characteristic peaks from XRD with standard PDF cards and correlating them with the lattice fringes observed in TEM, it can be inferred that the crystalline phase is AlF_3_. AIMD simulations reveal that the interaction mechanism between the functional modules and the amorphous component in LTOC-AlF_3_ is analogous, primarily driven by interface enhancement mechanisms and doping effects. Detailed analysis content can be found in the supporting information (Figs. S35–S40 and Supplementary Data 1).

In addition, the electrolyte LTOC-5AlF_3_ with a high fluorine content showed humidity stability. Exposing LTOC-5AlF_3_ and LTOC to a −30 °C dew point environment for 6 h in a drying room resulted in greatly higher retention of ionic conductivity (76.6% of pre-exposure value) for LTOC-5AlF_3_ (5.36 mS/cm) compared to LTOC (22.3% of pre-exposure value) (Fig. 6b and Fig. S41). We compared the ion conductivity of LTOC-5AlF_3_ after a two-hour exposure to a higher dew point of −10 °C. The results indicate that the ion conductivity retention rate of LTOC-5AlF_3_ is better than that of LTOC (Fig. S42). These results demonstrate that AlF_3_ enhances LTOC’s humidity stability, which is not solely reflected in ionic conductivity. Furthermore, after grinding SSE (LTOC-5AlF_3_/LTOC) and NCM88 in a drying room (dew point of −30 °C) and standing for 30 min, the ASSBs of LTOC-5AlF_3_ |NCM88 and LTOC | NCM88 were assembled separately. The charge-discharge curve of LTOC-5AlF_3_|NCM88 ASSB was normal and could be stably cycled, but LTOC|NCM88 deteriorated rapidly in the second cycle with a lower capacity (Fig. 6c). This experiment fully proves that introducing AlF_3_ as a functional module contributes to enhancing humidity stability. In addition, LTOC-5AlF_3_ showed high-voltage resistance and stability to lithium metal (Fig. 6d–h and Fig. S44). Introducing fluoride through functional module design enhances humidity stability, high-voltage resistance, and stability to lithium while maintaining ionic conductivity. This design scheme for electrolyte control is highly effective and can impart multiple beneficial properties.

## Discussion

This study presents a functional design engineered in amorphous SSEs by incorporating functional modules to enhance ionic conductivity and implementing a controlled design through functional modules. Introducing LaCl_3_ as a functional module into amorphous LTOC yielded a different SSE, LTLOC. This material enhances ionic conductivity (doubled), and a stable interface layer can be formed in situ at the positive electrode and SSEs interface. The SSEs enabled the ASSB with NCM88 to exhibit stable cycling and stable operation at low temperature (−30 °C). Interface analysis using in situ EIS, DRT, XPS and ToF-SIMS reveals the evolution and stability mechanism of the interface during prolonged cycling. The in situ generation of the oxide phase containing La_2_O_3_ and fast ionic conductor LiLaO_2_ at the interface ensures high stability and good diffusion kinetics. This process contributes to the enhanced performance of ASSBs.

Various types of functional modules, including chloride, oxide, and fluoride, can be incorporated into the SSEs to maintain the amorphous state while exhibiting high ionic conductivities. This observation further supports the universality of amorphous functional module design, potentially. Incorporating a high proportion of low-cost and low-density AlF_3_ as a functional module afforded the SSE LTOC-5AlF_3_. It highlights the benefits of this design approach, as it allows for a high proportion of fluoride to be introduced without compromising ionic conductivity. Ultimately exhibiting high ionic conductivity, stability in humid conditions, resistance to high voltage, and compatibility with lithium metal.

This study demonstrates that various functional modules can achieve multiple functions, including ionic conductivity, electrochemical stability, interface design, humidity stability, high-voltage stability, and lithium metal compatibility, showcasing the versatility and progress in functional module design, potentially. This work introduces a design concept in the realm of SSEs design. It leverages the unique advancements of amorphous SSEs to facilitate targeted functional design through functional modules, enabling directional control. The primary advantages of this design approach lie in its ability to retain benefits while compensating for limitations, collectively enhancing overall performance. Additionally, this method is not constrained by the proportions and types of materials.

## Methods

### Preparation of materials

To prepare Li_2_O-xTaCl_5_-aX_b_Y_c_, a stoichiometric mixture of Li_2_O (99.9%, Macklin), TaCl_5_(98%, Macklin)/LaCl_3_(99.9%, Macklin)/HfCl_4_(99.9%, Macklin)/YCl_3_(99.99%, Macklin)/ZrCl_4_(98%, Macklin)/AlF_3_(99.9%, Macklin)/MgF_2_(99.9%, Macklin)/Ti_2_O_3_(99.9%, Macklin) was ball-milled at 600 rpm for 6 h in a ZrO₂ vial with ZrO₂ balls via a Pulverisette 7PL (Fritsch GmbH) under Ar atmosphere. Besides, each cycle included 10 min of rotation and 5 min of rest, and the milling time stated in this work represents the net rotation time. The ball-milling time mentioned in this work is the rotation time.

The Li_6_PS_5_Cl (from China Automotive Battery Research Institute Co. Ltd) had a particle size of 2–4 µm.

### Physical characterisation

The X-ray diffraction (XRD) patterns were collected at a scanning rate of 10°/min over the 2θ range of 10–90° using a PANalytical Empyrean diffractometer with Cu Kα radiation (λ = 1.54056 Å). All samples were sealed with Kapton film to prevent exposure to air and moisture. The LTOC-xLa and LTOC-5AlF_3_ samples were prepared via ball milling, then uniformly mixed with LaB_6_ or Si powder at a mass ratio of 9:1. The proportion of the amorphous component in the LTOC-xLa and LTOC-5AlF_3_ samples was quantified by Rietveld refinement against the XRD patterns, with LaB₆ or Si employed as the internal standard. Scanning electron microscopy (SEM) images were acquired using a JSM-7900F field emission SEM coupled with energy dispersive spectroscopy (EDS) (the beam voltage is 15 kV). The X-ray photoelectron spectroscopy (XPS) measurements were performed on a Kratos AXIS Ultra spectrometer using monochromated Al Kα radiation (15 kV). All binding energies were calibrated using the C 1 s peak of adventitious carbon at 284.8 eV, and samples were transferred via a dedicated inert-atmosphere transfer holder to avoid contamination. A cryo-transmission electron microscope (Cryo-TEM) was recorded using the Cryo-TEM (Talos F200X G2) equipped with EDS. Solid-state nuclear magnetic resonance (SSNMR) experiments were carried out at room temperature on a Bruker AVANCE NEO 600 WB spectrometer (B₀ = 14.1 T, Larmor frequency ω0 = 233.24 MHz for ⁷Li) using a 3.2 mm HXY MAS probe. The ⁷Li π/2 pulse length was 1.6 μs. Two-dimensional exchange spectroscopy (2D-EXSY) NMR was performed with a mixing time of 1 s and a spinning rate of 30 kHz. All ⁷Li chemical shifts were referenced to a 1 M aqueous LiCl solution. Time-of-flight secondary ion mass spectrometry (ToF-SIMS) was conducted on a PHI Nano ToF II system using a 30 keV Bi^3+^ primary ion beam and a 1 keV Cs⁺ sputtering beam in interlaced mode. The laser particle size analyzer (HELOS/BR-RODOS/L) was used to measure the particle size of the electrolyte. The Ta L3-edge X-ray absorption fine structure (XAFS) measurements were performed at the BL14W beamline of the Shanghai Synchrotron Radiation Facility (SSRF, Shanghai, China), employing Si(111) double-crystal monochromators. Prior to beamline measurements, samples were loaded into aluminium holders and sealed with Kapton tape. XAFS spectra were recorded at room temperature using a 4-channel Bruker 5040 silicon drift detector (SDD). For ex situ characterisation results (such as ToF-SIMS and XPS), uncycled refers to mixing NCM88 and SSE evenly and pressing directly into tablets. After cycling, the battery is assembled, and the corresponding charge and discharge times are completed, and the tablets are removed and measured.

### The ab initio molecular dynamics (AIMD) simulations

In this work, the Vienna Ab initio Simulation Package (VASP) with the projector augmented wave (PAW) method was used to perform all the structural optimisation and the ab initio molecular dynamics (AIMD) simulations. The Perdew–Burke–Ernzerhof (PBE) functional with the generalised gradient approximation (GGA) method was used for the exchange-correlation functional, in combination with the DFT-D3 correction. The initial LTOC-LaCl_3_/AlF_3_ bulk structures were built using the packmol code with a consistent ratio of atoms, with experimental values. For the interface models, the partial amorphous structure was obtained through the melting-quenching method using the Li_2_O-TaCl_5_ and LaCl_3_/AlCl_3_ heterojunction model. Once building those structures, a constant number–volume–temperature (NVT) AIMD simulation was performed at 300 K for 30 ps with a timestep of 1 fs. During the computational simulations, calculations were performed utilising only the Gamma-centred k-point mesh with a moderate plane-wave cutoff energy set to 400 eV. The energy convergence criterion for achieving self-consistency was strictly defined at 10^−^⁴  eV. Furthermore, the diffusion trajectories and mean square displacement (MSD) of Li ions were extracted and analyzed by implementing the pymatgen package.

### Ionic conductivity and activation energy, electrical conductivities, and electrochemical stability window measurements

The obtained SSE powders were cold-pressed into pellets with a diameter of 10 mm at 400 MPa for 1 min using a hydraulic press. Two stainless-steel electrodes were sputtered onto both sides of the pellets for alternating-current (AC) impedance measurements. Impedance spectra were recorded from −55 to 65 °C over a frequency range of 1 Hz to 7 MHz with an applied amplitude of 10 mV. The direct current (DC) polarisation measurements with bias voltages of 0.1–1 V were performed to determine the electrical conductivity of the samples. Electrochemical tests were conducted on a Bio‑Logic workstation in an argon-protected glovebox (H_2_O, O_2_ < 0.1 ppm). Linear sweep voltammetry (LSV) was performed using a 10 mg mixture of solid electrolyte and carbon black (7:3 wt./wt.) as the working electrode, with Li foil serving as the counter and reference electrodes. The scan rate was set at 1 mV/s. Scan from open circuit potential to 0 and 7 V, respectively.

The equivalent circuit model for the fitting of the Nyquist plot of SSEs samples; R1 is the bulk resistance, Q1 is the constant-phase element, and Q2 is the Warburg impedance.
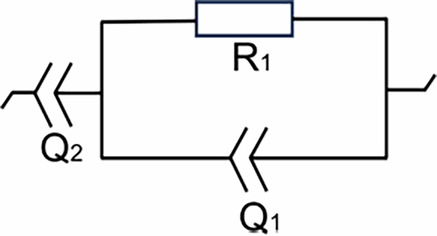


### All-solid-state battery (ASSB) preparation and battery performance measurement

For the battery performance measurements of an ASSB using the present oxyhalide SSEs, a representative LTLOC/LTOC/LTOC-5AlF_3_ was used. The positive electrode mixture was prepared by mixing LiNi_0.88_Co_0.09_Mn_0.03_O_2_ (NCM88)/LiCoO_2_(LCO) (Beijing Easpring Material Technology Co., Ltd) and LTLOC/LTOC/LTOC-5AlF_3_ in a weight ratio of NCM88/LCO: LTLOC/LTOC/LTOC-5AlF_3_ = 70: 30 or NCM88: LTLOC = 85: 15. About 50 mg of LTLOC and 50 mg of Li_6_PS_5_Cl or Li_2.5_Y_0.5_Hf_0.5_Cl_6_ (LYHC) powders as a separate layer were loaded in this order and pressed under 300 MPa to form disc-shaped pellets 10 mm in diameter. A Li-In alloy (95 wt% In) was used as the negative electrode, which was prepared by cold pressing a piece of In foil (Sino Santech Co., Ltd.) and a piece of fresh Li foil (China 679 Energy Lithium Co., Ltd.) at 200 MPa. The SUS current collectors were connected to the positive electrode and the negative electrode. The cell is 10 mm in diameter, all pressurisation processes are cold pressing, and the cells operate at a pressure of approximately 150 MPa. For a typical cell, the thicknesses of the cathode layer (11.6 mg of LiNi_0.88_Co_0.09_Mn_0.03_O_2_ composite powder), the SSE layer (50 mg of LTLOC), the sulfide layer (50 mg of Li_6_PS_5_Cl), and the anode layer (Li-In foil) are 30, 250, 450 and ~200 µm, respectively.

The ASSB preparation processes were performed in an Ar atmosphere. Charge/discharge measurements were carried out in constant current mode, terminated at 4.3/4.6 V (vs. Li^+^/Li) for charging and 2.5 V (vs. Li^+^/Li) for discharging (E (vs Li^+^/Li) = E (vs Li/LiIn) + 0.6 V, potentials were calibrated using a Li/LiIn reference electrode and converted to the Li/Li⁺ scale by adding an offset of about 0.6 V, according to the standard calibration for Li/LiIn reference electrodes in solid-state batteries. Galvanostatic charge/discharge was conducted on the LAND battery test system at RT. The Coulombic efficiency (CE) is calculated as the ratio of discharge capacity divided by the charge capacity in the preceding charge cycle. (All electrochemical tests have at least three parallel samples to ensure the accuracy of the data.)

To assemble Li|LTOC-5AlF_3_ | Li symmetric cells for constant current Li plating/stripping and critical current density testing, two Li metal foils (Φ 9 mm) were initially attached to spacers using circular marks at the centre to ensure proper electrode alignment. The obtained SE powders were cold-pressed into 10 mm diameter pellets by a hydraulic press at 400 MPa for 1 min. Then put Li metal foils on both sides, respectively. Galvanostatic charge/discharge was conducted on the NEWARE battery test system at RT.

The ASSBs used during the test are all ceramic inner pots (diameter: 10 mm), with stainless steel columns on both sides and sealed with rubber rings. The outer shell (diameter: 100 mm) is pressurised by a stainless-steel frame.

## Supplementary information

Supplementary Information

Description of Additional Supplementary Files

Supplementary Data 1

Transparent Peer Review file

## Source data

Source data file

## Data Availability

The data supporting the findings of the study are available in the Article and its Supplementary Information. The data generated in this study are provided in the Supplementary Information/Source Data/Supplementary Data. All data are available from the corresponding author upon request. Source data are provided with this paper.
