# Supplementary material for: Functional modules for enhanced amorphous composite halide solid electrolytes for low-temperature all-solid-state lithium batteries
Source: Nat Commun. 2026 May 27;17:6891. doi: 10.1038/s41467-026-71876-0 (PMC13388947; doi:10.1038/s41467-026-71876-0)
Supplement: Supplementary file 1 — Supplementary Information [file 41467_2026_71876_MOESM1_ESM.pdf]

Supplementary Information

Functional Modules for Enhanced Amorphous Composite Halide Solid Electrolytes for Low-temperature All-solid-state Lithium Batteries

Yanlong Wu [1, 2, 3], Xinmiao Wang [4], Xingyu Wang [4], Yulong Cai [3, 5], Junyi Yue [4], Simeng Zhang [4], Xiangzhen Zhu [4], Shanshan Wang [3, 6], Meng Li [3, 5], Xu Han [1, 3], Yi Duan [1], Changtai Zhao [1, 3], Rong Yang [1], Jianwen Liang* [1, 3], Xiaona Li* [4], Xueliang Sun* [4] & Jiantao Wang* [1]

[1] National Power Battery Innovation Center, China Automotive Battery Research Institute Co., Ltd., Beijing 100088, P.R. China.

[2] School of Mathematics and Science, Nanyang Institute of Technology, Nanyang, Henan 473004, PR. China

[3] GRINM (Guangdong) Institute for Advanced Materials and Technology, Foshan, Guangdong 528051, P.R. China

[4] Eastern Institute for Advanced Study, Eastern Institute of Technology, Ningbo, Zhejiang 315200, P.R. China.

[5] Beijing Advanced Innovation Center for Materials Genome Engineering, Beijing Key Laboratory for Magneto-Photoelectrical Composite and Interface Science, School of Materials Science and Engineering, University of Science and Technology Beijing, Beijing 100083, P.R. China.

[6] College of Chemistry and Environmental Engineering, Shenzhen University, Shenzhen, Guangdong 518060, P.R. China.

Corresponding emails: liangjianwen@grinm.com, xli@eitech.edu.cn, xsun@eitech.edu.cn, jiantaowang2002@126.com

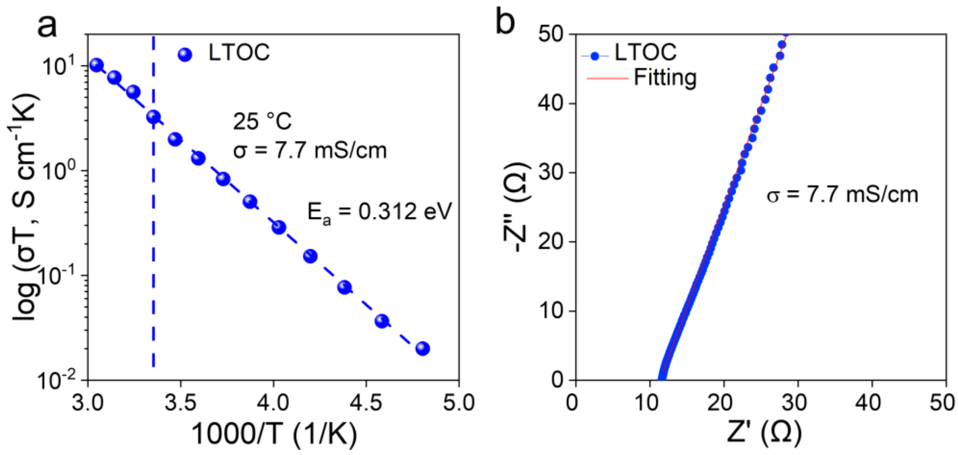

Fig. S1 | a The activation energy (E_a) and **b** EIS of LTOC at 25 °C (chsq 1.03×10^{-4}).

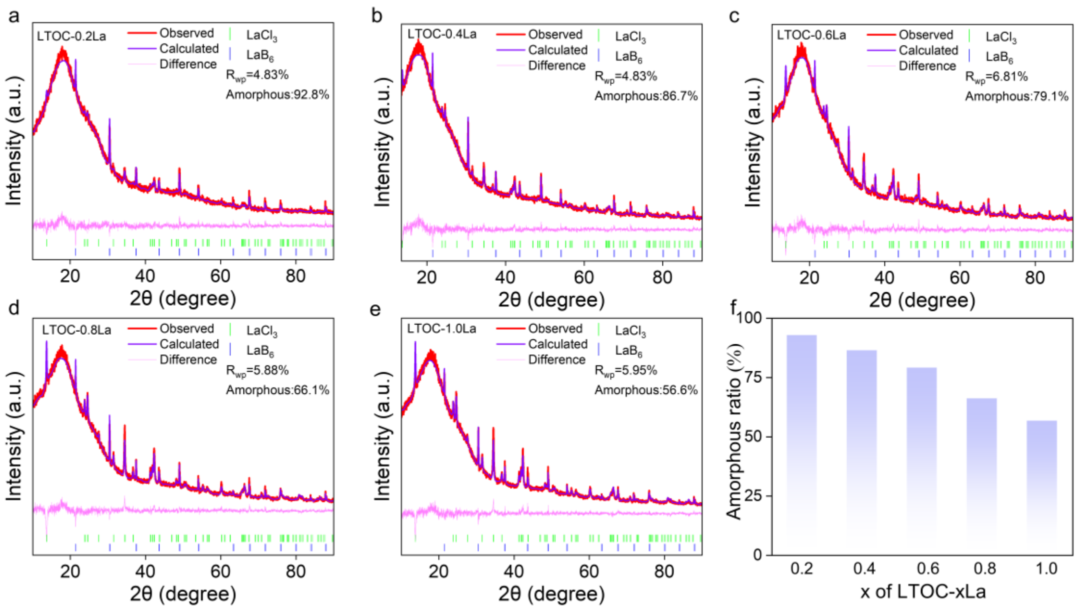

Fig. S2 | Analysis of amorphous content. a-e XRD refinement of LTOC-xLa, x = 0.2, 0.4, 0.6, 0.8 and 1.0

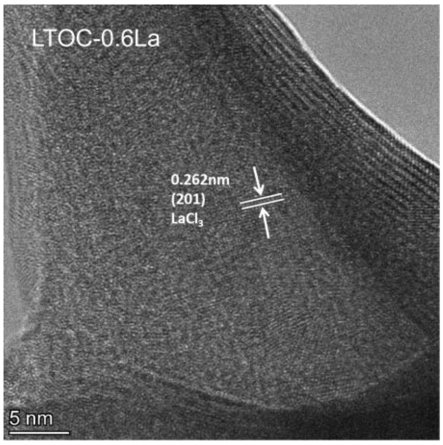

Fig. S3 | Cryo-HRTEM images of LTOC-0.6La

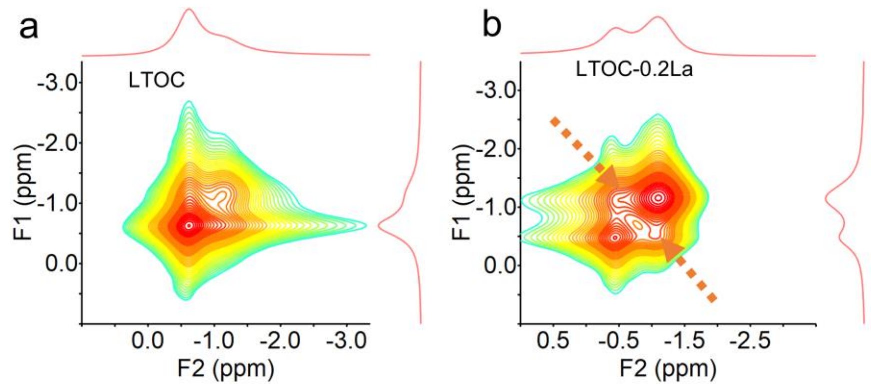

Fig. S4 | 2D EXSY NMR of LTOC and LTOC-0.2La recorded with a mixing time of 1 s and a spinning rate of 30 kHz (Relaxation Delay time is 2s). **a** 2D EXSY NMR of LTOC. **b** 2D EXSY NMR LTOC-0.2La.

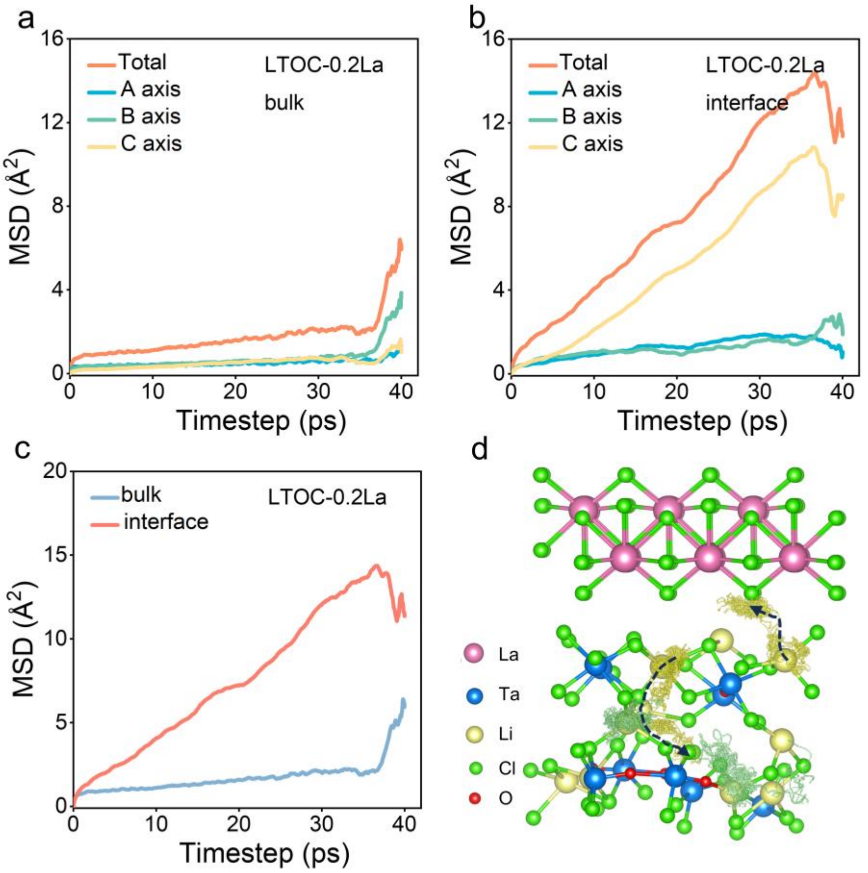

Fig. S5 | Ab initio molecular dynamics (AIMD) simulations of LTOC-0.2La. **a-c** Mean Squared Displacement of Li^+ in LTOC-0.2La. **d** The structure and potential isosurface of LTOC-0.2La are visualised using VESTA

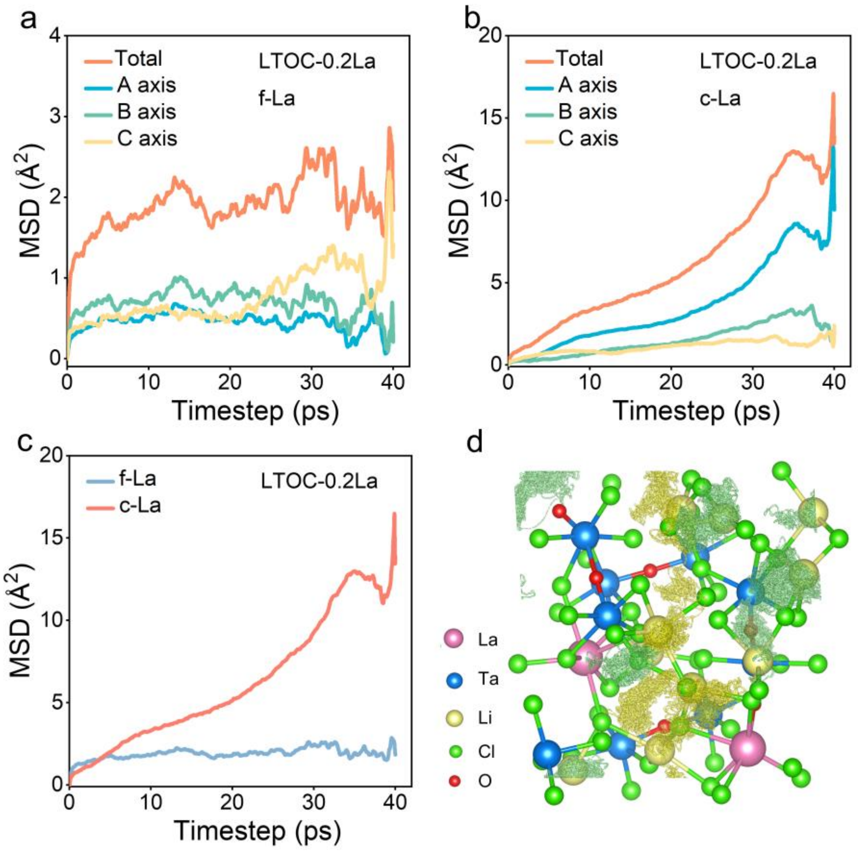

Fig. S6 | Ab initio molecular dynamics (AIMD) simulations of LTOC-0.2La. **a-c** Mean Squared Displacement of Li^+ in LTOC-0.2La. **d** The structure and potential isosurface of LTOC-0.2La are visualised using VESTA. (f-La represents being far away from La, c-La represents being close to La)

$LaCl_3$ interacts with the amorphous phase of LTOC, facilitating lithium-ion transport at the interface. Theoretical calculations further suggest that similar interfacial enhancement effects have been observed in systems such as AlOCl-nanoLiCl and ZrO_2-Li_2ZrCl_6[1, 2]. By analysing the mean square displacement, it was found that the introduction of $LaCl_3$ significantly promotes ion transport at the interface. This indicates that rapid conduction at the interface is crucial in the design of functionalized modules for electrolytes. This greatly facilitates the connectivity of lithium ion transport pathways between the bulk phase and the interface, forming a more complete, closed, and multidimensional lithium ion transport channel, which effectively reduces the migration barrier of lithium ions and thus enhances lithium-ion conductivity (Fig. S5).

Second, Characterisation results confirm that $LaCl_3$ doping effectively enhances the ionic conductivity. The doping effect of $LaCl_3$, particularly the introduction of La^{3+}, plays a crucial role in enhancing ionic conductivity. However, excessive La^{3+} introduction may lead to $LaCl_3$ precipitation, which could reduce ionic conductivity.

Theoretical calculations reveal that the introduction of La^{3+} alters the local environment of LTOC. The doping effect of cations leads to a differentiation in the local lithium-ion migration energy barrier of the electrolyte. The vibrational frequency of lithium ions in channels adjacent to La atoms is markedly higher than that of ions located further away, suggesting a reduced migration energy barrier in these regions, thereby facilitating ion transport (Fig. S6). Nevertheless, even with the introduction of $0.8LaCl_3$ (LTOC-0.8La), which exhibits an ionic conductivity of 8.7 mS/cm, this value remains higher than that of reported amorphous LTOC (7 mS/cm), indicating the persistence of the doping effect of $LaCl_3$. Thus, ion conductivity is closely related to doping.

In this work, the introduction of $LaCl_3$ serves merely as an example to illustrate the feasibility of the functional module design concept. The mechanism by which $LaCl_3$ enhances ionic conductivity is specific to the LTOC-x$LaCl_3$ system, and the effects of other functional modules on ionic transport must be evaluated individually. Overall, the incorporation of $LaCl_3$ effectively improves ionic conductivity through the synergistic effects of promoting lithium-ion transport at the interface and the doping effect of La^{3+}.

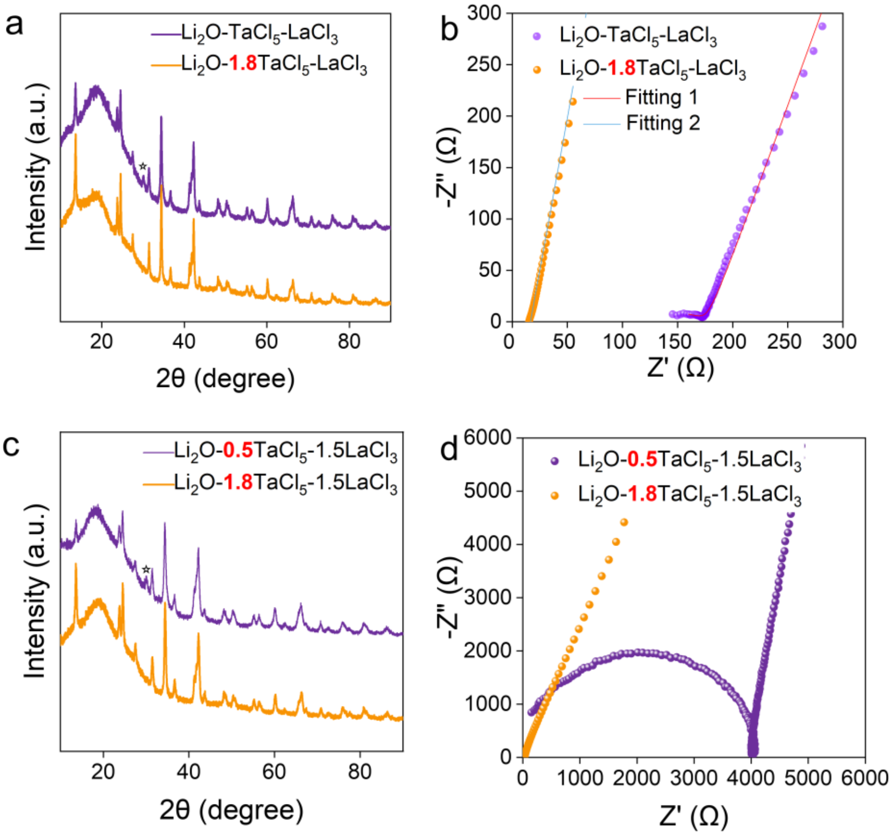

Fig. S7 | a XRD patterns of Li_2O-x$TaCl_5$-$LaCl_3$ (x = 1 and 1.8). **b** EIS of Li_2O-x$TaCl_5$-$LaCl_3$ (x = 1 and 1.8) (chsq 1.25×10^{-4} and 1.67×10^{-4}). **c** XRD patterns of Li_2O-x$TaCl_5$-

1.5$LaCl_3$ (x = 0.5 and 1.8). **d** EIS of Li_2O-x$TaCl_5$-1.5$LaCl_3$ (x = 0.5 and 1.8) at 25 °C.

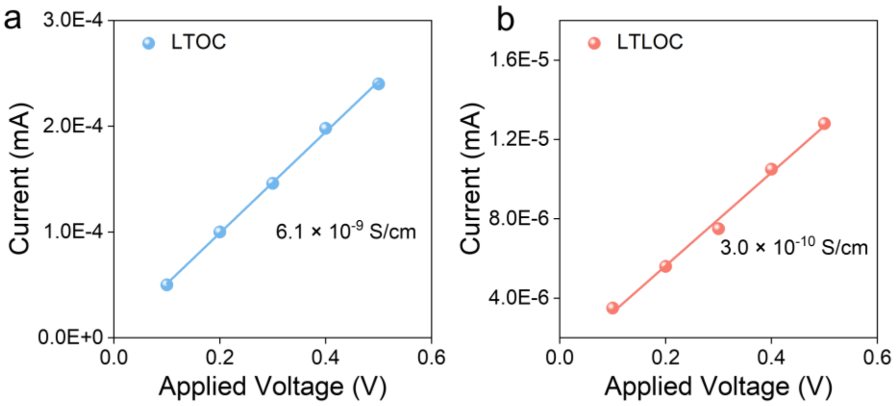

Fig. S8 | The transient current behaviour under DC bias for the LTOC/LTLOC with stainless steel (SS) electrodes. **a** The transient current behaviour under DC bias for the LTOC with stainless steel (SS) electrodes. **b** The transient current behaviour under DC bias for the LTLOC with stainless steel (SS) electrodes at 25 °C.

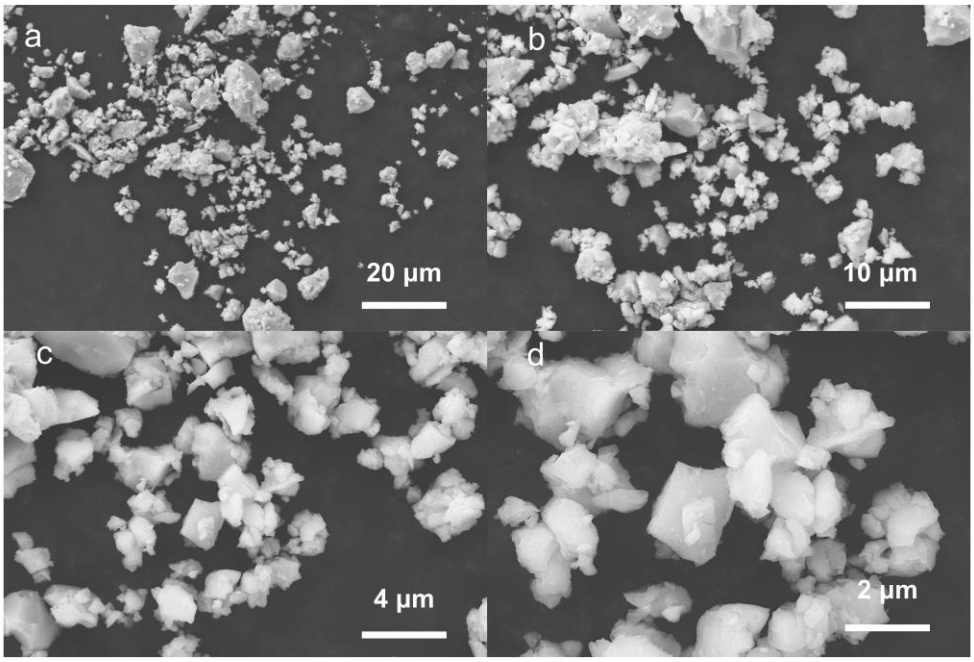

Fig. S9 | SEM images with different magnifications of LTOC-0.2La SSEs. **a** x1000; **b** x2000; **c** x5000; **d** x10000

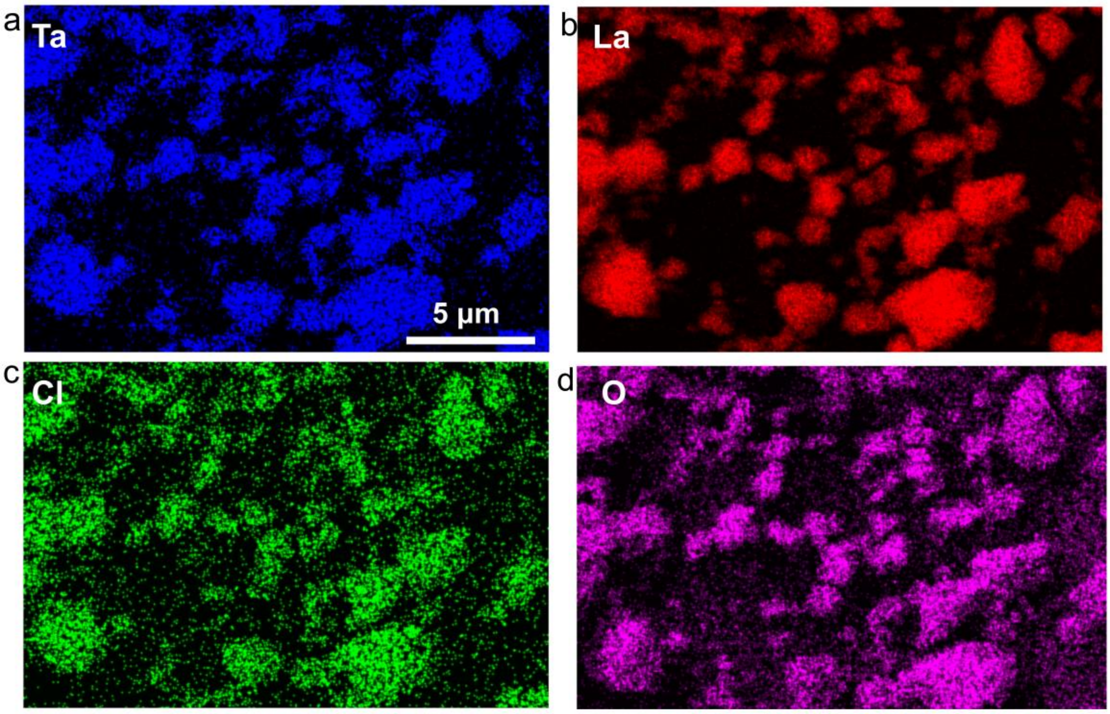

Fig. S10 | EDS element mapping of the image of LTOC-0.2La. **a** Ta element mapping. **b** La element mapping. **c** Cl element mapping. **d** O element mapping.

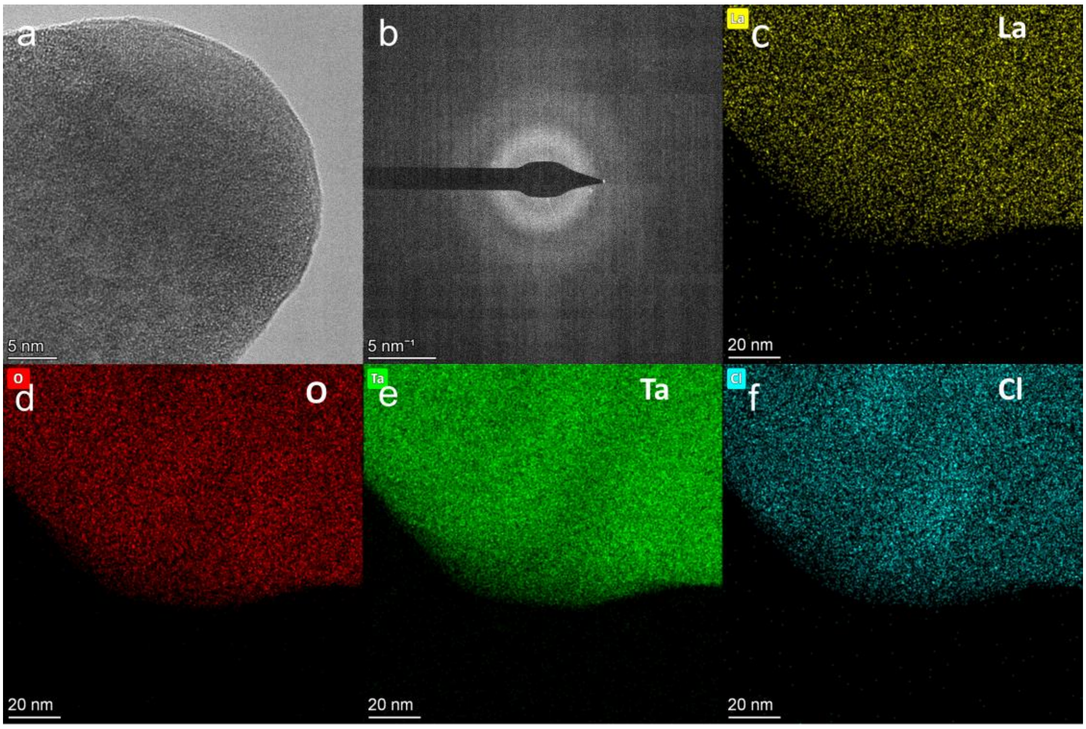

Fig. S11 | TEM images and element mapping of the image of LTLOC. **a, b** TEM image of the LTLOC. **c-f** The element mapping of the image of LTLOC

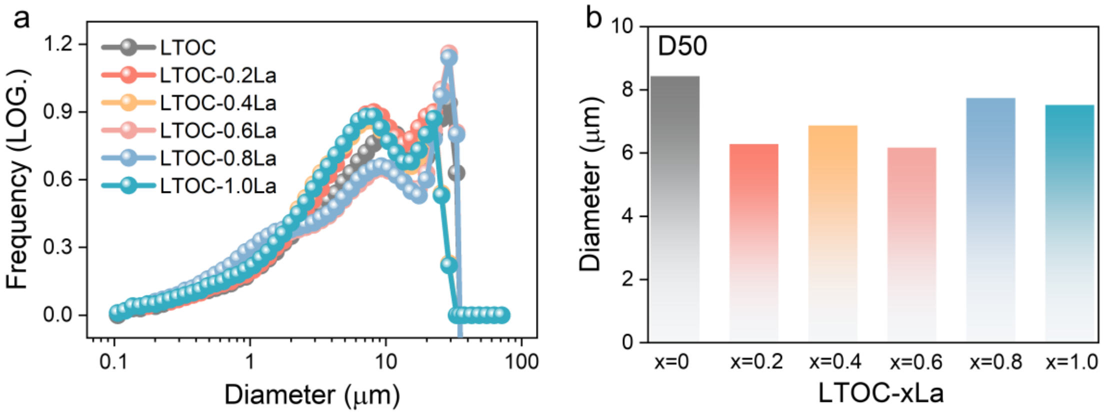

Fig. S12 | The particle size distribution of LTOC-xLa (x = 0, 0.2, 0.4, 0.6, 0.8, 1.0) SSEs. **a** The diameter of LTOC-xLa (x = 0, 0.2, 0.4, 0.6, 0.8, 1.0) SSEs. **b** Comparison of particle size distribution of LTOC-xLa (x = 0, 0.2, 0.4, 0.6, 0.8, 1.0) SSEs.

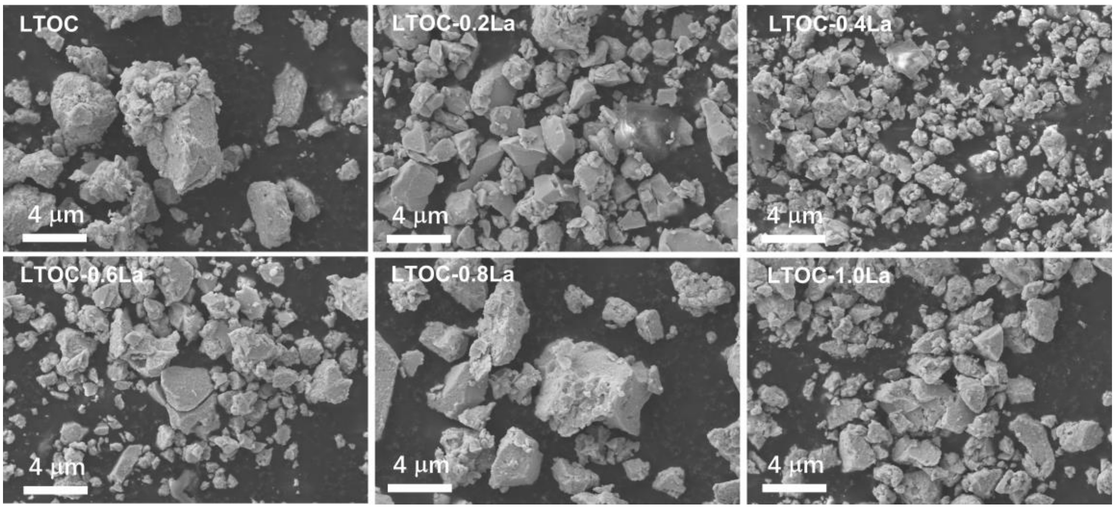

Fig. S13 | SEM images of LTOC-xLa (x = 0, 0.2, 0.4, 0.6, 0.8, 1.0) SSEs

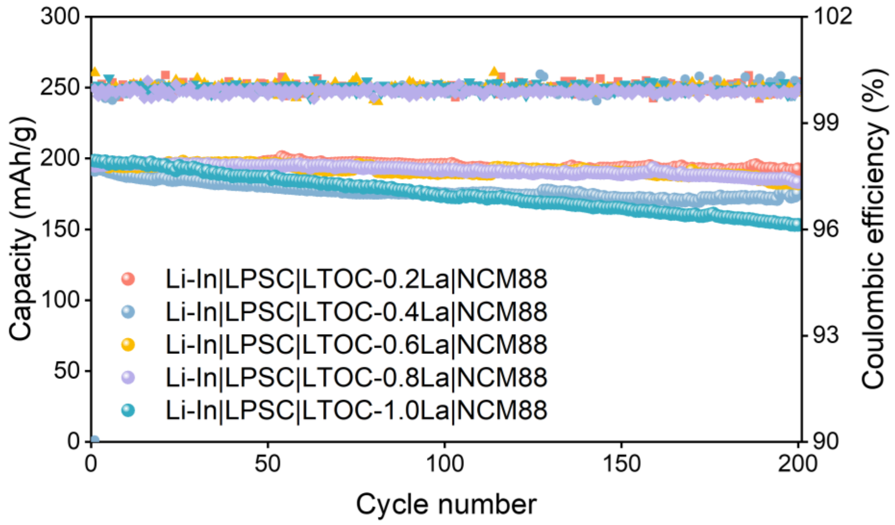

Fig. S14 | Electrochemical performance of Li-In|LPSC|LTOC-xL|NCM88 at 40 mA/g.

The relationship between filler loading and electrolyte particle size remains unclear; however, it plays a critical role in determining the physical contact and chemical performance between the electrolyte and the positive electrode in a composite electrode. To investigate this, we characterized a series of solid-state electrolytes, LTOC-xLa (x = 0.2, 0.4, 0.6, 0.8, and 1.0), with varying proportions of functional filler ($LaCl_3$), focusing on their particle size distribution, morphology, Young's modulus, and the electrochemical performance of all-solid-state batteries assembled under identical conditions using these electrolytes. This aims to comprehensively understand the effects of different proportions of functional fillers on the solid-state electrolytes themselves, including particle size, morphology, and Young's modulus, as well as their impact on the contact and conductivity at the composite electrode interface. The particle size distribution indicates that the particle size of LTOC is slightly larger, and the introduction of $LaCl_3$ results in a slight reduction in particle size; however, the overall difference is minor, with D50 values ranging between 6-9 μm (Fig. S12). SEM observations reveal that the particles are irregular in shape, with no significant differences in morphology (Fig. S12, 13).

As the value of x increases, the Young's modulus exhibits a trend of first decreasing and then increasing (Table S4). Additionally, the initial discharge specific capacity of the all-solid-state battery assembled with the electrolyte is close to 200 mAh/g (20 mA/g). After 200 cycles, the capacity retention rate shows slight differences, with Li-In|LPSC|LTOC-xL|NCM88 demonstrating the poorest capacity retention (Fig. S14). Although the experimental data do not yet allow definitive conclusions to be drawn, they suggest that the molar ratio of functional modules exerts a measurable influence on the properties of the electrolyte and the performance of all-solid-state batteries employing it. This represents a promising direction for further investigation.

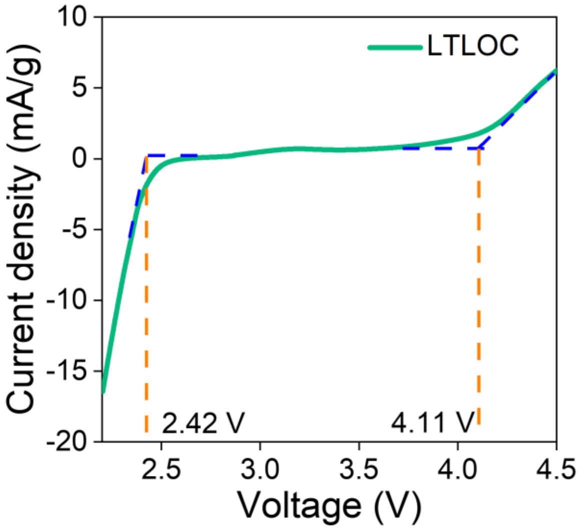

Fig. S15 | The LSV curve of the Li|LTLOC|LTLOC+Carbon cell at 1 mV/s

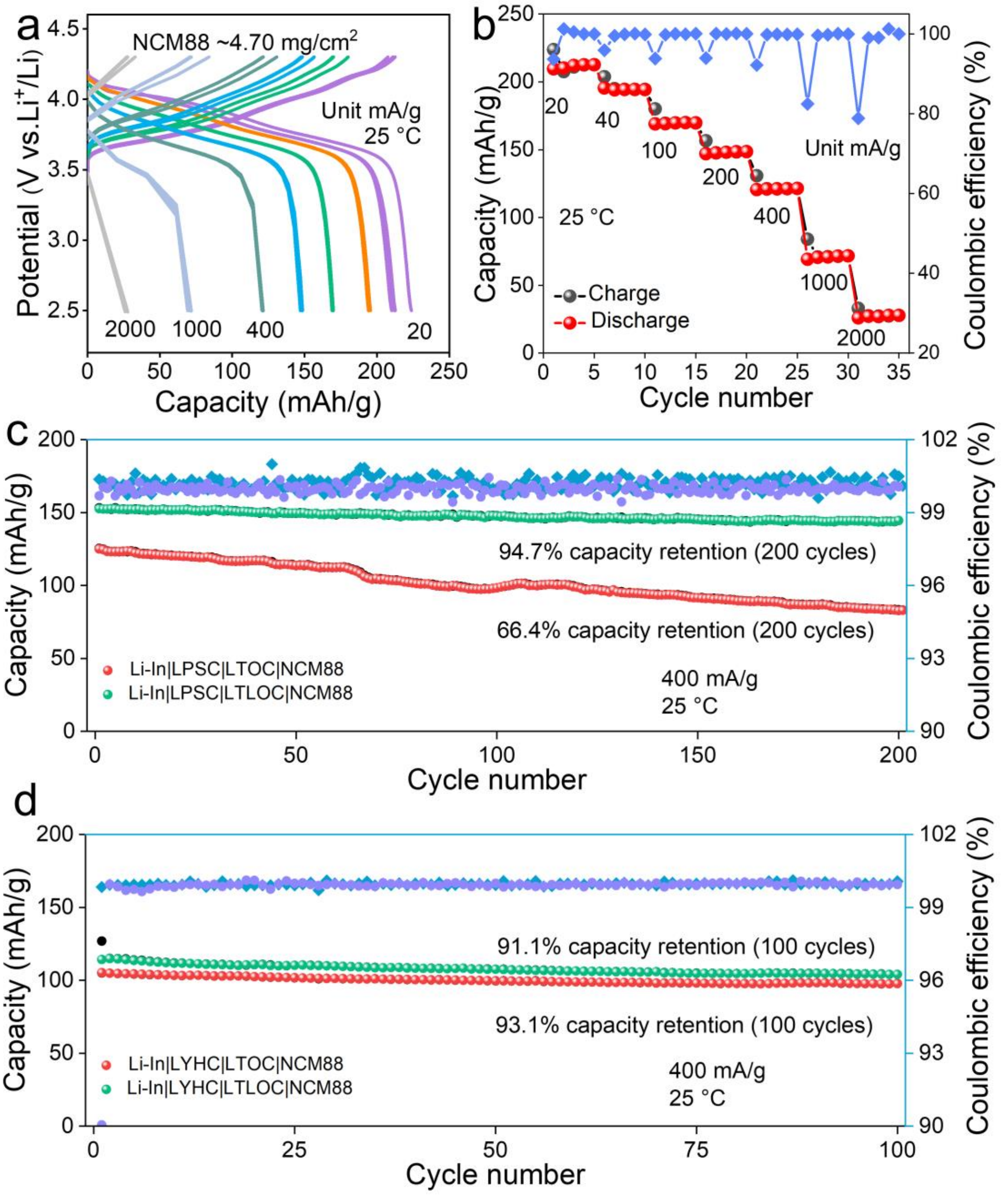

Fig. S16 | Electrochemical performance of Li-In|LPSC (LYHC)|LTLOC|NCM88 and Li-In|LPSC (LYHC)|LTOC|NCM88 at 25 °C. **a** Discharge/charge curves and **b** Cycling performance of the Li-In|LPSC (LYHC)|LTOC|NCM88 ASSB at specific currents (20, 40, 100, 200, 400, 1,000, and 2,000 mA/g). **c**, **d** Long-term cycling performance and coulombic efficiency at 400 mA/g.

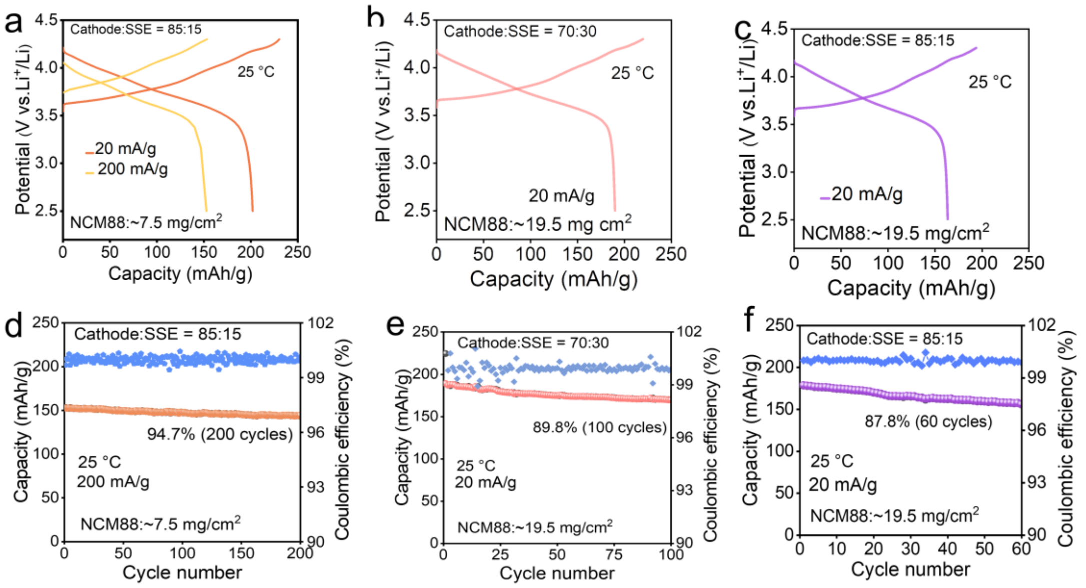

Fig. S17 | Electrochemical performance of ASSB of Li-In|LPSC|LTLOC|NCM88 ASSB at 4.3 V (vs.Li^+/Li). **a** Discharge/charge curves of LTLOC|NCM88 ASSB (85 wt% NCM88 and 15 wt% SSE, NCM88: ~7.5 mg/cm^2). **b** Discharge/charge curves of LTLOC|NCM88 ASSB (70 wt% NCM88 and 30 wt% SSE, NCM88: ~19.5 mg/cm^2). **c** Discharge/charge curves of LTLOC|NCM88 ASSB (85 wt% NCM88 and 15 wt% SSE, NCM88: ~19.5 mg/cm^2). **d** Long-term cycling performance and coulombic efficiency of LTLOC|NCM88 ASSB (85 wt% NCM88 and 15 wt% SSE, NCM88: ~7.5 mg/cm^2). **e** Long-term cycling performance and coulombic efficiency of LTLOC|NCM88 ASSB (70 wt% NCM88 and 30 wt% SSE, NCM88: ~19.5 mg/cm^2). **f** Long-term cycling performance and coulombic efficiency of LTLOC|NCM88 ASSB (85 wt% NCM88 and 15 wt% SSE, NCM88: ~19.5 mg/cm^2)

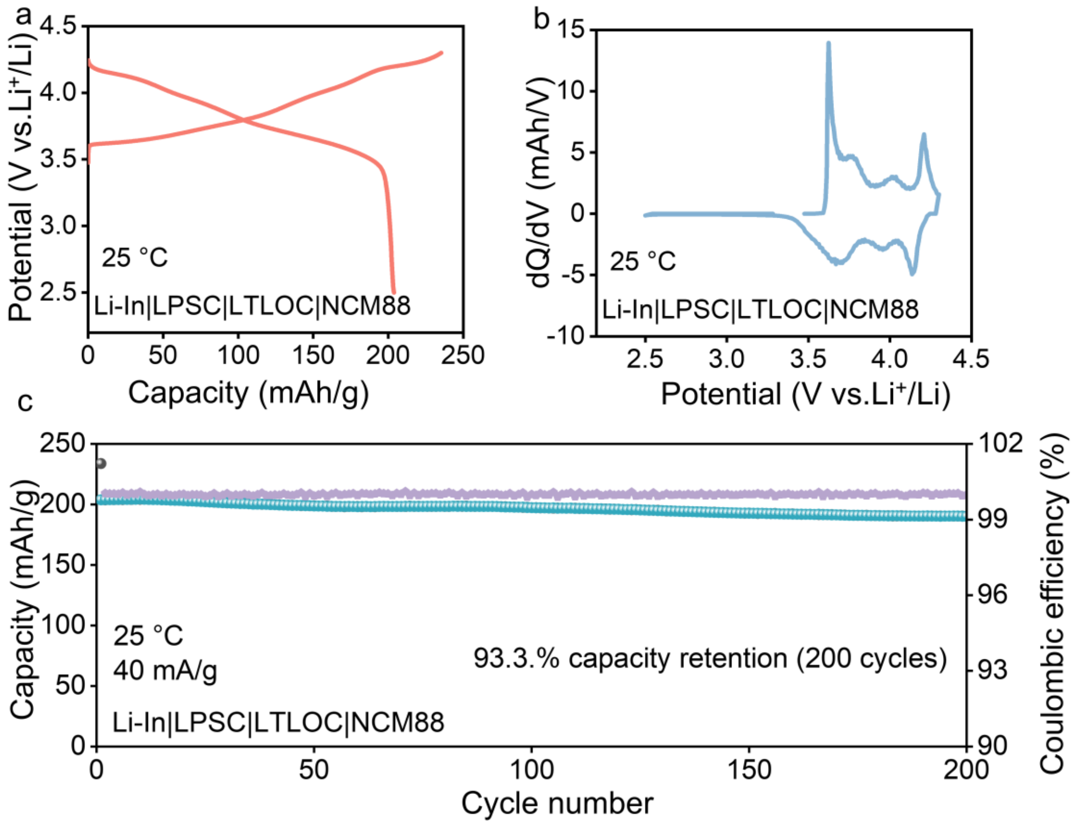

Fig. S18 | a Discharge/charge curves of the Li-In|LPSC|LTLOC|NCM88 ASSB at 0.2 C. b DQ/dV curves of the Li-In|LPSC|LTLOC|NCM88 ASSB at 40 mA/g. c Long-term cycling performance and coulombic efficiency at 40 mA/g of the Li-In|LPSC|LTLOC|NCM88 ASSB at 25 °C. (The XPS, TOF-SIMS, and SEM data of the ASSB after 200 cycles at a rate of 40 mA/g (discharge specific capacity of approximately 200 mAh/g).

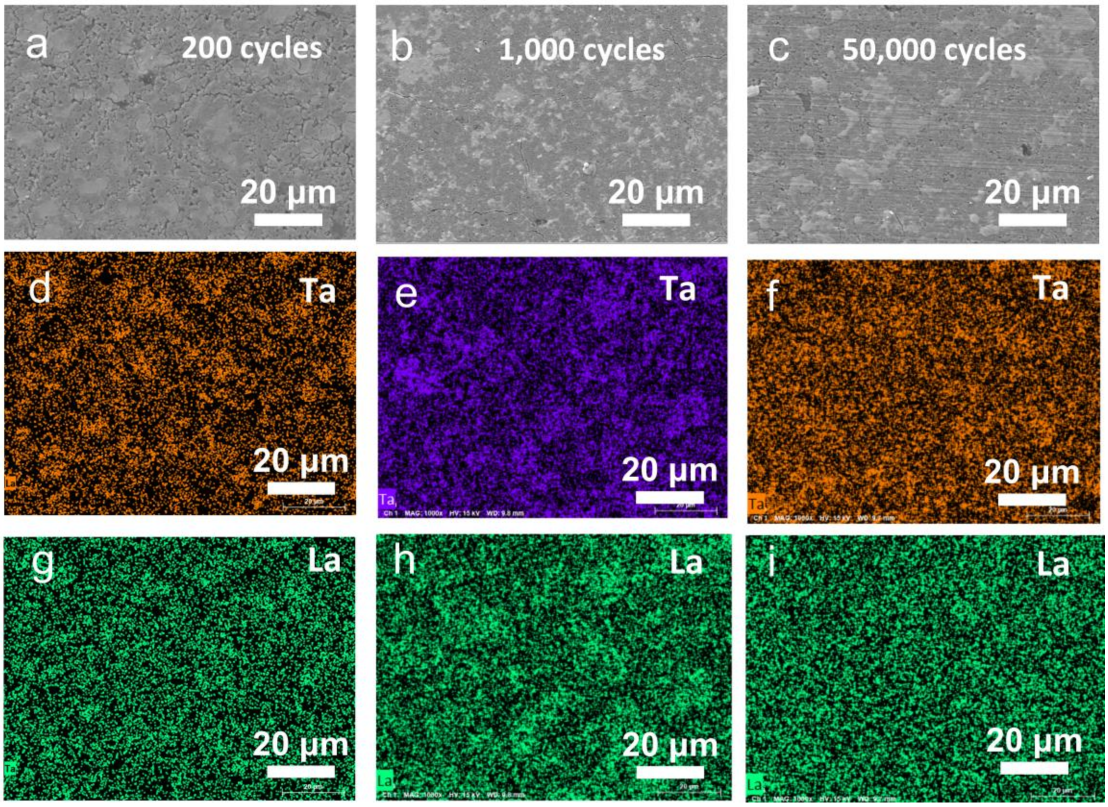

Fig. S19 | SEM and EDS images of LTLOC|NCM88 ASSB after 200, 1,000, and 50,000 cycles. **a** The SEM image of LTLOC|NCM88 ASSB after 200 cycles. **b** The SEM image of LTLOC|NCM88 ASSB after 1,000 cycles. **c** The SEM image of LTLOC|NCM88 ASSB after 50,000 cycles. **d, g** The EDS images of **a**. **e, h** The EDS images of **b**. **f, i** The EDS images of **c**. (**a, d,** and **g** at a rate of 40 mA/g (discharge specific capacity of approximately 200 mAh/g)

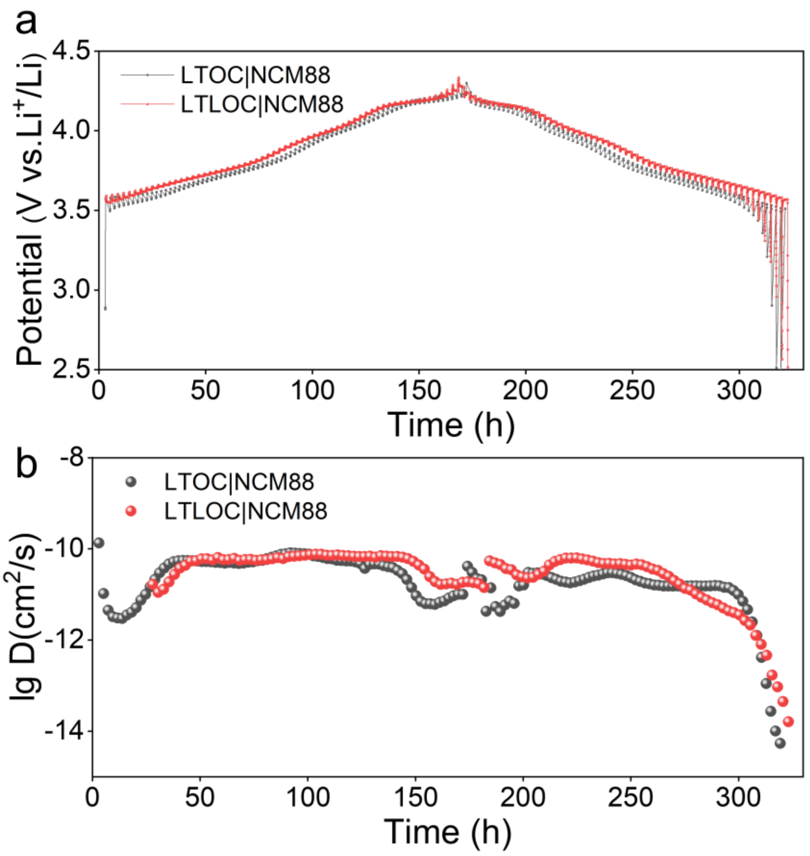

Fig. S20 | a The GITT curves of LTOC|NCM88 and LTLOC|NCM88 ASSBs. **b** The Li^+ diffusion coefficient of LTOC|NCM88 and LTLOC|NCM88 ASSBs. Cycle under the condition that the specific current is 20 mA/g, calculate the current based on the active material, and then charge for 1 hour and let stand for 3 hours under this current to allow ion diffusion in the system to reach an equilibrium state, and collect data.

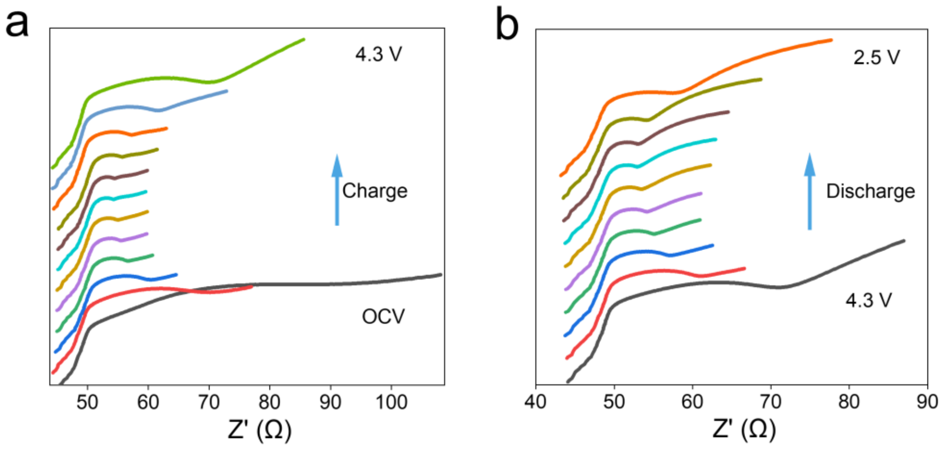

Fig. S21 | The impedance change during charging and discharging of LTLOC|NCM88 ASSB. **a** The impedance change during charging. **b** The impedance change during discharging

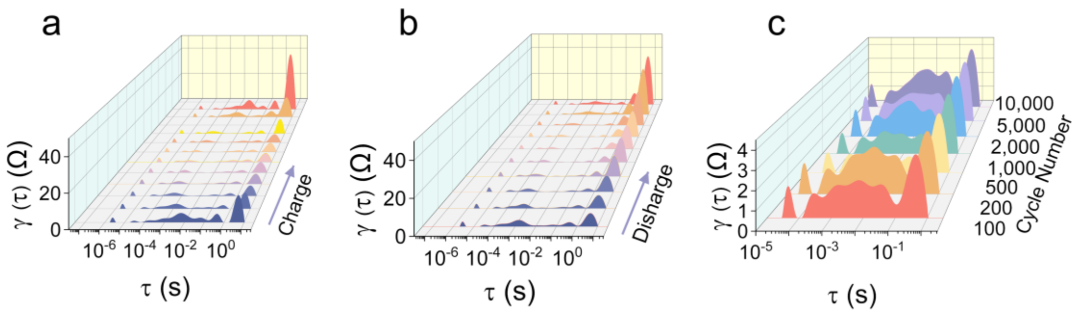

Fig. S22 | a DRT is calculated from the EIS measurements in charge. **b** DRT is calculated from EIS measurements during discharge. **c** DRT calculated from EIS measurements at different cycles

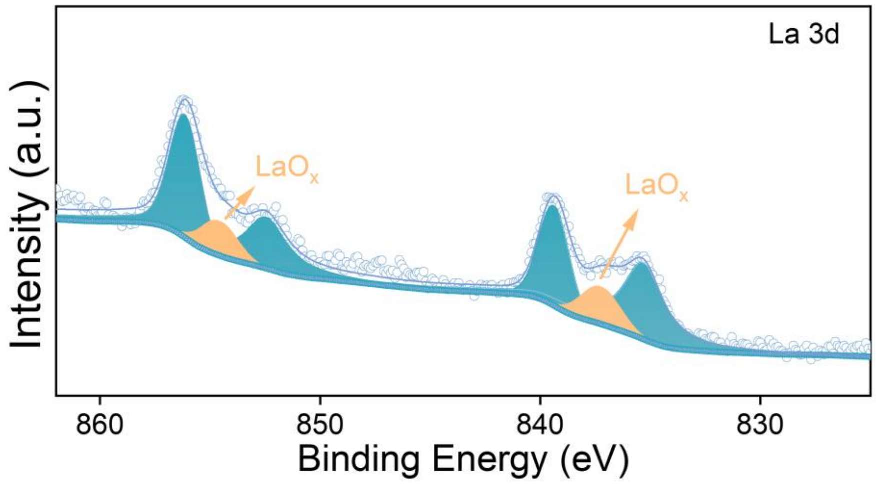

Fig. S23 | XPS spectra of La 3d after 200 cycles at a rate of 40 mA/g (discharge specific capacity of approximately 200 mAh/g)

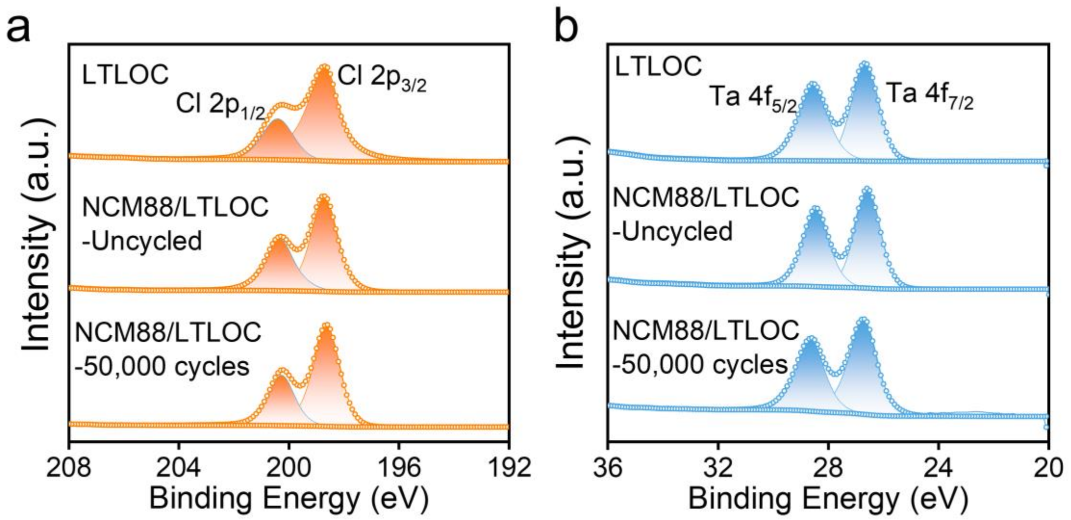

Fig. S24 | a XPS spectrum of Cl at uncycled and after 50,000 cycles. **b** XPS spectrum of Ta at uncycled and after 50,000 cycles

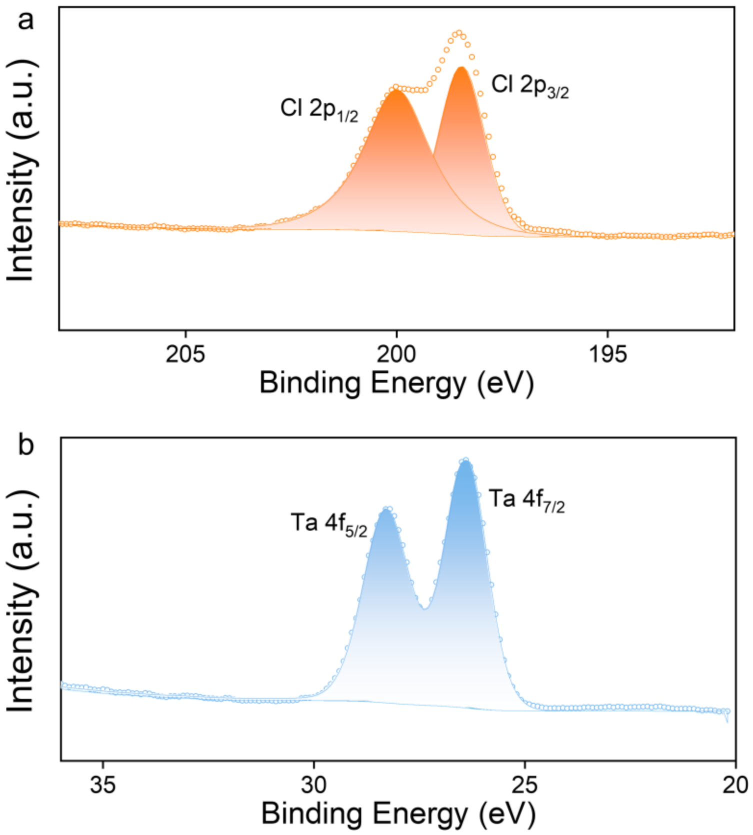

Fig. S25 | a XPS spectrum of Cl after 200 cycles at a rate of 40 mA/g. **b** XPS spectrum of Ta after 150 cycles at a rate of 40 mA/g (discharge specific capacity of approximately 200mAh/g)

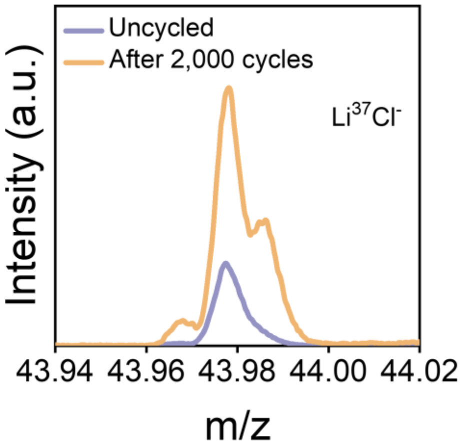

Fig. S26 | ToF-SIMS surface analysis results of Li^{37}Cl$^-$ at uncycled and after 2,000 cycles

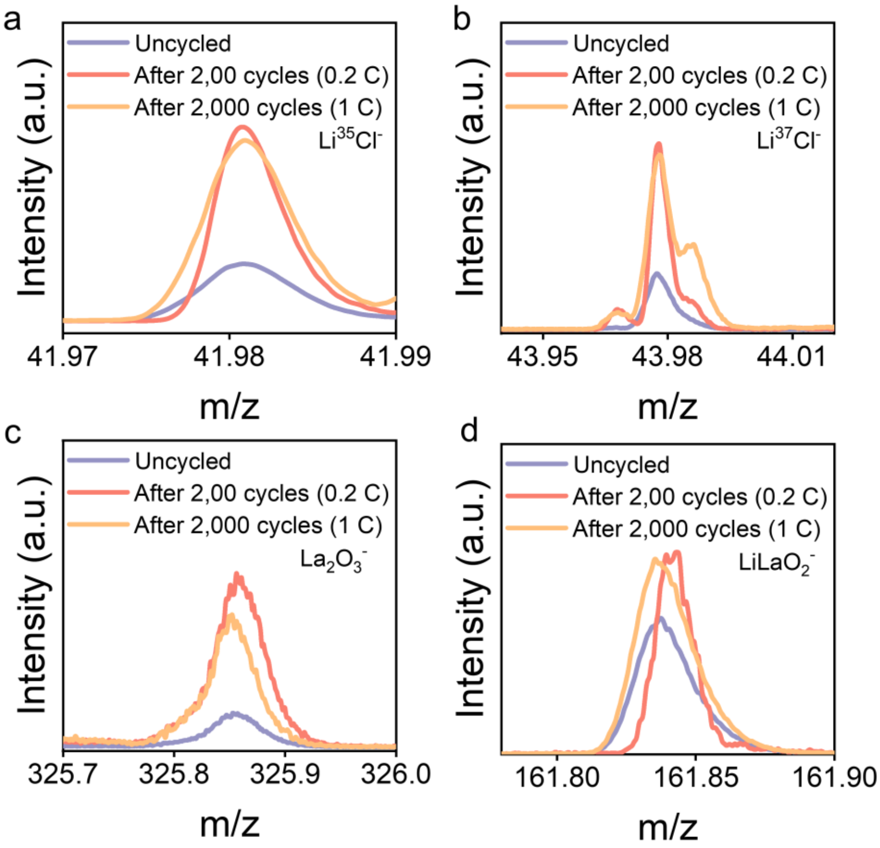

Fig. S27 | ToF-SIMS surface analysis results of **a** $Li^{35}Cl^-$, **b** $Li^{37}Cl^-$, **c** $La_2O_3^-$, **d** $LiLaO_2^-$ at a rate of 40 mA/g (discharge specific capacity of approximately 200 mAh/g).

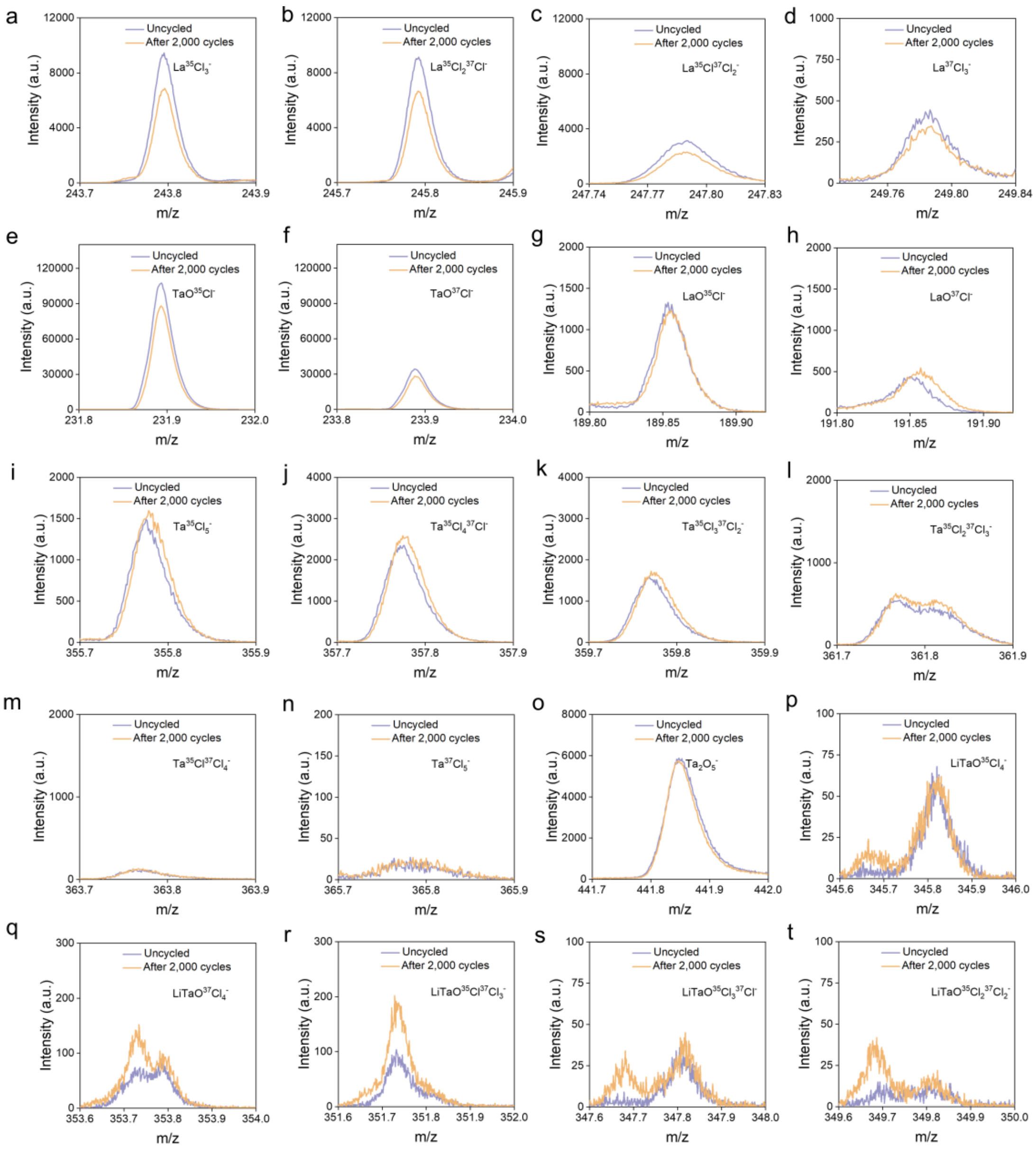

Fig. S28 | ToF-SIMS surface analysis results of **a-d** $LaCl_3^-$, **e-f** $TaOCl^-$, **g-h** $LaOCl^-$, **i-n** $TaCl_5^-$, **o** $Ta_2O_5^-$, and **p-t** $LiTaOCl_4^-$ at uncycled and after 2,000 cycles

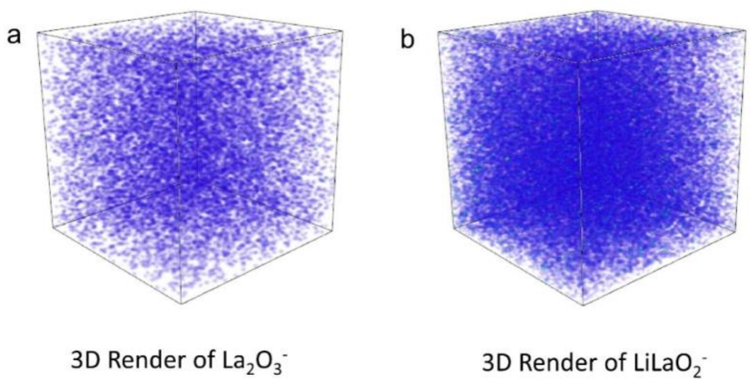

Fig. S29 | ToF-SIMS surface analysis results of 3D render: **a** $La_2O_3^-$ and **b** $LiLaO_2^-$

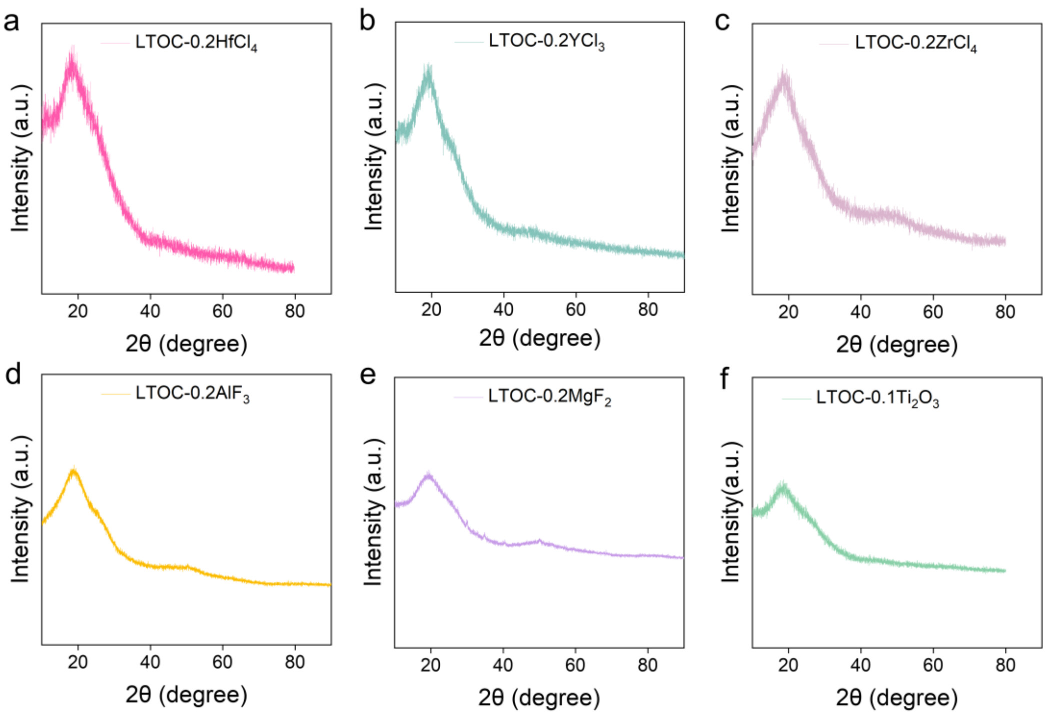

Fig. S30 | XRD patterns: **a** LTOC-0.2$HfCl_4$, **b** LTOC-0.2YCl_3, **c** LTOC-0.2$ZrCl_4$, **d** LTOC-0.2AlF_3, **e** LTOC-0.2MgF_2, and **f** LTOC-0.1Ti_2O_3

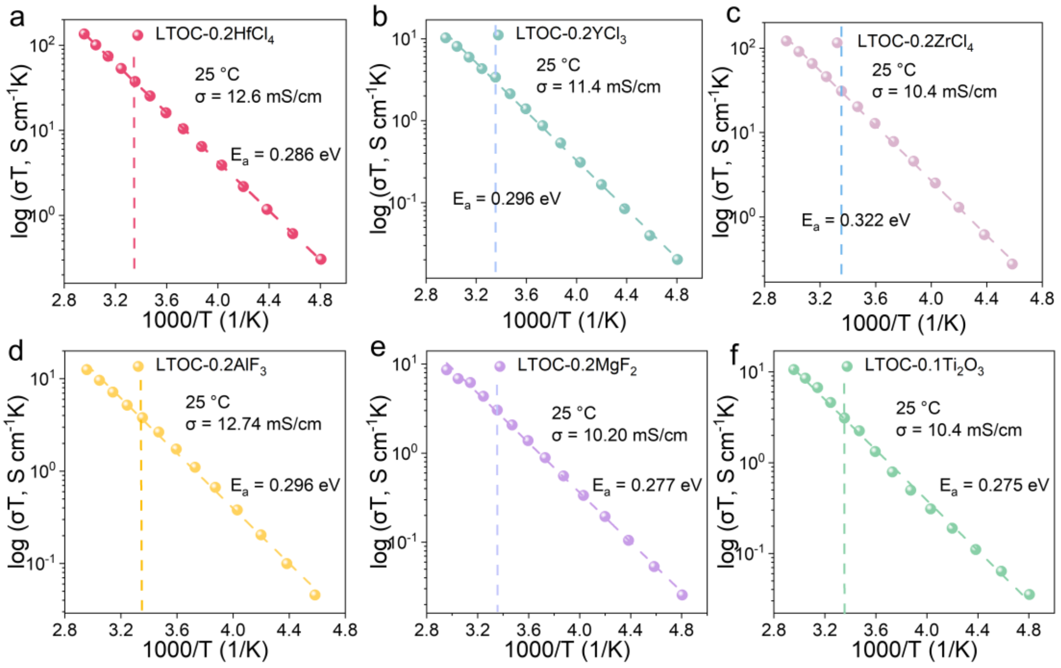

Fig. S31 | Arrhenius plots: **a** LTOC-0.2$HfCl_4$, **b** LTOC-0.2YCl_3, **c** LTOC-0.2$ZrCl_4$, **d** LTOC-0.2AlF_3, **e** LTOC-0.2MgF_2, and **f** LTOC-0.1Ti_2O_3 at 25 °C.

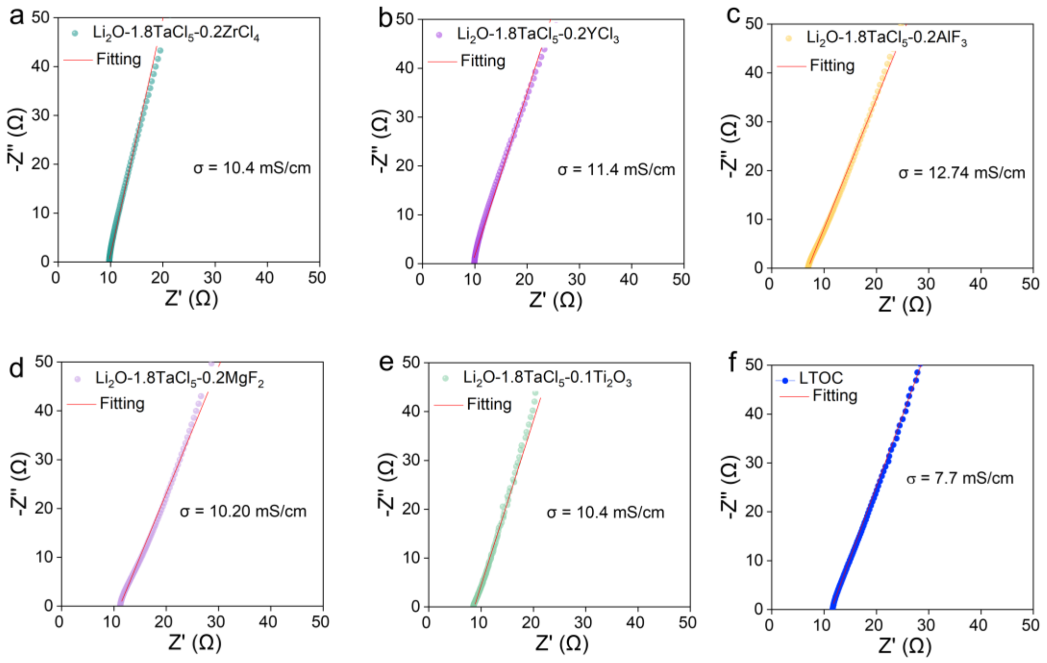

Fig. S32 | The EIS: a LTOC-0.2$HfCl_4$ (chsq 6.53×10^{-4}), **b** LTOC-0.2YCl_3 (chsq 3.01×10^{-4}), **c** LTOC-0.2$ZrCl_4$ (chsq 3.95×10^{-4}), **d** LTOC-0.2AlF_3 (chsq 8.44×10^{-4}), **e** LTOC-0.2MgF_2 (chsq 5.32×10^{-4}), and **f** LTOC-0.1Ti_2O_3 (chsq 6.11×10^{-4}) at 25 °C.

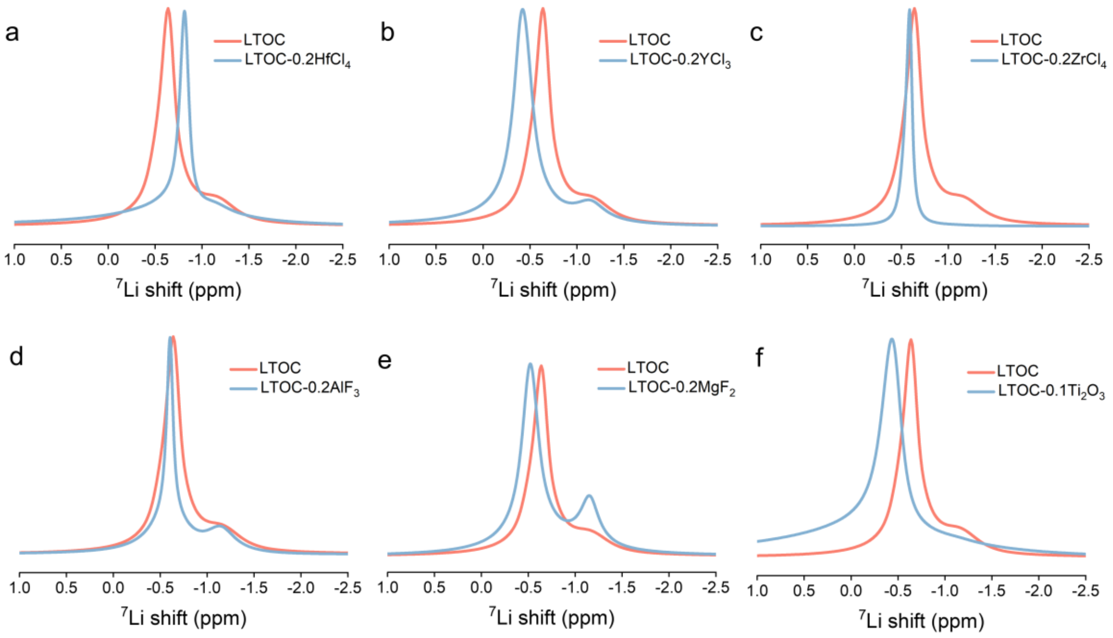

Fig. S33 | ^{7}Li 1D NMR spectra: **a** LTOC-0.2$HfCl_4$, **b** LTOC-0.2YCl_3, **c** LTOC-0.2$ZrCl_4$, **d** LTOC-0.2AlF_3, **e** LTOC-0.2MgF_2, and **f** LTOC-0.1Ti_2O_3

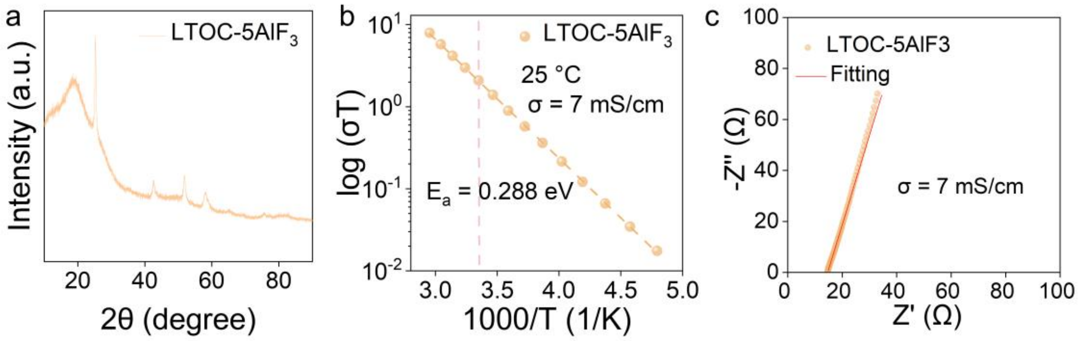

Fig. S34 | a XRD patterns of LTOC-5AlF_3. **b** Arrhenius plots of LTOC-5AlF_3. **c** EIS of LTOC-5AlF_3 at 25 °C (chsq 1.57×10^{-4}).

The ionic conduction mechanism of LTOC-5AlF_3

The relatively high proportion of AlF_3 introduced, coupled with its limited solubility in the amorphous phase, renders the presence of crystalline components inevitable. By comparing the characteristic peaks from XRD with standard PDF cards, it can be inferred that the crystalline phase is AlF_3 (Fig. S35-37). However, the proportion of the crystalline phase is slightly lower than the proportion of AlF_3 in the raw materials during synthesis, indicating that a portion of AlF_3 has formed an amorphous phase, while the other AlF_3 exists separately (Fig.S38). To verify this conclusion, we directly observed the presence of AlF_3 in LTOC-5AlF_3 through TEM (Fig.S39). These findings indicate that the functional modules preferentially integrate into the amorphous phase; however, when their proportion exceeds the solubility limit of the amorphous matrix, a crystalline phase begins to precipitate.

We conducted a comprehensive analysis of the interaction between AlF_3 as a functional module and the amorphous part of LTOC using first-principles calculations. The interaction between the functional modules and the amorphous phase not only involves doping effects but also promotes interfacial conduction.

Theoretical calculations indicate that the introduction of AlF_3 modifies the local environment of LTOC. The cation doping effect results in a differentiation in the local lithium-ion migration energy barrier within the electrolyte. The vibration frequency of lithium ions in the channel adjacent to Al atoms is significantly higher than that of lithium ions situated farther from Al atoms. This observation suggests that the migration energy barrier for lithium ions in this position is lower, thereby facilitating ionic transport (Fig. S40).

To eliminate the interference of F ions on the interface, we constructed an interface

model of LTOC-$AlCl_3$. Similar to LTOC-0.2La, it was found to enhance the transport. This highlights the importance of rapid conduction at the interface in designing functionalized modules for electrolytes. This greatly facilitates the connectivity of lithium-ion transport pathways between the bulk phase and the interface, forming a more complete, closed, and multidimensional lithium-ion transport channel, which effectively reduces the migration barrier of lithium ions and thus enhances lithium-ion conductivity at the interface (Fig. S41).

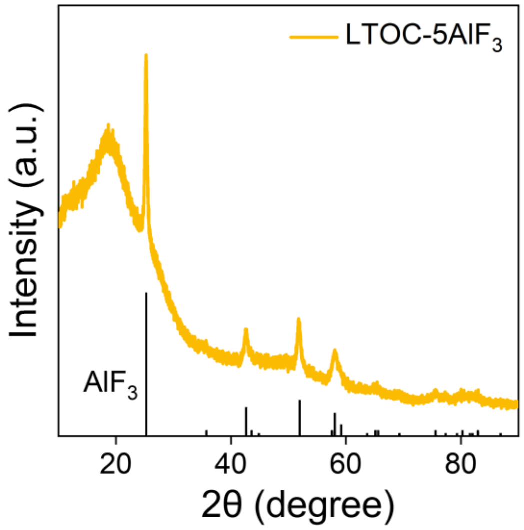

Fig. S35 | XRD patterns of LTOC-5AlF_3

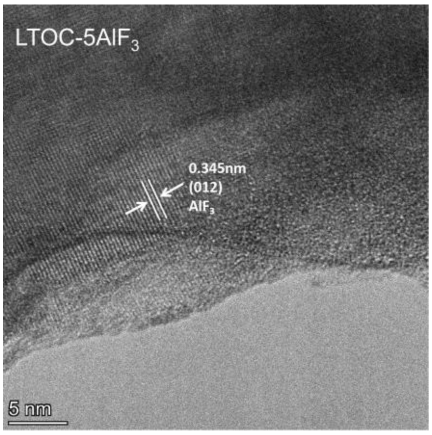

Fig. S36 | TEM images of LTOC-5AlF_3

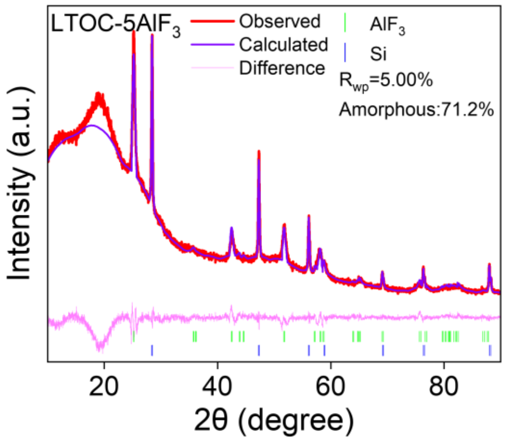

Fig. S37 | XRD refinement of LTOC-5AlF_3

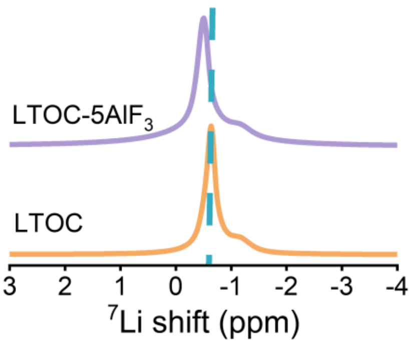

Fig. S38 | ^{7}Li 1D NMR spectra of LTOC and LTOC-5AlF_3

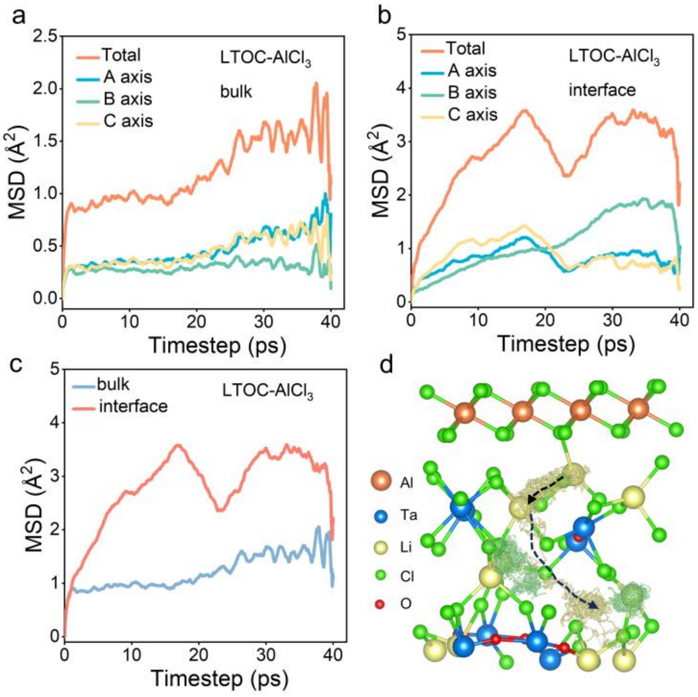

Fig. S39 | Ab initio molecular dynamics (AIMD) simulations of LTOC-$AlCl_3$. **a-c** Mean Squared Displacement of Li^+ in LTOC-$AlCl_3$. **d** The structure and potential isosurface

of LTOC-AlCl3 are visualised using VESTA

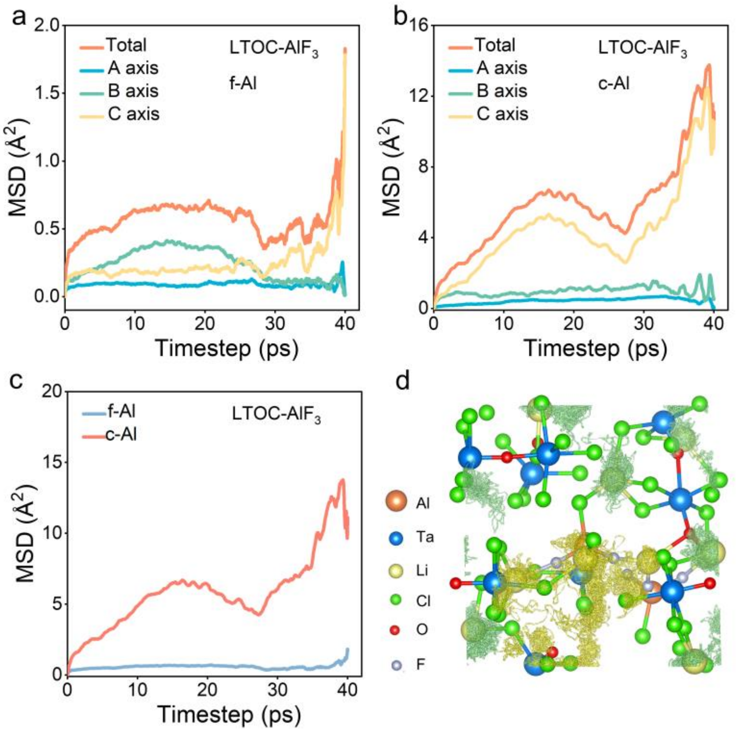

Fig. S40 | Ab initio molecular dynamics (AIMD) simulations of LTOC-$AlCl_3$. **a-c** Mean Squared Displacement of Li^+ in LTOC-$AlCl_3$. **d** The structure and potential isosurface of LTOC-$AlCl_3$ are visualised using VESTA. (f-Al represents being far away from Al, c-Al represents being close to Al)

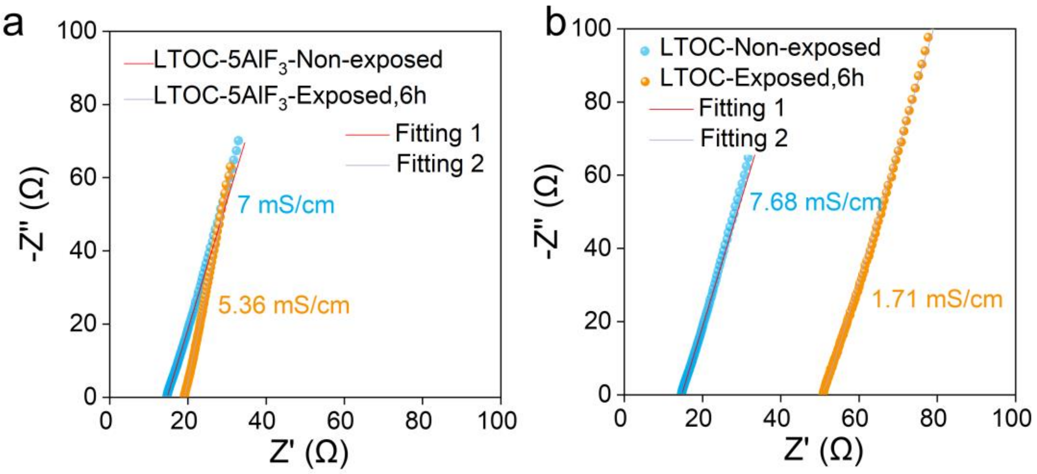

Fig. S41 | a Comparison of the EIS of LTOC-5AlF_3 before and after exposure (dew point of -30 °C) (chsq 7.20×10^{-4} and 4.33×10^{-4}). **b** Comparison of the EIS of LTOC before and after exposure (dew point of -30 °C) (chsq 9.06×10^{-4} and 5.21×10^{-4}).

To demonstrate the significant improvement in humidity stability compared to LTOC, we compared the ion conductivity of LTOC-5AlF_3 after a two-hour exposure to

a higher dew point of -10 °C. The results indicate that the ion conductivity retention rate of LTOC-5AlF_3 is better than that of LTOC (Fig. S42).

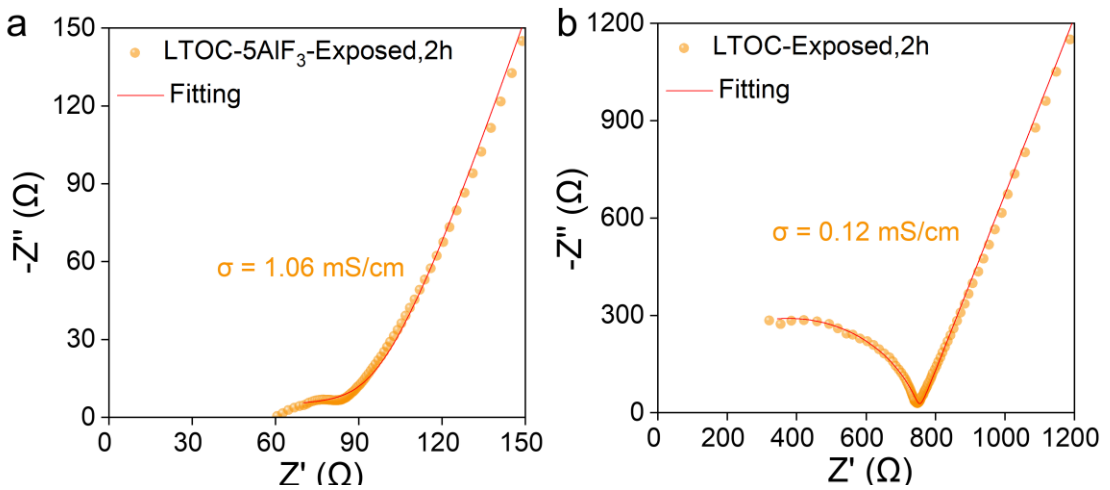

Fig. S42 | a The EIS of LTOC-5AlF_3 after exposure (dew point of -10 °C) (chsq 3.83×10^{-4}). **b** The EIS of LTOC after exposure (dew point of -10 °C) (chsq 1.55×10^{-4})

The reason why AlF_3 can improve the stability of solid-state electrolyte humidity is that it can simultaneously provide chemical and physical protection.

Chemical protection: It has been confirmed that AlF_3, as a functional module, partially interacts with the amorphous phase to form an amorphous structure in conjunction with LTOC. This interaction results in the formation of stronger Ta-F bonds compared to Ta-O or Ta-Cl bonds, which can mitigate reactivity with water to some extent (Fig. S43)[3, 4].

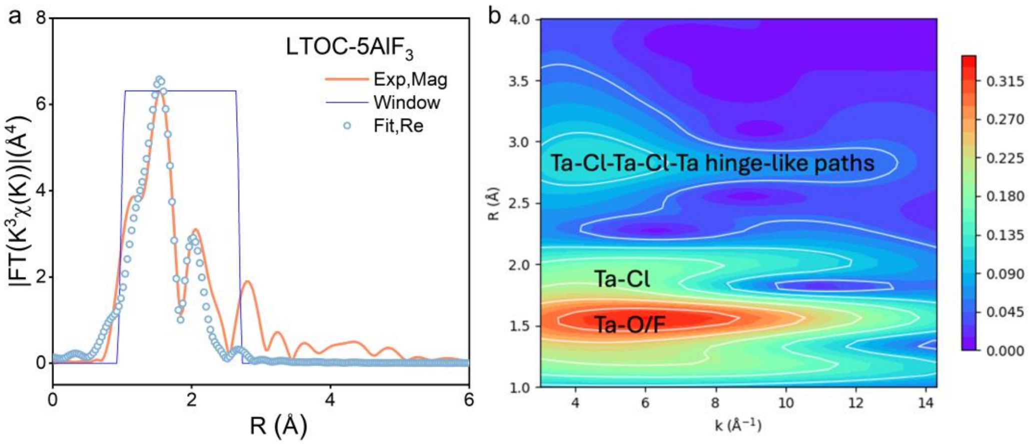

Fig. S43 | a Fourier transform of k^3-weighted Ta L_3-edge EXAFS curves of LTOC-5AlF_3, **b** WT-EXAFS of LTOC-5AlF_3 electrolyte at Ta L_3-edge. A k^3 weighting was used.

Physical protection: According to the chemical reaction equation, it can be inferred that $TaCl_5$ reacts spontaneously and violently with water, whereas AlF_3 does not react with water. Therefore, AlF_3 exhibits a greater tolerance to water compared to $TaCl_5$. Due to the high proportion of AlF_3 introduced, it cannot completely form an amorphous structure and will exist in the form of AlF_3. Coupled with the low density of AlF_3, the portions of AlF_3 exposed to the air surface effectively reduce physical contact between the internal amorphous phase and water in the air and reduce the available surface area for moisture to attack the surface of LTOC particles without completely breaking the conduction network. Given that AlF_3 itself possesses good air stability, it can enhance the air stability of the electrolyte.

Equations for the Reaction of $TaCl_5$ and AlF_3 with H_2O (Data from MaterialProject):

$$0.25TaCl_5+0.75H_2O \longrightarrow 0.25TaCl_3O+0.5H_3ClO \quad -0.016\ \text{eV/atom}$$

$$AlF_3 + H_2O \longrightarrow AlF_3 + H_2O \quad \text{no reaction}$$

Based on these two reasons, it can be determined that AlF_3 can improve the stability of solid-state electrolyte humidity. However, it is important to note that this strategy does not entirely resolve the issue of air stability in halide electrolytes; rather, it improves the air stability of the electrolyte to a certain extent.

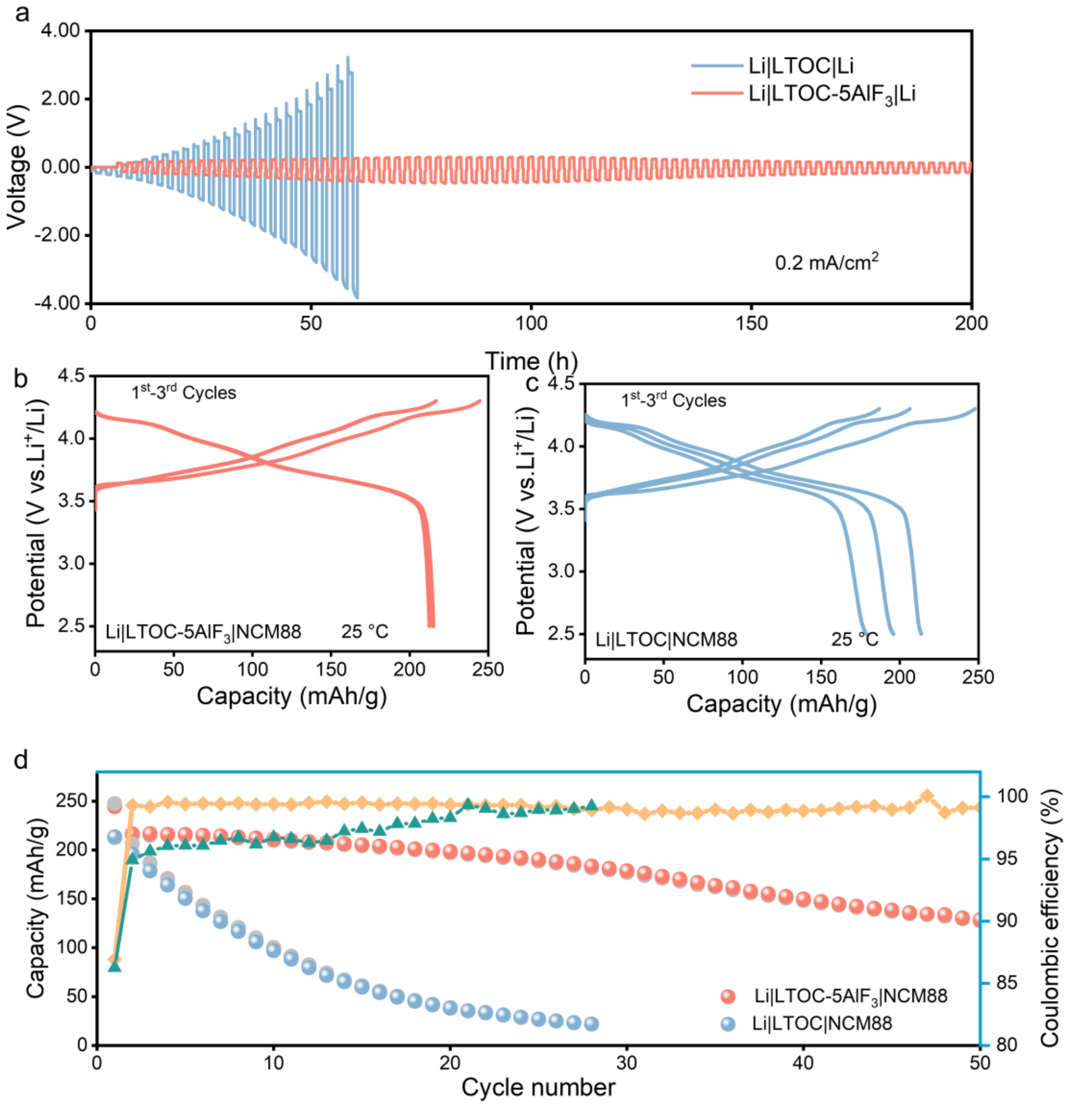

Fig. S44 | a The galvanostatic lithium stripping/plating performances of Li|LTOC|Li and Li|LTOC-5AlF_3|Li symmetric cells at 0.2 mA/cm^2 with a fixed areal capacity of 0.2 mAh/cm^2. **b** Discharge/charge curves of the Li|LTOC-5AlF_3|NCM88 ASSLMB at 25 °C. **c** Discharge/charge curves of the Li|LTOC|NCM88 ASSLMB at 25 °C. d Long-term cycling performance and coulombic efficiency at 20 mA/g of the Li|LTOC-5AlF_3|NCM88 and Li|LTOC|NCM88 ASSLMB at 25 °C

Halide solid electrolytes are thermodynamically unstable with lithium metal, leading to reactions between them. Consequently, an increase in overpotential is observed during the early Li plating/stripping of lithium symmetric cells. Literature reports indicate that electrolytes stable with lithium metal also exhibit this phenomenon of overpotential increase[5, 6, 7]. Currently, the primary approach to improving the stability of lithium metal in these systems is the use of in situ or ex situ interfacial layers. The

stability of lithium metal discussed in this work is comparable to that reported in the literature. However, it is not an intrinsic thermodynamic stability but rather a kinetic stability achieved through the formation of an interfacial layer after Li plating/stripping.

Although we have standardised the testing procedures for lithium symmetric cells according to the literature, our survey of published studies revealed certain limitations of this method. The ability of lithium symmetric cells to cycle stably and exhibit high critical current density reflects only one aspect of the interfacial stability between the electrolyte and lithium metal. Therefore, we assess the stability of LTOC-AlF_3 against lithium metal through a multi-faceted approach combining lithium symmetric cells and all-solid-state lithium metal batteries (Fig. S44). Our experiments show that AlF_3, as a functional module, can improve the interfacial instability between the LTOC electrolyte and lithium metal to some extent. This intriguing phenomenon can guide us in the formation of such materials to enhance their kinetic stability with metallic lithium. We believe that in the future, through the modulation and optimisation of functional modules, we can generate a more favourable kinetically stable interfacial layer, further improving the interfacial issues between halide electrolytes and lithium metal.

Table S1 Summary of RT ionic conductivities and activation energy of LTOC-xLa obtained by impedance spectra of the cold-pressed LTOC-xLa pellets. The data is based on the measurements for two or three repeated samples of each composition.

Samples	RT ionic conductivity (mS/cm)	Activation energy (eV)
LTOC	7.7	0.312
LTOC-0.2La	13.3	0.304
LTOC-0.4La	11.4	0.307
LTOC-0.6La	10.0	0.272
LTOC-0.8La	8.7	0.282
LTOC-1.0La	6.9	0.303

Table S2. Relaxation Times, T1, of LTOC sample derived from ^{7}Li MAS-NMR

Peak name	F2 (ppm)	T1 (s)	error
1	0.472	0.384	0.01415
2	-0.167	1.36	0.3042

Table S3. Relaxation Times, T1, of LTOC-0.2La sample derived from ^7Li MAS-NMR

Peak name	F2 (ppm)	T1 (s)	error
1	0.436	0.362	0.01645
2	-0.178	1.35	0.3180

Table S4. The Young's modulus of LTOC-xLa (x = 0, 0.2, 0.4, 0.6, 0.8, 1.0) SSEs

SSE	Density (g/cm^{-3})	P-wave velocity (m/s)	S-wave velocity (m/s)	Young's modulus (GPa)
LTOC	3.5287	2510.07	1157.89	12.914
LTOC-0.2La	3.4341	2885.24	1079.75	11.359
LTOC-0.4La	3.3794	2161.49	1104.76	10.915
LTOC-0.6La	3.3013	2210.52	1127.52	11.114
LTOC-0.8La	3.2078	2820.14	1187.88	12.603
LTOC-1.0La	3.0995	2684.21	1378.38	15.106

Table S5. Comparison of functionalized SSEs with Others.[1, 5, 7, 8, 9, 10, 11, 12, 13, 14, 15, 16, 17, 18, 19, 20, 21, 22]

SSE	Ionic conductivity	Temperature	Ref.
L_3YCl_6	0.51	room temperature	8
L_3ScCl_6	2.04	25 °C	7
$Li_{0.388}Ta_{0.238}La_{0.475}Cl_3$	3.02	30 °C	5
Li_2ZrCl_6	0.81	room temperature	10
$LiTaCl_6$	10.95	25 °C	11
$Li_7La_3Zr_2O_{12}$	1.7	25 °C	19
$Li_{6.4}La_3Zr_{1.4}Ta_{0.6}O_{12}$	1.01	25 °C	20
$Li_{1.3}Al_{0.3}Ti_{1.7}(PO_4)_3$	0.51	25 °C	21
$Li_{1.5}Al_{0.5}Ge_{1.5}(PO_4)_3$	0.5	room temperature	22
Li_3OCl	0.85	room temperature	18
$LiNbOCl_4$	10.7	25 °C	15
$LiTaOCl_4$	12.8	25 °C	15
ZrO_2-Li_2ZrCl_6	2.42	30 °C	1
$Li_{1.75}ZrCl_{4.75}O_{0.5}$	1.35	25 °C	16
xLi_2O-$TaCl_5$	6.6	25 °C	17
Li_2O-1.8$TaCl_5$-0.2$LaCl_3$	13.3	25 °C	This work
Li_2O-1.8$TaCl_5$-0.2$HfCl_4$	12.6	25 °C	This work
Li_2O-1.8$TaCl_5$-0.2YCl_3	11.4	25 °C	This work
Li_2O-1.8$TaCl_5$-0.2$ZrCl_4$	10.4	25 °C	This work
Li_2O-1.8$TaCl_5$-0.2AlF_3	12.74	25 °C	This work
Li_2O-1.8$TaCl_5$-0.2MgF_2	10.2	25 °C	This work
Li_2O-1.8$TaCl_5$-0.1Ti_2O_3	10.4	25 °C	This work

Supplementary references

1. Kwak H*, et al.* Boosting the interfacial superionic conduction of halide solid electrolytes for all-solid-state batteries. *Nature Communications* **14**, 2459 (2023).
2. Duan H*, et al.* Amorphous AlOCl Compounds Enabling Nanocrystalline LiCl with Abnormally High Ionic Conductivity. *Journal of the American Chemical Society* **146**, 29335-29343 (2024).
3. Chen X, Jia Z, Lv H, Wang C, Zhao N, Guo X. Improved stability against moisture and lithium metal by doping F into Li3InCl6. *Journal of Power Sources* **545**, 231939 (2022).
4. Wu M*, et al.* Fluorinated amorphous halides with improved ionic conduction and stability for all-solid-state sodium-ion batteries. *Nature Communications* **16**, 2808 (2025).
5. Yin Y-C*, et al.* A $LaCl_3$-based lithium superionic conductor compatible with lithium metal. *Nature* **616**, 77-83 (2023).
6. Yu T*, et al.* Superionic Fluorinated Halide Solid Electrolytes for Highly Stable Li-Metal in All-Solid-State Li Batteries. *Advanced Energy Materials* **11**, 2101915 (2021).
7. Liang J*, et al.* Site-Occupation-Tuned Superionic Li_xScCl_{3+x} Halide Solid Electrolytes for All-Solid-State Batteries. *Journal of the American Chemical Society* **142**, 7012-7022 (2020).
8. Asano T, Sakai A, Ouchi S, Sakaida M, Miyazaki A, Hasegawa S. Solid Halide Electrolytes with High Lithium-Ion Conductivity for Application in 4 V Class Bulk-Type All-Solid-State Batteries. *Adv Mater* **30**, e1803075 (2018).
9. Li X*, et al.* Water-Mediated Synthesis of a Superionic Halide Solid Electrolyte. *Angewandte Chemie International Edition* **58**, 16427-16432 (2019).
10. Wang K*, et al.* A cost-effective and humidity-tolerant chloride solid electrolyte for lithium batteries. *Nature Communications* **12**, 4410 (2021).
11. Ishiguro Y, Ueno K, Nishimura S, Iida G, Igarashib Y. TaCl5-glassified Ultrafast Lithium Ion-conductive Halide Electrolytes for High-performance All-solid-state Lithium Batteries. *Chemistry Letters* **52**, 237-241 (2023).
12. Yu S*, et al.* Elastic Properties of the Solid Electrolyte Li7La3Zr2O12 (LLZO). *Chemistry of Materials* **28**, 197-206 (2016).
13. Manthiram A, Yu X, Wang S. Lithium battery chemistries enabled by solid-state electrolytes. *Nature Reviews Materials* **2**, 16103 (2017).
14. Lü X*, et al.* Li-rich anti-perovskite Li3OCl films with enhanced ionic conductivity. *Chemical Communications* **50**, 11520-11522 (2014).
15. Tanaka Y, Ueno K, Mizuno K, Takeuchi K, Asano T, Sakai A. New Oxyhalide Solid Electrolytes with High Lithium Ionic Conductivity >10 mS cm^{-1} for All-Solid-State Batteries. *Angewandte Chemie International Edition* **62**, e202217581 (2023).
16. Hu L*, et al.* A cost-effective, ionically conductive and compressible oxychloride solid-state electrolyte for stable all-solid-state lithium-based batteries. *Nature Communications* **14**, 3807 (2023).
17. Zhang S*, et al.* A family of oxychloride amorphous solid electrolytes for long-cycling all-solid-state lithium batteries. *Nature Communications* **14**, 3780 (2023).
18. Zhao Y, Daemen LL. Superionic Conductivity in Lithium-Rich Anti-Perovskites. *Journal of the American Chemical Society* **134**, 15042-15047 (2012).
19. Nasir M, Park JY, Heo P, Choi KH, Park HJ. Li-La-Zr-O Garnets with High Li-Ion Conductivity and Air-Stability by Microstructure-Engineering. *Advanced Functional Materials* **33**, 2303397

(2023).

20. Seung Ho Kwon*, et al.* In-situ photo-polymerized elastomeric composite electrolytes containing $Li_{6.4}La_3Zr_{1.4}Ta_{0.6}O_{12}$ particles for stable operation in lithium metal batteries. *EcoMat* **6 12**, (2024).

21. Waetzig K*, et al.* Synthesis and sintering of $Li_{1.3}Al_{0.3}Ti_{1.7}(PO_4)_3$ (LATP) electrolyte for ceramics with improved Li^+ conductivity. *Journal of Alloys and Compounds* **818**, 153237 (2020).

22. Meesala Y*, et al.* All-Solid-State Li-Ion Battery Using $Li_{1.5}Al_{0.5}Ge_{1.5}(PO_4)_3$ As Electrolyte Without Polymer Interfacial Adhesion. *The Journal of Physical Chemistry C* **122**, 14383-14389 (2018).
