# Supplementary material for: Functional modules for enhanced amorphous composite halide solid electrolytes for low-temperature all-solid-state lithium batteries
Source: Nat Commun. 2026 May 27;17:6891. doi: 10.1038/s41467-026-71876-0 (PMC13388947; doi:10.1038/s41467-026-71876-0)
Supplement: Supplementary file 2 — Description of Additional Supplementary Files [file 41467_2026_71876_MOESM2_ESM.pdf]

Description of Additional Supplementary Files

Supplementary Data 1. The source files of the electronic structure calculation.
