# Supplementary material for: Functional modules for enhanced amorphous composite halide solid electrolytes for low-temperature all-solid-state lithium batteries
Source: Nat Commun. 2026 May 27;17:6891. doi: 10.1038/s41467-026-71876-0 (PMC13388947; doi:10.1038/s41467-026-71876-0)
Supplement: Supplementary file 4 — Transparent Peer Review file [file 41467_2026_71876_MOESM4_ESM.pdf]

nature portfolio

Peer Review File

Functional Modules for Enhanced Amorphous Composite Halide Solid Electrolytes for Low-temperature All-solid-state Lithium Batterie

Corresponding Author: Professor Xueliang Sun

Parts of this Peer Review File have been redacted as indicated to remove third-party material.

This file contains all reviewer reports in order by version, followed by all author rebuttals in order by version.

Version 0:

Reviewer comments:

Reviewer #1

(Remarks to the Author)
The author reported fillers effects in amorphous Li2O-1.8TaCl5 solid electrolyte. Different fillers can improve specific properties of solid electrolytes, for example the ionic conductivity, interfacial stability. Although a lot of data are given, the fundamental reason of how the fillers work is rather unclear.
1.The author reported the LaCl3 as the filler to improve the ionic conductivity, at x = 0.2, the materials is still completely amorphous which indicates the LaCl3 is probably incorporated into LTOC matric, which lead to the highest ionic conductivity rather than the filler effects. With higher LaCl3 content, excess LaCl3 segregates out, thus can be seen from XRD and conductivity drops gradually.
2.The specific fillers effects in driving high ionic conductivity are unclear, is it leading to higher interfacial conduction? The increasing Li 2 in NMR is due to more interfacial Li at the LTOC-LaCl3 interface? Furthermore, with some of the fillers, the Li NMR peaks narrow which correspond to fast Li motion then high ionic conductivity as expected. But some of the materials has rather same peak width, but the ionic conductivity is higher?
3.The figure 3 contains cells data with too low loading which is unacceptable, the supporting information high loading cells data should be put into main text instead. And highlights fast cycling at 10C and low temperature performance with 5 mg/cm2 loading is not valid.
4.All the characterizations XPS, TOF-SIMS and SEM are done in the low loading cell cycled at 10C, with such high current, thus high overpotential, the NCM is not really charged to 4.3V and exhibits low discharge capacity. So characterization should be done on cells cycled at lower rate to have full capacity of NMC 200mAh/g.
5.The claim about AlF3 filler drives the Li metal stability doesn't agree with the data, the symmetrical cell obviously shows significant overpotential increases, thus interfacial reaction. Thus the CCD test is not right.
6.Furthermore, does AlF3 just work as filler, is any Florine exchange with Cl? And why with high AlF3 content, the moisture stability is better is not explained. And it is not improved much, since it is test in -30C dew point dry room.

Reviewer #2

(Remarks to the Author)
This paper is reporting the role of adding inorganic filler to Li2O-1.8TaCl5 (LTOC). Although the concept was previously reported (Nat. Commun. 14, 2459, 2023) and engineering the decomposition products of solid electrolytes has been popular, the high ionic conductivity of LTOC-0.2La (13.3 mS/cm) and LTOC-5AlF3 (7 mS/cm, despite of large molar fraction of AlF3) is worthy to report in this journal. However, the microstructure and ionic conduction mechanism of the materials are unclear, which does not satisfy high scientific standard of Nat. Commun. The clear understanding on these materials should be provided for the publication.
The direct evidence of the presence of intact inorganic fillers (LaCl3, AlF3 and etc.) should be provide by either spectroscopy, diffractometer and/or microscopy. For example, the presence of LaCl3 and AlF3 has not been proved.
What is the role of inorganic fillers for increasing ionic conductivity of LTOC-0.2La?
The local environment of metal ions of the inorganic fillers (La, Al) should be verified. Does the inorganic fillers exist in intact

or it form a part of amorphous matrix? XRD patterns (Fig.1d (x=0.2) and Fig. S18), and EDS mapping (Fig. S5,S6) seems to suggest that those are homogeneous amorphous materials rather than the mixtures of LTOC and inorganic fillers.
The mechanism of high ionic conductivity of LTOC-0.2La and LTOC-5AlF3 should be discussed. Is there any possible doping effect of La3+ and Al3+?
The 7Li NMR spectra of LTOC shown in Fig. 1g looks different form what shown in the reference [27]. Could you provide an explanation?
In the sentence "In the 1D 7Li NMR spectra of LTOC…", the reference [27] is more suitable because the reference [20] is reporting Li NMR spectra of oxygen-free Li0.5TaCl5.5.
In the sentence "The presence of a higher number of… La sites…", Is 'La' a typo?
In the sentence "The presence of a higher number of… La sites…", presumably, increasing less-electronegative Cl- (vs. O2-) may result in a stronger shielding effect. For example, the 7Li NMR spectrum of LTOC-5AlF3 (Fig. S23) shows a significant de-shielding compared to that of LTOC, possibly due to the presence of highly electro-negative F-?
Overall, proving LTOC-5AlF3 and LTOC-0.2La are not a common amorphous glassy material but a (nano-)mixture utilizing inorganic fillers and ascertaining how ionic conductivity is increased by adding inactive inorganic filler are crucial.

Reviewer #3

(Remarks to the Author)
Wu et al. present a very interesting study on incorporating functional modules ("fillers") into well-known amorphous SSE compositions to tune their properties and widen their applicability in battery systems. They present compelling cycling performance and extensive characterization to support their materials engineering efforts, and I believe this work will spark wide interest in terms of how it enlarges the design-space for SSE. However, it is also heavily phenomenological and I have highlighted a few areas where I think more scientific insight would be helpful/of interest to readers not solely focused on battery performance.

One primary "big-picture" question: what is the interplay between filler loading and particle size, especially for cases when the functional modules become part of a homogenous network in the composite particles? Could 'zoning' of the functional modules towards the exterior of, in particular, small/nanosized SSE particles impact the extent of interfacial contacts for conduction into the electrode? Correspondingly, how do the functional modules affect particle morphology? There is a series of SEM images for one LTLOC composition (Fig. S4 – also, please label which one this is in the caption), but it would be helpful to also have a series spanning the full range of $LaCl_3$ incorporation levels. Especially as further nanostructuring in the composite cathode to improve conduction percolation is suggested as a future direction, and the ^{7}Li NMR demonstrates LiCl exsolvation, this seems worthy of addressing.

The following are specific points that I believe the authors should address:

Line 65: "doped halide SSEs with superior characteristics" – a bit more specificity would be helpful, in the sense of which "superior characteristics" are intended to be highlighted (especially with four separate references cited towards this point).

Line 89-101: This section of the final intro paragraph largely repeats similar content as the abstract; I think it would be improved if the authors instead offered additional details at this stage to rationalize why one would pick, e.g., an oxide versus a chloride functional module, and what drives the selection of one over another in trying to regulate a given property of the SSE (essentially, previewing the results a bit more specifically, more like the beginning of the paragraph where LaCl3 is used as an example, in order to reduce the repetition a bit).

Line 143: The site labeled as Li2 in the ^{7}Li MAS-NMR is most certainly exsolvated LiCl, which is commonly detected in halide SSE by ^{7}Li NMR. While the shift of the main amorphous peak with incorporation of $LaCl_3$ is most likely the result of the polarizability effect that the authors identify, it does also suggest that the incorporation of the functional module is simultaneously creating additional pockets of LiCl, as evidenced by the growth in the intensity of this site (either too small to be diffractable, or too poorly ordered). While it is possible that these sites are part of the conduction network and do contribute chemical exchange that is detected by the 2D EXSY, the cross-peaks there could also be due to spin diffusion (as the EXSY experiment is functionally equivalent to NOESY). The primary way to distinguish these cases is to perform the EXSY at a series of temperatures; spin diffusion is relatively insensitive to increasing temperature, whereas chemical exchange will show strong thermal activation (evidenced by increased cross peak volume at the same mixing time), especially as these are fast ion conductors. Further, there is an added dividend: the activation energy associated with the increased exchange rate will provide a complementary energy scale that is comparatively more local than the activation energy derived from macroscopic conductivity measurements. Also, it would be helpful if the authors marked the mixing times on Figures 1h,i (or included them in the caption).

An additional point with the NMR results in Figure 1: the methods section states that the recycle delays are 0.5 s and 2 s (it's also unclear why there are two used?). At 600 MHz, this can be quite short relative to typical ^{7}Li relaxation times, especially for the LiCl component marked as Li2. Are the authors confident that there is no saturation in these measurements, which is affecting the relative proportions of Li1 and Li2? Also, it doesn't appear that the MAS rate is stated anywhere in the main text or the methods section; this is key for reproducibility.

Line 223: what is 'super-magnification' referring to in this context? Phrasing here is unclear.

Line 293: GITT is used to evaluate the diffusion coefficient, and support the notion that there is a lower transmission barrier at the SSE/electrode interface on account of the higher diffusion coefficient. It's not clear what this is higher relative to. A control experiment with un-modulated LTOC would be necessary here to contextualize this statement (the poorer interfacial stability of LTOC at high rate relative to LTLOC would presumably be negligible with perturbative GITT pulses). Also, as this is recording the combined response of the diffusion in both the cathode and the SSE, it's difficult to make any direct inference about the SSE contribution to the performance, especially absent a control.

Figure 4: panels b,c and e,f are essentially presenting the same information, and the figure is a bit cramped overall. Perhaps choosing one style for the DRT as a function of charge/discharge process and having larger panels overall would improve readability?

Line 375: "When $ZrCl_4$ is introduced, only one peak is observed" I infer from later context (and units) that this is the NMR again, but the preceding discussion involved both XPS and TOF-SIMS (which also discuss peaks), so it would be helpful for readability to directly point this out in the text.

Line 377: "Lithium in isolation or in a less complex environment exhibits relatively weak signals." Not sure what this is intended to mean; indeed, in a 'less complex' environment (say, a more symmetrical one like LiCl…) ^{7}Li will often have a _stronger_ signal because there is less homogenous broadening and the peak is sharper, all things being equal with populations (indeed, this is part of why the Li2 – LiCl – site doesn't shift much with addition of functional modules, because the local coordination is already highly symmetrical). I think the primary conclusion of these Fig. S21 results is, as the authors point out, that the addition of the functional modules only induces small changes in the material susceptibility (the small shifts) and does not significantly disrupt the amorphous conduction network of the LTOC, so this paragraph should be revised to just reflect this point.

Line 417: While the contrast between LTOC and LTOC-5AlF_3 is clear from the performance of the respective cells after humidity exposure is clear, what is the scientific hypothesis for the role of AlF_3 here? Is it basically just reducing the available surface area for moisture to attack the surface of LTOC particles without completing breaking the conduction network, in contrast to a coating? Mechanistically, this section in particular feels a bit unclear, and largely phenomenological.

As a general point: I can't help but think that referring to the functional modules as "filler" does a slight disservice to their role in expanding the capabilities of these materials. Perhaps a different term might be more effective? Or even just sticking with "functional modules" all the way through, rather than flipping back and forth between both terms?

Version 1:

Reviewer comments:

Reviewer #1

(Remarks to the Author)
The author tied to answer some of the reviewer's questions, but some of them still remain unclear.
1.The authors mainly claim their superior electrochemical performance, though the cell performance is not as good as previous reported (Chem. Lett. 2023, 52, 237). claiming best performance is not supported with their data. Furthermore, all the cell loading are still only around 10mg/cm2, way lower than practical requirement. Within the SI, some of the cells with ~5mg/cm2 loading, those cell data should be removed. And Figure 2 and S17 a-c are same set-up, but the low loading cell show worse rate performance than the high loading cell which make no sense and need to explain whether labeled wrong or why.
2.Figure 3f and S18 with y-axis starting from -50 is wrong.
3.With the material LTOC-0.2La, all the data says LaCl3 just dope into the materials, as the TEM and XRD show it is complete amorphous and all element evenly distributed, so it is just doping rather so called functional modules.
4.The question about LTOC-5AlF3 show better moisture stability is somewhat answered partially. Replacing some Cl bonded to Ta with F is clear, with such mechanism AlF3-xClx should be formed too, however will TaCl4-xFx and AlF3-xClx exhibit better moisture tolerance. Or possible just mainly because much excess AlF3 on the surface to act as physical barrier to water.

Reviewer #2

(Remarks to the Author)
The revised manuscript resolves the most of the raised concerns and good to publish in this journal.

Reviewer #3

(Remarks to the Author)
Overall, the authors have done a very reasonable job of addressing the questions posed in the original review. The

additional explanations provided are helpful in understanding the role of the functional modules, and the new calculations supporting the MSD increase in the presence of the functional modules greatly buttress the previous results. The flow and readability of the manuscript has also been improved, and as before, the main findings are compelling and likely to be of broad interest in the community.

While I do think that the VT-NMR results would be an asset and increase the rigour of the original EXSY experiments, I also recognize that it is challenging to schedule and conduct such experiments within the short turn-around time of a review response, and the new calculations do work well in demonstrating the key result that was being inferred from the original, single-temperature EXSY experiments. One point I would still make with the NMR results regards the potential saturation of the 7Li signals (particularly the LiCl component): a citation (#3) is given in the reviewer response to a different manuscript as support for the short recycle delay, but in that study, 5 s was utilized (i.e. an order of magnitude longer, and potentially still too short). Further, it does not follow that a similar error is not being propagated through the literature just because another example exists. The response also states "This selection of time is the result of multiple verifications by the testers in conjunction with the literature to ensure the accuracy and authenticity of the data." The nature of the 'multiple verifications' are not stated (there is really only one certain method: a formal T1 measurement on the sample). However, since the peak area quantifications are relative, not absolute, and (more importantly) primarily support qualitative arguments about the composition, I don't think this can be considered a fatal flaw (especially taking into account that the authors mention that they cannot easily conduct additional NMR experiments with their main facilities).

Correspondingly, I have no further criticisms/comments which have not been adequately addressed in the revisions/response.

Version 2:

Reviewer comments:

Reviewer #1

(Remarks to the Author)
No further questions.

Reviewer #3

(Remarks to the Author)
I appreciate the due-diligence of the authors in supplementing the original experiments with T1 measurements to verify their parameters. Given that the combined recycle delay and acquisition durations (+ mixing time as well, for the EXSY experiments) are on the order of (2-3)*T1 for the T1 values of the Peak 2 signals (LiCl; the longer of the T1 times), then it is confirmed that saturation is not a significant concern and the relative peak quantifications can be taken as accurate to within a few percent. This would not have been a priori clear for a high-symmetry site like LiCl - especially at 14.1 T - so I'm glad that the authors took the time to make the extra measurements and check.

Recognizing as before the significant efforts to address the comments from the previous round of review, I have no further criticisms/comments at this stage.

Open Access This Peer Review File is licensed under a Creative Commons Attribution 4.0 International License, which permits use, sharing, adaptation, distribution and reproduction in any medium or format, as long as you give appropriate credit to the original author(s) and the source, provide a link to the Creative Commons license, and indicate if changes were made.
In cases where reviewers are anonymous, credit should be given to 'Anonymous Referee' and the source.
The images or other third party material in this Peer Review File are included in the article’s Creative Commons license, unless indicated otherwise in a credit line to the material. If material is not included in the article’s Creative Commons license and your intended use is not permitted by statutory regulation or exceeds the permitted use, you will need to obtain permission directly from the copyright holder.
To view a copy of this license, visit https://creativecommons.org/licenses/by/4.0/

Response to the Comments of Reviewers on " Building Blocks of Battery Innovation: Modular Design of Amorphous Electrolytes"(Research article, #NCOMMS-25-15945A)

We highly appreciate the reviewers' recommendation to publish this paper in **Nature Communications** and are also very grateful to them for providing constructive comments and insightful suggestions for further improving the quality of the manuscript. We have revised the manuscript with great effort. We carefully addressed the concerns and corrected any errors. The specific responses and revisions are listed below. We believe that the paper is now stronger and thank the referees for their constructive comments very much.

The most significant improvements in the revised version of the manuscript can be summarised as follows:

1. We fundamentally analyzed the role of functional modules and investigated the potential mechanisms by which functional modules ($LaCl_3$, AlF_3) improve the performance of solid-state electrolytes through multi-characterization techniques such as XRD, NMR, and TEM, combined with first-principles calculations, as well as the impact of functional modules on the chemical and structural properties of the solid-state electrolytes.

2. Modifications have been made regarding the solid-state NMR-related issues to enhance the rigour of the argumentation, and a more detailed explanation of the solid-state NMR data has been provided, referencing relevant literature.

3. We explored the influence of the amount of functionalized modules on the physical properties of solid-state electrolyte materials, such as morphology, particle size, and Young's modulus.

4. A detailed explanation of the mechanism by which AlF_3 enhances air stability as a functional module has been provided, further deepening the understanding of how

functional modules regulate electrolyte performance.

5. The data on higher loading electrochemical performance and post-cycling XPS, TOF-SIMS, and SEM data at low rates have been added.

6. Other concerns have also been addressed.

Reviewer 1#

The author reported fillers effects in amorphous Li_2O-1.8$TaCl_5$ solid electrolyte. Different fillers can improve specific properties of solid electrolytes, for example, the ionic conductivity, interfacial stability. Although a lot of data is given, the fundamental reason for how the fillers work is rather unclear.

1#-1. The author reported $LaCl_3$ as the filler to improve the ionic conductivity. at x = 0.2, the material is still completely amorphous, which indicates that $LaCl_3$ is probably incorporated into the LTOC matrix, which leads to the highest ionic conductivity rather than the filler effects. With higher $LaCl_3$ content, excess $LaCl_3$ segregates out, and thus, can be seen from XRD and conductivity drops gradually.

1#-2-A. The specific filler effects in driving high ionic conductivity are unclear; is it leading to higher interfacial conduction?

Response: Thank you for your comments. In this response, we address Reviewer Comments 1 and 2A together.

1. We have replaced all instances of 'fillers' with 'functional modules' to ensure consistency and rigour throughout the article. We found that the term "filler" was improperly used and could lead to ambiguity. Therefore, we have replaced "filler" with "functional module" in the text. The purpose of this work is to introduce a component that can maintain ionic transport (whether it enhances or minimally reduces ionic conductivity) while also adjusting other properties of the electrolyte. We believe that the term "functional module" is more appropriate, as it is based on a design perspective. This term aims to emphasise the introduction of substances with regulatory functions, similar to functional additives in electrolyte liquid design. Thus, the functional module and the amorphous part are not simply a physical mixture but interact with each other. The electrolyte can be entirely amorphous (LTOC-0.2La) or may contain some crystalline phases (for example, LOTC-xLa, where x = 0.4-1.0). In both cases, we refer to $LaCl_3$ as a "functional module."

2. We conducted a further in-depth analysis of the mechanisms by which different functional modules enhance the performance of electrolytes. This study provides a comprehensive understanding of how functional modules improve ionic conductivity and interfacial stability, and elucidates the role of functional modules in this context. We clarified the fundamental reasons behind the ability of functional modules to regulate performance.

2.1 The uniqueness of amorphous electrolyte materials. Unlike crystalline materials with rigid structures, amorphous oxyhalide SSEs benefit from long-range disorderliness[1]. This eliminates dependence on a fixed structure for ion transmission, leading to a more flexible and efficient path for ion movement. Therefore, we proposed regulating the properties of amorphous SSEs through a functional module design. The designed SSEs comprise two components: the ion transport part (amorphous LTOC) and the functional module part. This unique structure ensures efficient ion transmission and allows for artificial control using functional modules based on specific requirements. Notably, the incorporation of the functional module exerts minimal influence on the intrinsic conduction mechanism of the amorphous matrix; it can serve a great role in functional design.

2.2. Progressiveness and flexibility of functional modules. The functional modules and amorphous parts do not merely exist as a physical mixture; instead, they interact with each other. Similar to additives in liquid electrolytes, these modules are integrated into the primary structure of the liquid electrolyte. The functional modules can facilitate the formation of either a homogeneous amorphous phase or a composite structure that includes an amorphous matrix alongside a secondary phase, a characteristic frequently observed in polymer electrolytes. The primary advantages of this design approach are its ability to retain benefits while compensating for limitations, collectively enhancing overall performance. Furthermore, this method is not constrained by the proportions and types of materials used.

2.3 The fundamental reason why $LaCl_3$ enhances ion conductivity as a functional module. We carried out a comprehensive investigation of the interaction

between $LaCl_3$, serving as a functional module, and the amorphous component of LTOC by combining XRD, TEM, NMR, and first-principles calculations. The results reveal that this interaction not only entails doping effects but also enhances interfacial ionic conduction.

2.3.1 Doping effect

Characterisation results confirm that $LaCl_3$ doping effectively enhances the ionic conductivity. The doping effect of $LaCl_3$, particularly the introduction of La^{3+}, plays a crucial role in enhancing ionic conductivity. However, excessive La^{3+} introduction may lead to $LaCl_3$ precipitation, which could reduce ionic conductivity. Theoretical calculations reveal that the introduction of La^{3+} alters the local environment of LTOC. The doping effect of cations leads to a differentiation in the local lithium-ion migration energy barrier of the electrolyte. The vibrational frequency of lithium ions in channels adjacent to La atoms is markedly higher than that of ions located further away, suggesting a reduced migration energy barrier in these regions, thereby facilitating ion transport (**Fig. R1**). Nevertheless, even with the introduction of $0.8LaCl_3$ (LTOC-0.8La), which exhibits an ionic conductivity of 8.7 mS/cm, this value remains higher than that of the ionic conductivity of amorphous LTOC (7 mS/cm), indicating the persistence of the doping effect of $LaCl_3$. Thus, ion conductivity is closely related to doping.

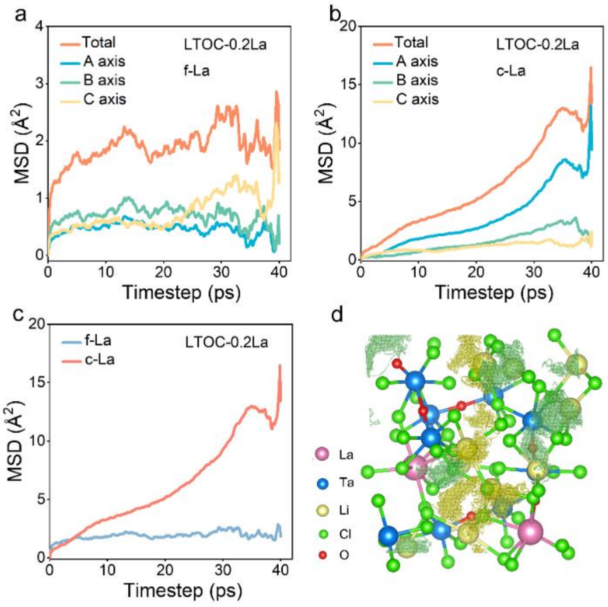

Fig. R1 | ab initio molecular dynamics (AIMD) simulations of LTOC-0.2La. **a-c** Mean Squared Displacement of Li^+ in LTOC-0.2La. **d** The structure and potential isosurface of LTOC-0.2La are visualised using VESTA. (f-La represents being far away from La, c-La represents being close to La).

2.3.2 Interface enhancement mechanism

The findings indicate that, beyond the doping-induced improvement in ionic conductivity, the interfacial enhancement arising from the incorporation of functional modules is also a critical factor that warrants attention. $LaCl_3$ interacts with the amorphous phase of LTOC, facilitating lithium-ion transport at the interface. Theoretical calculations further suggest that similar interfacial enhancement effects have been observed in systems such as AlOCl-nanoLiCl and ZrO_2-Li_2ZrCl_6[2, 3]. By analysing the mean square displacement, it was found that the introduction of $LaCl_3$ significantly promotes ion transport at the interface. This highlights the importance of rapid conduction at the interface in designing functionalized modules for electrolytes. This greatly facilitates the connectivity of lithium-ion transport pathways between the bulk phase and the interface, forming a more complete, closed, and multidimensional lithium-ion transport channel, which effectively reduces the migration barrier of lithium ions and thus enhances lithium-ion conductivity (**Fig. R2**).

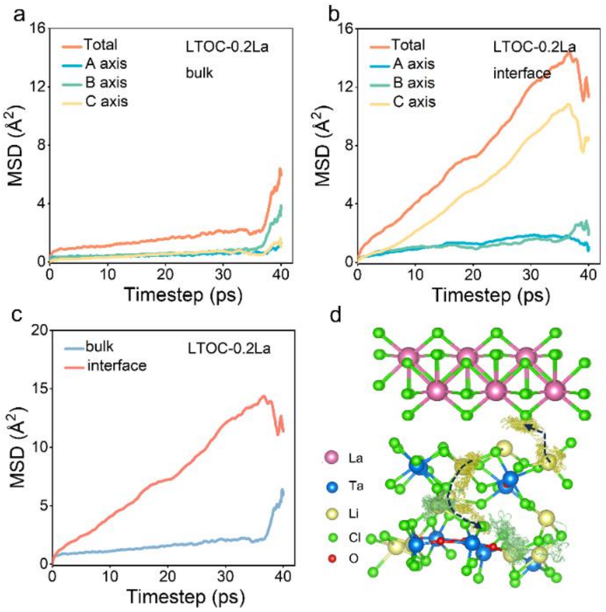

Fig. R2 | ab initio molecular dynamics (AIMD) simulations of LTOC-0.2La. **a-c** Mean Squared Displacement of Li^+ in LTOC-0.2La. **d** The structure and potential isosurface of LTOC-0.2La are visualised using VESTA.

In this work, the introduction of $LaCl_3$ serves merely as an example to illustrate the feasibility of the functional module design concept. The mechanism by which $LaCl_3$ enhances ionic conductivity is specific to the LTOC-x$LaCl_3$ system, and the effects of other functional modules on ionic transport must be evaluated individually. Overall, the incorporation of $LaCl_3$ effectively improves ionic conductivity through the synergistic effects of promoting lithium-ion transport at the interface and the doping effect of La^{3+}.

2.4 The fundamental reason why $LaCl_3$ enhances interfacial stability as a functional module. $LaCl_3$, as a functional module, is advantageous for concurrently enhancing the migration kinetics and thermodynamic stability of interface ions, which in turn improves interface stability.

2.4.1 Kinetics of interface

Interface analysis using in-situ EIS, DRT, XPS, and ToF-SIMS reveals the evolution and stability mechanism of the interface during prolonged cycling. The diffusion coefficient during charge and discharge was evaluated using GITT, as shown

in **Fig. R3**, which presents the charge and discharge GITT curves. Compared to NCM88/LTOC ASSB, NCM88/LTLOC ASSB has higher ion diffusion. A higher lithium-ion diffusion coefficient suggests a lower transmission barrier at the interface between the LTLOC and the cathode.

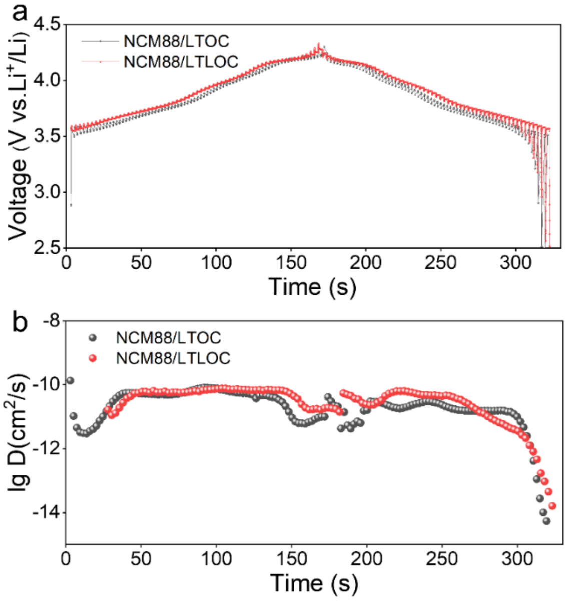

Fig. R3 | a The GITT curves of NCM88/LTOC and NCM88/LTLOC ASSBs. **b** The Li^+ diffusion coefficient of NCM88/LTOC and NCM88/LTLOC ASSBs

This study employed EIS and DRT to intensively uncover NCM88/LTLOC ASSB dynamic processes. Following numerous cycles, impedances at different frequencies were consistently low (**Fig. R4f, 4g**). Despite fluctuations in impedance values at mid and low frequencies, they remained small throughout, suggesting excellent diffusion kinetics at the interface of the cathode material and SSE during cycling.

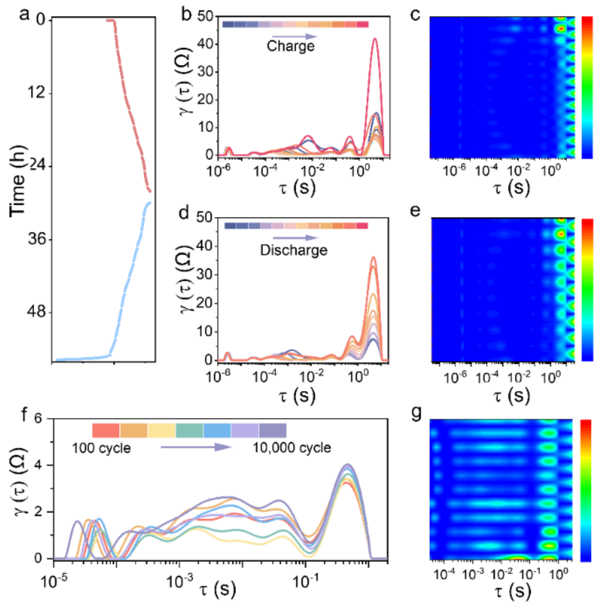

Fig. R4 | Dynamic analysis of NCM88/LTLOC by electrochemical impedance spectroscopy (EIS) and distribution of relaxation time (DRT). **a** The in-situ charge-discharge curve and the discontinuous point align with the EIS testing point. **b** DRT is calculated from the EIS measurements in charge. **d** DRT is calculated from EIS measurements during discharge. **f** DRT calculated from EIS measurements at different cycles. **c**, **e**, and **g** 2D intensity colour map of the charge-dependent DRT curves in (b), (d), and (f), respectively.

2.4.2 Thermodynamics of interface

The in-situ generation of the oxide phase containing La_2O_3 and the fast ionic conductor $LiLaO_2$ at the interface ensures high stability and excellent diffusion kinetics. This process significantly contributes to the enhanced performance of ASSBs, which exhibit remarkable uniformity and compactness (**Fig. R5-R7**). Furthermore, $LiLaO_2$ acts as a lithium-ion conductor, facilitating rapid lithium-ion diffusion at the interface.

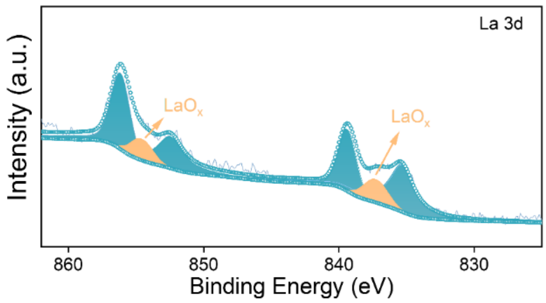

Fig. R5 | XPS spectra of La 3d after 200 cycles at a rate of 0.2 C (discharge specific capacity of approximately 200 mAh/g).

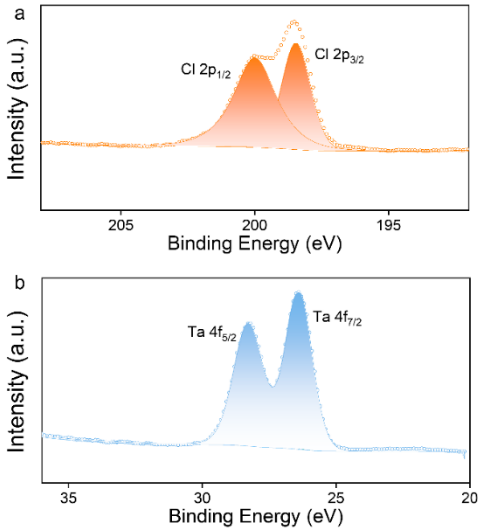

Fig. R6 | a XPS spectrum of Cl after 200 cycles at a rate of 0.2 C. **b** XPS spectrum of Ta after 200 cycles at a rate of 0.2 C (discharge specific capacity of approximately 200 mAh/g).

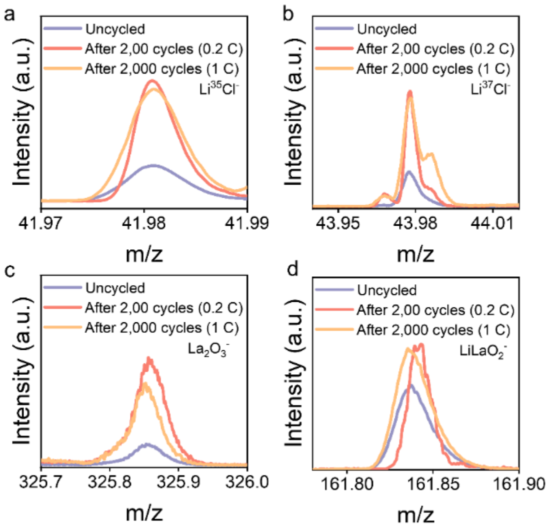

Fig. R7 | ToF-SIMS surface analysis results of **a** $Li^{35}Cl^-$, **b** $Li^{37}Cl^-$, **c** $La_2O_3^-$, **d**

$LiLaO_2^-$ at a rate of 0.2 C (discharge specific capacity of approximately 200 mAh/g).

1#-2-B. The increasing Li2 in NMR is due to more interfacial Li at the LTOC-$LaCl_3$ interface? Furthermore, with some of the fillers, the Li NMR peaks narrow, which corresponds to fast Li motion, then high ionic conductivity, as expected. But some of the materials have rather the same peak width, but the ionic conductivity is higher?

Response: Thank you for your comments.

We have found that the Li2 in NMR is Li structure/chemical coordination environment similar to LiCl through the literature review. The increase in peak intensity at the Li2 in NMR position may be due to the precipitation of more Li structure/chemical coordination environment similar to LiCl[3, 4].

Figure Redacted

Fig. R8 | a-c 1D ^{7}Li MAS NMR spectra and deconvolution analysis results corresponding to A-LTC·LiCl (a), A-LTC·3LiCl (b), and A-LTC· 6LiCl (c)[4]. (***Journal of the American Chemical Society* 2023, *145* (50), 27774-27787. DOI: 10.1021/jacs.3c10602**)

Figure Redacted

Fig. R9 | a 1D ^{7}Li MAS NMR of AlOCl-nanoLiCl and LiCl. b 2D EXSY NMR of

AlOCl-nanoLiCl recorded with a mixing time of 0.5 s^3. (***Journal of the American Chemical Society*** **2024,** ***146*** **(43), 29335-29343. DOI: 10.1021/jacs.4c06498**)

3. In the same material, a narrowing of the Li NMR peak corresponds to rapid lithium-ion movement. However, the introduction of functionalized modules (such as YCl_3, $HfCl_4$, $ZrCl_4$, AlF_3, MgF_2, Ti_2O_3, etc.) results in a different local lithium environment within the electrolyte compared to LTOC, even though both are amorphous. This is due to the interaction between the functional modules and the amorphous portion (similar to the interaction with $LaCl_3$ as a functional module). Therefore, the local lithium environment in the electrolyte is no longer the same as that of LTOC. Since the two materials have different structures, it is not feasible to solely determine the level of lithium-ion conductivity based on the width of the Li NMR peak. At this point, the ionic conductivity may vary due to different doping effects and interfacial enhancement effects caused by different functional modules. Thus, even if the Li NMR peak widths are the same in different materials, the structural differences prevent a direct correlation with lithium-ion conductivity.

1#-3. The figure 3 contains cells data with too low loading which is unacceptable, the supporting information high loading cells data should be put into main text instead. And highlights fast cycling at 10 C and low temperature performance with 5 mg/cm^2 loading is not valid.

Response: Thank you for your valuable advice. We have enhanced the electrochemical data by increasing the loading (The loading was increased from 5 mg/cm^2 to 10-11.6 mg/cm^2) and have updated Figures 2a-2f and 3a-3f accordingly. Additionally, we have incorporated new content and revised both the manuscript and the supporting information, with all changes highlighted in yellow.

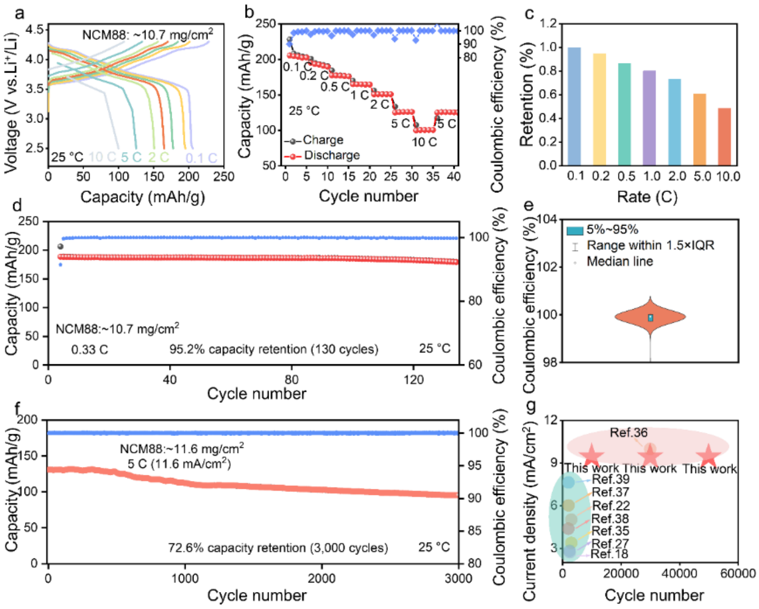

Fig. R10 | Electrochemical performance of NCM88/LTLOC/LPSC/Li-In (NCM88/LTLOC) at 25 °C. a Discharge/charge curves and **b** rate performance of the NCM88/LTLOC ASSB at different rates (0.1, 0.2, 0.5, 1, 2, 5, and 10 C). **c** The average discharge capacity retention rate. **d** Long-term cycling performance and coulombic efficiency at 0.33 C. **e** The coulombic efficiency distribution within 130 cycles. **f** Long-term cycling performance and coulombic efficiency at 5 C (3,000 cycles). **g** Comparison of cycling number and current density of ASSBs with different solid-state electrolytes.

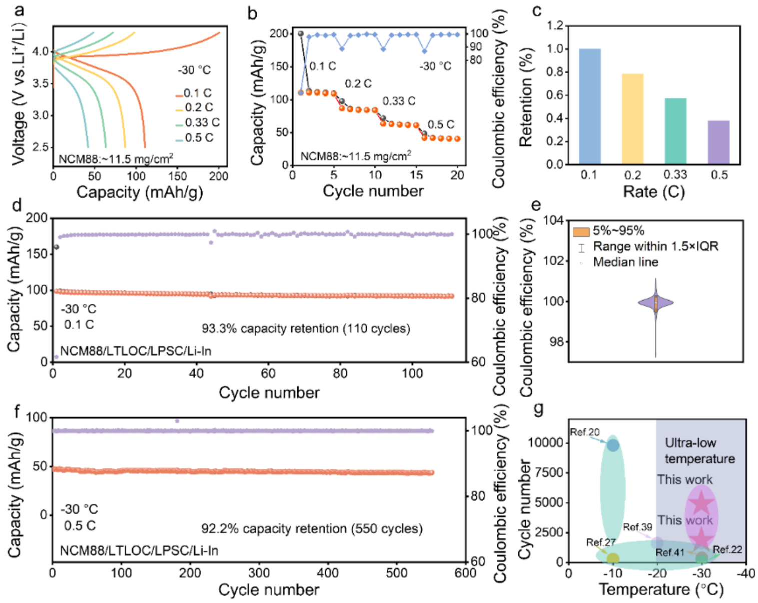

Fig. R11 | Electrochemical performance of NCM88/LTLOC/LPSC/Li-In

(NCM88/LTLOC) at -30 °C. **a** Discharge/charge curves and **b** rate performance of the NCM88/LTLOC ASSB at different rates (0.1, 0.2, 0.33, and 0.5 C). **c** The average discharge capacity retention rate. **d** Long-term cycling performance and coulombic efficiency at 0.1 C. **e** The coulombic efficiency distribution within 110 cycles. **f** Long-term cycling performance and coulombic efficiency at 0.5 C (550 cycles). **g** Comparison of cycling number and operating temperature of ASSBs with different solid-state electrolytes.

1#-4. All the characterisations XPS, TOF-SIMS and SEM are done in the low loading cell cycled at 10C, with such high current, thus high overpotential, the NCM is not really charged to 4.3 V and exhibits low discharge capacity. So characterization should be done on cells cycled at lower rate to have full capacity of NMC 200 mAh/g.

Response: Thanks for the advice. Cycling at high current (i.e., at a high overpotential and lower discharge capacity) may not accurately capture the true reactions occurring during the cycling process.

We have supplemented the XPS and TOF-SIMS, and SEM data of the all-solid-state battery after 200 cycles at a rate of 0.2 C (discharge specific capacity of approximately 200 mAh/g). We have added the following content and revised the manuscript and supporting information, and marked them yellow.

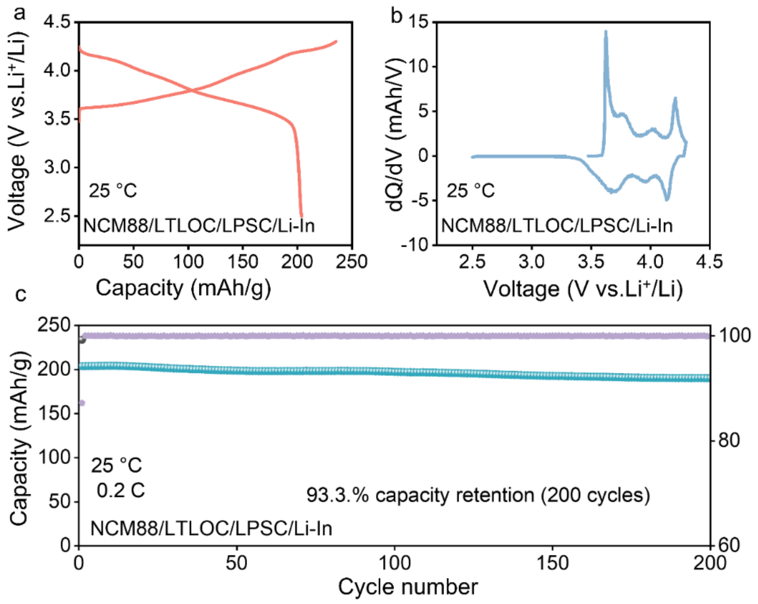

Fig. R12 | a Discharge/charge curves of the NCM88/LTLOC/LPSC/Li-In ASSB at 0.2 C. b DQ/dV curves of the NCM88/LTLOC/LPSC/Li-In ASSB at 0.2 C. c Long-term cycling performance and coulombic efficiency at 0.2 C of the NCM88/LTLOC/LPSC/Li-In ASSB at 25 ° C. (The XPS and TOF-SIMS, and SEM data of the ASSB after 200 cycles at a rate of 0.2 C (discharge specific capacity of approximately 200 mAh/g)).

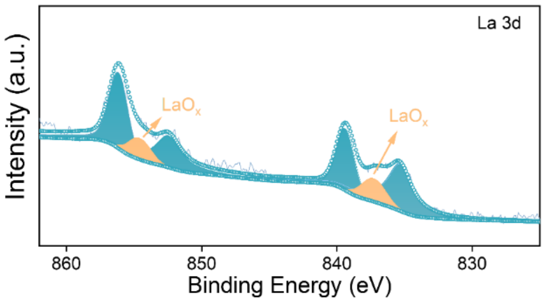

Fig. R13 | XPS spectra of La 3d after 200 cycles at a rate of 0.2 C (discharge specific capacity of approximately 200 mAh/g).

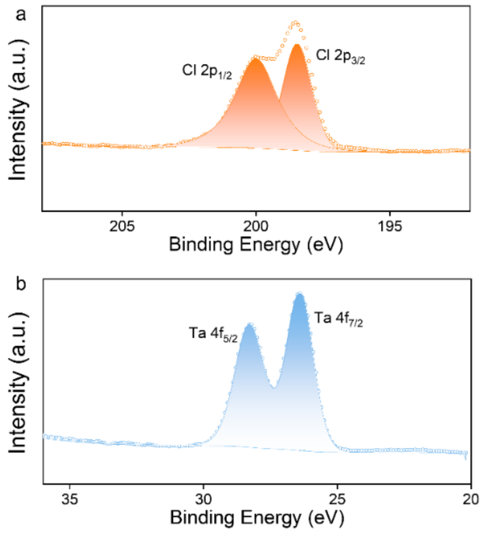

Fig. R14 | a XPS spectrum of Cl after 200 cycles at a rate of 0.2 C. **b** XPS spectrum of Ta after 200 cycles at a rate of 0.2 C (discharge specific capacity of approximately 200 mAh/g).

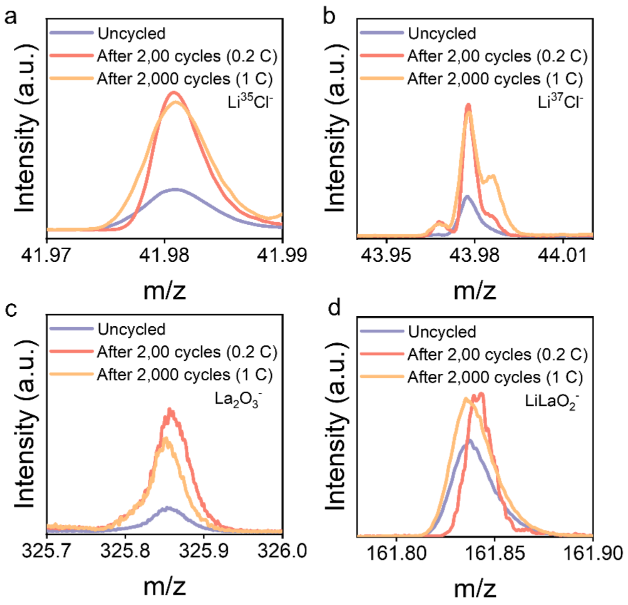

Fig. R15 | ToF-SIMS surface analysis results of **a** $Li^{35}Cl^{-}$, **b** $Li^{37}Cl^{-}$, **c** $La_2O_3^{-}$, **d** $LiLaO_2^{-}$ at a rate of 0.2 C (discharge specific capacity of approximately 200 mAh/g).

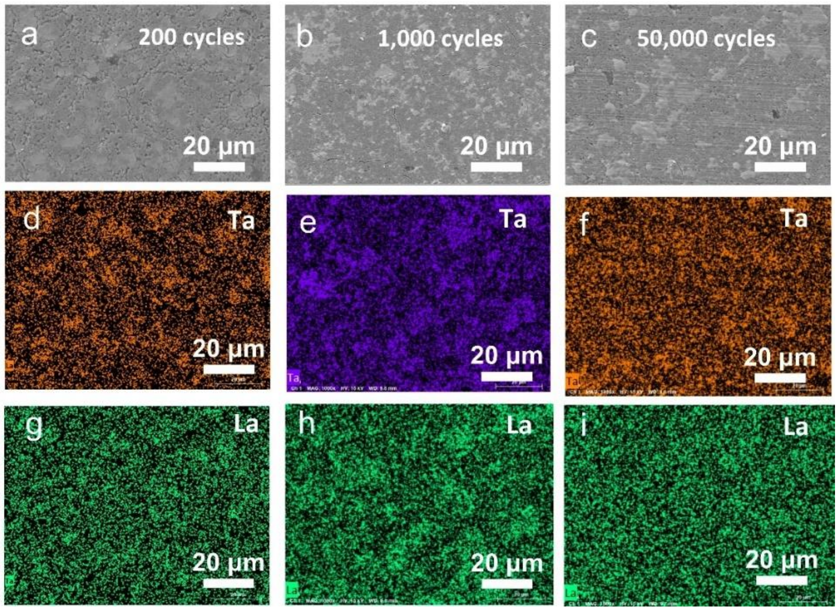

Fig. R16 | SEM and EDS images of NCM88/LTLOC ASSB after 200, 1,000, and 50,000 cycles. **a** The SEM image of NCM88/LTLOC ASSB after 200 cycles. **b** The SEM image of NCM88/LTLOC ASSB after 1,000 cycles. **c** The SEM image of NCM88/LTLOC ASSB after 50,000 cycles. **d, g** The EDS images of **a**. **e, h** The EDS images of **b**. **f, i** The EDS images of **c**. (**a, d, and g** at a rate of 0.2 C (discharge specific capacity of approximately 200 mAh/g).

1#-5. The claim about AlF_3 filler drives the Li metal stability doesn't agree with the data, the symmetrical cell obviously shows significant overpotential increases, thus interfacial reaction. Thus, the CCD test is not right.

Response: Thank you for your comments. Halide solid electrolytes are thermodynamically unstable with lithium metal, leading to reactions between them. Consequently, an increase in overpotential is observed during the early cycling of lithium symmetric cells. Literature reports indicate that electrolytes stable with lithium metal also exhibit this phenomenon of overpotential increase[5, 6, 7]. Currently, the primary approach to improving the stability of lithium metal in these systems is the use of in-situ or ex-situ interfacial layers. The stability of lithium metal discussed in this work is comparable to that reported in the literature. however, it is not an intrinsic thermodynamic stability but rather a kinetic stability achieved through the formation of an interfacial layer after cycling.

We fully agree with the reviewer's suggestions. Although we have standardized the testing procedures for lithium symmetric cells according to the literature, our survey of published studies revealed certain limitations of this method. The ability of lithium symmetric cells to cycle stably and exhibit high critical current density reflects only one aspect of the interfacial stability between the electrolyte and lithium metal. Therefore, we assess the stability of LTOC-AlF_3 against lithium metal through a multi-faceted approach combining lithium symmetric cells and all-solid-state lithium metal batteries (**Fig. R17**). Our experiments show that AlF_3, as a functional module, can improve the interfacial instability between the LTOC electrolyte and lithium metal to some extent. This intriguing phenomenon can guide us in the formation of such materials to enhance their kinetic stability with metallic lithium. We believe that in the future, through the modulation and optimisation of functional modules, we can generate a more favourable kinetically stable interfacial layer, further improving the interfacial issues between halide electrolytes and lithium metal.

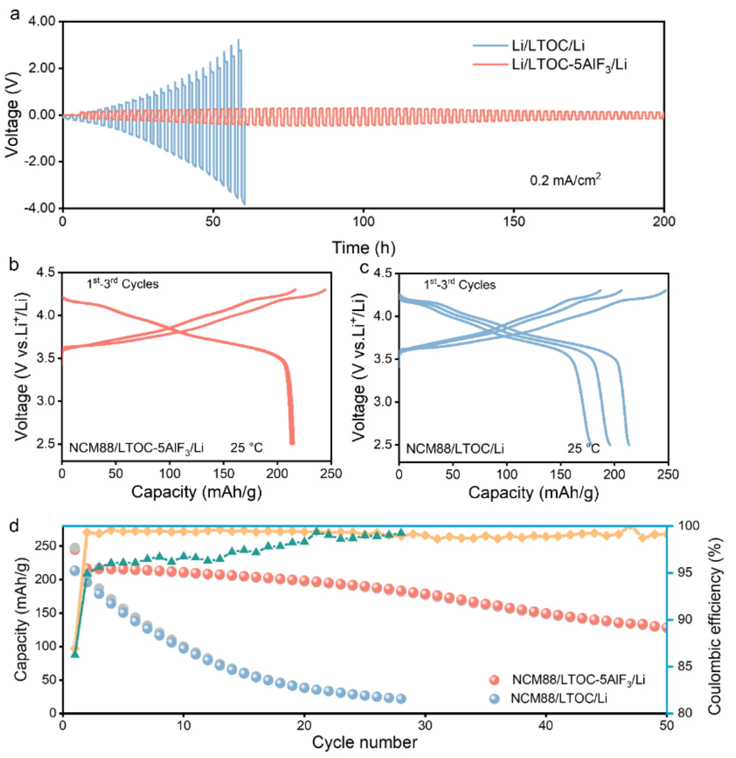

Fig. R17 | a The galvanostatic lithium stripping/plating performances of Li/LTOC/Li and Li/LTOC-5AlF_3/Li symmetric cells cycled at 0.2 mA/cm^2 with a fixed areal capacity of 0.2 mAh/cm^2. **b** Discharge/charge curves of the NCM88/LTOC-5AlF_3/Li ASSLMB at 25 °C. **c** Discharge/charge curves of the NCM88/LTOC/Li ASSLMB at 25 °C. d Long-term cycling performance and coulombic efficiency at 1C of the NCM88/LTOC-YF_3/Li and NCM88/LTOC/Li ASSLMB at 25 °C.

1#-6. Furthermore, does AlF_3 just work as filler, is any Florine exchange with Cl? And why, with high AlF_3 content, the moisture stability is better is not explained. And it is not improved much, since it is test in -30 ° C dew point dry room.

Response: Many thanks.

1. We found that the term "filler" was improperly used and could lead to ambiguity. Therefore, we have replaced "filler" with "functional module" in the text. The purpose of this work is to introduce a component that can maintain ionic transport (whether it enhances or minimally reduces ionic conductivity) while also adjusting other properties of the electrolyte. We believe that the term "functional module" is more appropriate, as it is based on a design perspective. This term aims to emphasise

the introduction of substances with regulatory functions, similar to functional additives in electrolyte liquid design. Thus, the functional module and the amorphous part are not simply a physical mixture but interact with each other. The electrolyte can be entirely amorphous (LTOC-0.2La) or may contain some crystalline phases (for example, LOTC-xLa, where x = 0.4-1.0). In both cases, we refer to $LaCl_3$ as a "functional module."

There is an exchange between F and Cl. It has been confirmed that AlF_3, as a functional module, partially interacts with the amorphous phase to form an amorphous structure in conjunction with LTOC **(Fig. R18-R20**). Therefore, it is theoretically speculated that there will be Ta-F bonds. In addition, the Ta L_3 edge EXAFS analysis reveals the presence of a Ta-F bond, which is formed through the exchange of fluoride ions (F) from AlF_3 and chloride ions (Cl) from LTOC **(Fig. R21**).

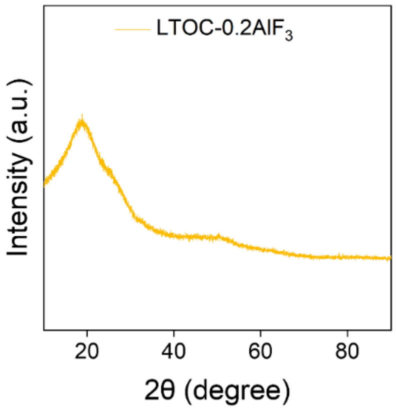

Fig. R18 | XRD patterns of LTOC-0.2AlF_3

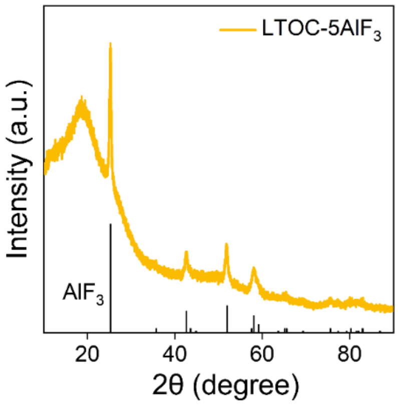

Fig. R19 | XRD patterns of LTOC-5AlF_3

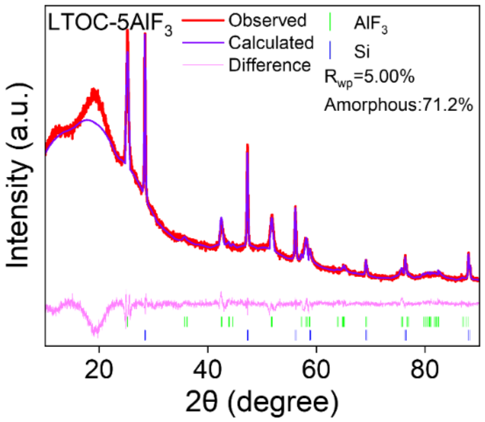

Fig. R20 | XRD refinement of LTOC-5AlF_3

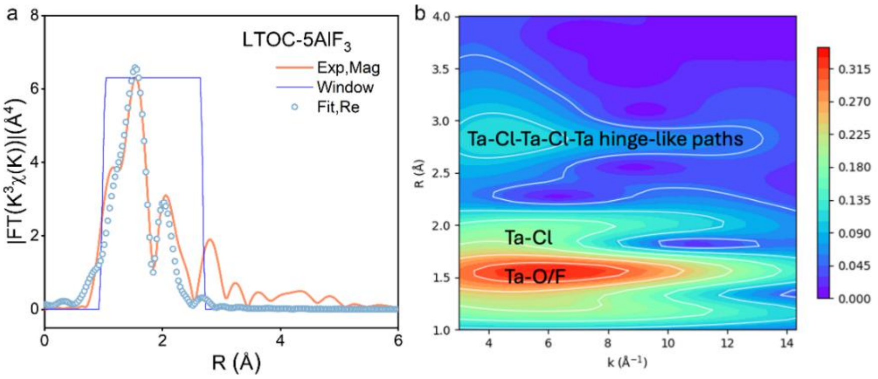

Fig. R21 | a Fourier transform of k^3-weighted Ta L_3-edge EXAFS curves of LTOC-5AlF_3, **b** WT-EXAFS of LTOC-5AlF_3 electrolyte at Ta L_3-edge. A k^3 weighting was used.

2. The reason why AlF_3 can improve the stability of solid-state electrolyte humidity is that it can simultaneously provide chemical and physical protection.

Chemical protection: It has been confirmed that AlF_3, as a functional module, partially interacts with the amorphous phase to form an amorphous structure in conjunction with LTOC. This interaction results in the formation of stronger Ta-F bonds compared to Ta-O or Ta-Cl bonds, which can mitigate reactivity with water to some extent[8, 9].

Physical protection: According to the chemical reaction equation, it can be inferred that $TaCl_5$ reacts spontaneously and violently with water, whereas AlF_3 does not react with water. Therefore, AlF_3 exhibits a greater tolerance to water compared to

$TaCl_5$. Due to the high proportion of AlF_3 introduced, it cannot completely form an amorphous structure and will exist in the form of AlF_3. Coupled with the low density of AlF_3, the portions of AlF_3 exposed to the air surface effectively reduce physical contact between the internal amorphous phase and water in the air and reduce the available surface area for moisture to attack the surface of LTOC particles without completely breaking the conduction network. Given that AlF_3 itself possesses good air stability, it can enhance the air stability of the electrolyte.

Equations for the Reaction of $TaCl_5$ and AlF_3 with H_2O (Data from Material Project):

$$0.25TaCl_5 + 0.75H_2O \longrightarrow 0.25TaCl_3O + 0.5H_3ClO \quad -0.016\ \text{eV/atom}$$

$$AlF_3 + H_2O \longrightarrow AlF_3 + H_2O \quad \text{no reaction}$$

Based on these two reasons, it can be determined that AlF_3 can improve the stability of solid-state electrolyte humidity. However, it is important to note that this strategy does not entirely resolve the issue of air stability in halide electrolytes; rather, it improves the air stability of the electrolyte to a certain extent.

3. To demonstrate the significant improvement in humidity stability compared to LTOC, we compared the ion conductivity of LTOC-5AlF_3 after a two-hour exposure to a higher dew point of -10 ° C. The results indicate that the ion conductivity retention rate of LTOC-5AlF_3 is markedly superior to that of LTOC (**Fig. R22**).

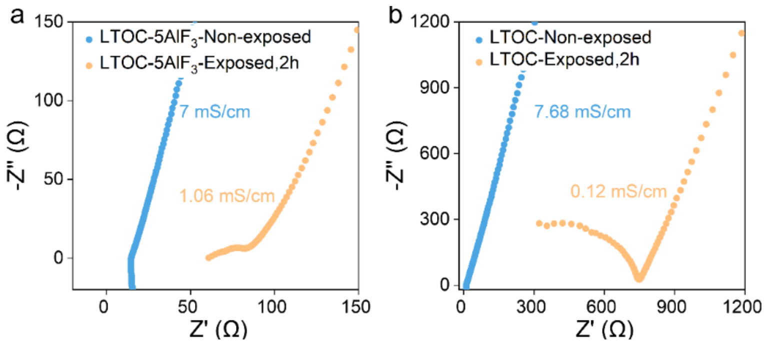

Fig. R22 | a Comparison of the EIS of LTOC-5AlF_3 before and after exposure (dew point of -10 °C). **b** Comparison of the EIS of LTOC before and after exposure (dew point of -10 °C)

Furthermore, we evaluated the humidity stability of the electrolyte by exposing it to the positive electrode material and grinding the two together in a drying room before battery assembly. This approach is particularly significant, as there are currently few reports addressing the practical application of halide electrolytes mixed with positive electrode materials under drying conditions. After grinding SSE (LTOC-5AlF_3/LTOC) and NCM88 in a drying room (dew point of -30 °C) and standing for 30 min, the ASSBs of NCM88/LTOC-5AlF_3 and NCM88/LTOC were assembled separately. The charge-discharge curve of NCM88/LTOC-5AlF_3 ASSB could be stably cycled, but NCM88/LTOC deteriorated rapidly in the second cycle with a lower capacity (**Fig. R23**). This experiment fully proves that introducing AlF_3 as a functional module contributes to enhancing humidity stability.

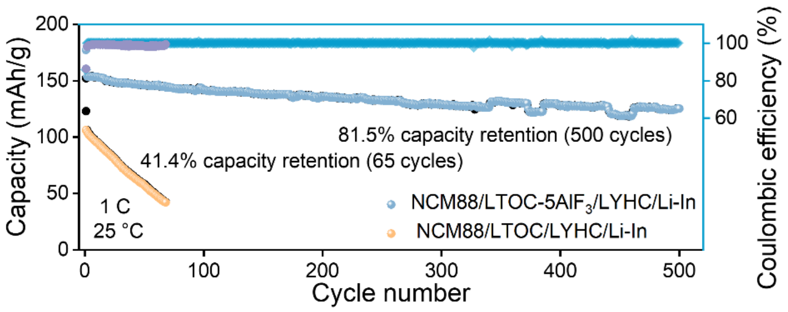

Fig R23 | Long-term cycling performance and coulombic efficiency of LTOC-5AlF_3 and LTOC in a drying room (dew point of -30 °C) after exposure to 6 h (NCM88/LTOC-5AlF_3/LYHC/Li-In and NCM88/LTOC/LYHC/Li-In).

Reviewer 2#

2#-1. This paper is reporting the role of adding inorganic filler to Li_2O-1.8$TaCl_5$ (LTOC). Although the concept was previously reported (Nat. Commun. 14, 2459, 2023) and engineering the decomposition products of solid electrolytes has been popular, the high ionic conductivity of LTOC-0.2La (13.3 mS/cm) and LTOC-5AlF_3 (7 mS/cm, despite of large molar fraction of AlF_3) is worthy to report in this journal. However, the microstructure and ionic conduction mechanism of the materials are unclear, which does not satisfy high scientific standard of Nat. Commun. The clear understanding on these materials should be provided for the publication.

2#-3. What is the role of inorganic fillers for increasing the ionic conductivity of LTOC-0.2La?

2#-5. The mechanism of high ionic conductivity of LTOC-0.2La and LTOC-5AlF_3 should be discussed. Is there any possible doping effect of La^{3+} and Al^{3+}?

2#-9. Overall, proving LTOC-5AlF_3 and LTOC-0.2La are not a common amorphous glassy material but a (nano-)mixture utilising inorganic fillers and ascertaining how ionic conductivity is increased by adding inactive inorganic fillers is crucial.

Response: Thank you for your comments. We appreciate your high recognition of the research content and the innovative aspects of this work. In this response, we address Reviewer Comments 1, 3, 5, and 9 together.

We have replaced all instances of 'fillers' with 'functional modules' to ensure consistency and rigour throughout the article. We found that the term "filler" was improperly used and could lead to ambiguity. Therefore, we have replaced "filler" with "functional module" in the text. The purpose of this work is to introduce a component that can maintain ionic transport (whether it enhances or minimally reduces ionic conductivity) while also adjusting other properties of the electrolyte. We believe that the term "functional module" is more appropriate, as it is based on a design perspective. This term aims to emphasise the introduction of substances with regulatory functions, similar to functional additives in electrolyte liquid design. Thus,

the functional module and the amorphous part are not simply a physical mixture but interact with each other. The electrolyte can be entirely amorphous (LTOC-0.2La) or may contain some crystalline phases (for example, LOTC-xLa, where x = 0.4-1.0). In both cases, we refer to $LaCl_3$ as a "functional module."

We conducted a further in-depth analysis of the mechanisms by which different functional modules enhance the performance of electrolytes. This study provides a comprehensive understanding of how functional modules improve ionic conductivity and interfacial stability, and elucidates the role of functional modules in this context. We clarified the fundamental reasons behind the ability of functional modules to regulate performance.

1. The microstructure of LTOC-xLa

$LaCl_3$ partially or fully integrates into the amorphous phase rather than simply forming a physical mixture. This is evidenced by the XRD pattern of LTOC-0.2$LaCl_3$, which displays no distinct characteristic peaks, and by TEM observations showing the absence of lattice fringes (**Fig. R24, R25**). At low concentrations of $LaCl_3$, it forms an amorphous structure with LTOC, exhibiting no distinct lattice fringes (LTOC-0.2La). As the concentration of $LaCl_3$ increases, a portion of $LaCl_3$ remains amorphous, while another portion exists in its crystalline form (LTOC-0.6La) (**Fig. R26**).

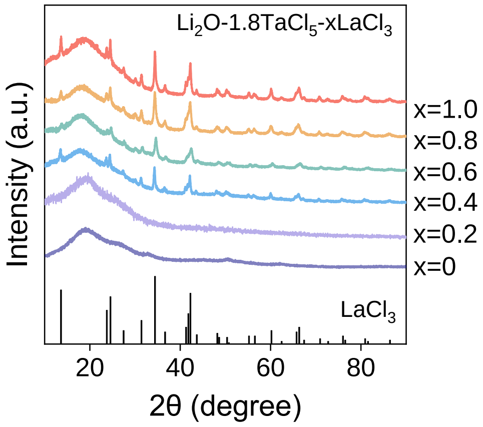

Fig. R24 | XRD patterns of LTOC-xLa with different compositions

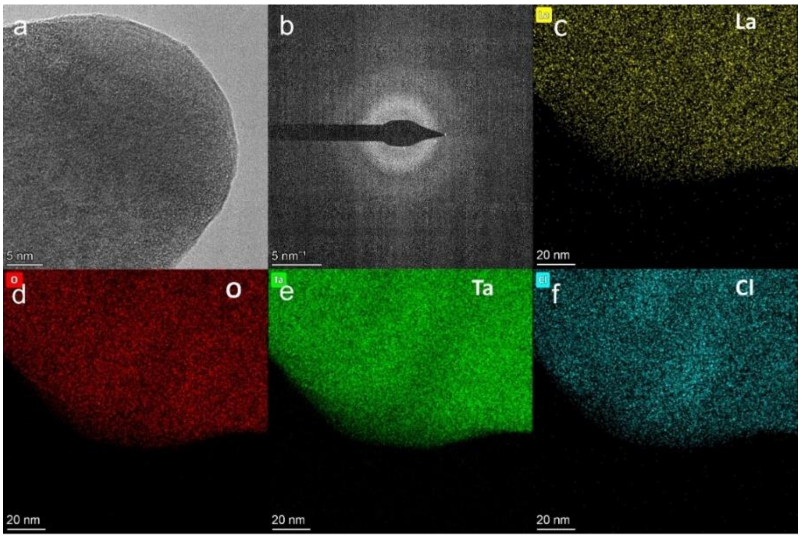

Fig. R25 | TEM images and element mapping of the image of LTLOC. **a, b** TEM image of the LTLOC. **c-f** The element mapping of the image of LTLOC.

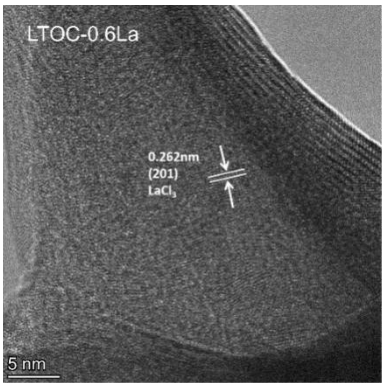

Fig. R26 | TEM images of LTOC-0.6La.

2. The ionic conduction mechanism of LTOC-xLa

We carried out a comprehensive investigation of the interaction between $LaCl_3$, serving as a functional module, and the amorphous component of LTOC by combining XRD, TEM, NMR, and first-principles calculations. The results reveal that this interaction not only entails doping effects but also enhances interfacial ionic conduction.

2.1 Doping effect

Characterisation analyses confirm that the doping effect of $LaCl_3$ is indeed effective in improving ionic conductivity. The doping effect of $LaCl_3$, particularly the

introduction of La^{3+}, plays a crucial role in enhancing ionic conductivity. However, excessive La^{3+} introduction may lead to $LaCl_3$ precipitation, which could reduce ionic conductivity. Theoretical calculations reveal that the introduction of La^{3+} alters the local environment of LTOC. The doping effect of cations leads to a differentiation in the local lithium-ion migration energy barrier of the electrolyte. The vibrational frequency of lithium ions in channels adjacent to La atoms is markedly higher than that of ions located further away, suggesting a reduced migration energy barrier in these regions, thereby facilitating ion transport (**Fig. R27**). Nevertheless, even with the introduction of $0.8LaCl_3$ (LTOC-0.8La), which exhibits an ionic conductivity of 8.7 mS/cm, this value remains higher than that of reported amorphous LTOC (7 mS/cm), indicating the persistence of the doping effect of $LaCl_3$. Thus, ion conductivity is closely related to doping.

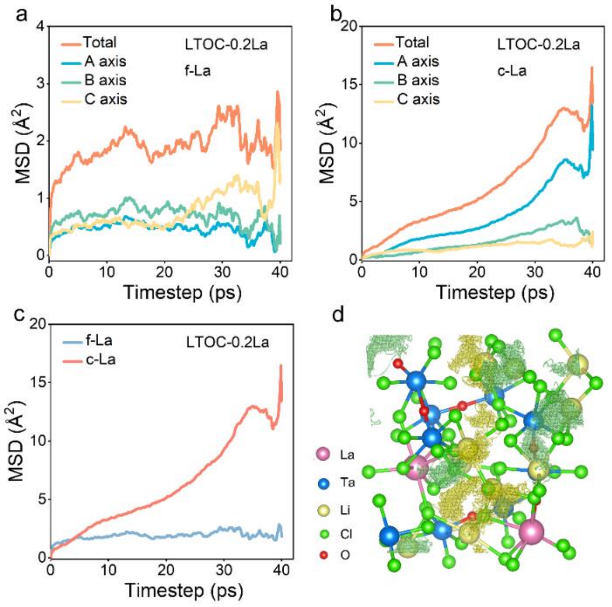

Fig. R27 | ab initio molecular dynamics (AIMD) simulations of LTOC-0.2La. **a-c** Mean Squared Displacement of Li^+ in LTOC-0.2La. **d** The structure and potential isosurface of LTOC-0.2La are visualised using VESTA. (f-La represents being far away from La, c-La represents being close to La).

2.2 Interface enhancement mechanism

The findings indicate that, beyond the doping-induced improvement in ionic

conductivity, the interfacial enhancement arising from the incorporation of functional modules is also a critical factor that warrants attention. $LaCl_3$ interacts with the amorphous phase of LTOC, facilitating lithium-ion transport at the interface. Theoretical calculations further suggest that similar interfacial enhancement effects have been observed in systems such as AlOCl-nanoLiCl and ZrO_2-Li_2ZrCl_6[2, 3]. By analysing the mean square displacement, it was found that the introduction of $LaCl_3$ significantly promotes ion transport at the interface. This highlights the importance of rapid conduction at the interface in designing functionalized modules for electrolytes. This greatly facilitates the connectivity of lithium-ion transport pathways between the bulk phase and the interface, forming a more complete, closed, and multidimensional lithium-ion transport channel, which effectively reduces the migration barrier of lithium ions and thus enhances lithium-ion conductivity (**Fig. R28**).

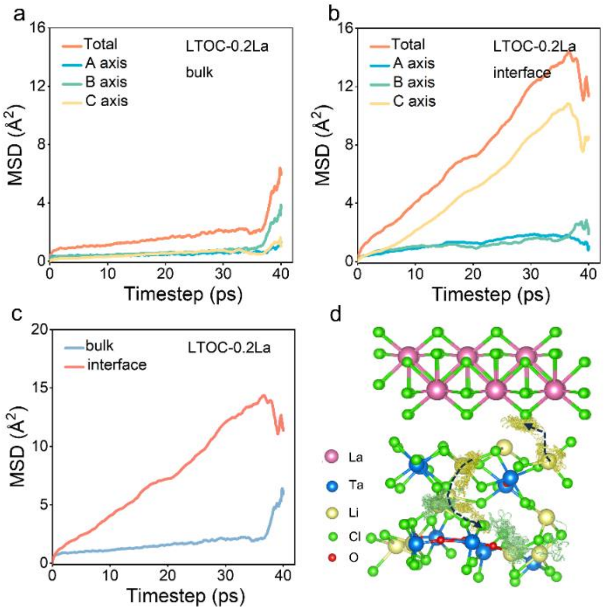

Fig. R28 | ab initio molecular dynamics (AIMD) simulations of LTOC-0.2La. **a-c** Mean Squared Displacement of Li^+ in LTOC-0.2La. **d** The structure and potential isosurface of LTOC-0.2La are visualised using VESTA.

In this work, the introduction of $LaCl_3$ serves merely as an example to illustrate the feasibility of the functional module design concept. The mechanism by which $LaCl_3$ enhances ionic conductivity is specific to the LTOC-x$LaCl_3$ system, and the effects of other functional modules on ionic transport must be evaluated individually.

Overall, the incorporation of $LaCl_3$ effectively improves ionic conductivity through the synergistic effects of promoting lithium-ion transport at the interface and the doping effect of La^{3+}.

3. The microstructure of LTOC-5AlF_3

The relatively high proportion of AlF_3 introduced, coupled with its limited solubility in the amorphous phase, renders the presence of crystalline components inevitable. By comparing the characteristic peaks from XRD with standard PDF cards, it can be inferred that the crystalline phase is AlF_3 (**Fig. R29, R30**). However, the proportion of the crystalline phase is slightly lower than the proportion of AlF_3 in the raw materials during synthesis, indicating that a portion of AlF_3 has formed an amorphous phase, while the other AlF_3 exists separately (**Fig.R31**). To verify this conclusion, we directly observed the presence of AlF_3 in LTOC-5AlF_3 through TEM (**Fig.R32**). These findings indicate that the functional modules preferentially integrate into the amorphous phase; however, when their proportion exceeds the solubility limit of the amorphous matrix, a crystalline phase begins to precipitate.

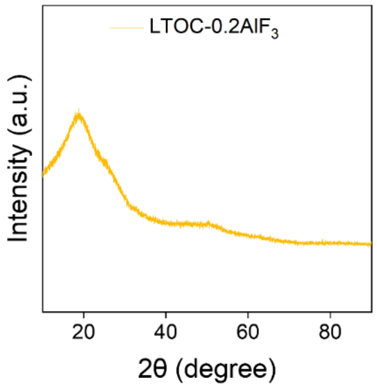

Fig. R29 | XRD patterns of LTOC-0.2AlF_3

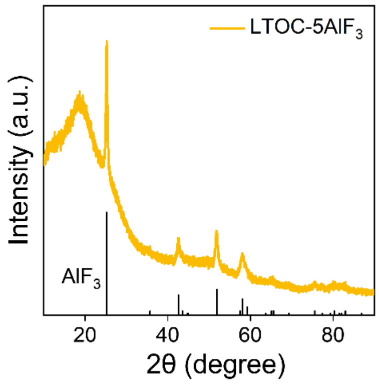

Fig. R30 | XRD patterns of LTOC-5AlF$_3$

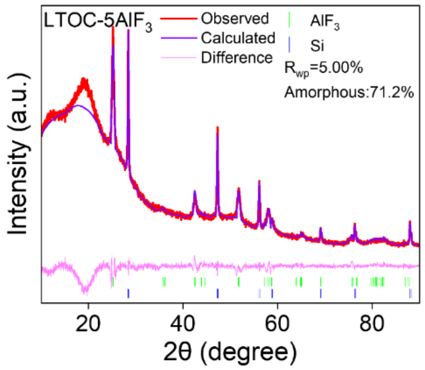

Fig. R31 | XRD refinement of LTOC-5AlF$_3$.

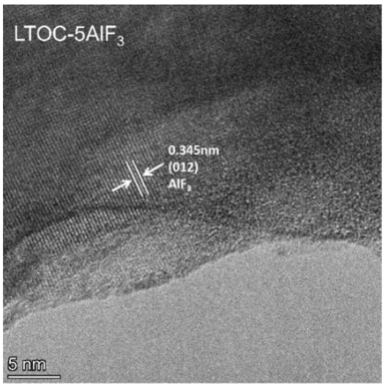

Fig. R32 | TEM images of LTOC-5AlF$_3$.

4. The ionic conduction mechanism of LTOC-5AlF$_3$

We conducted a comprehensive analysis of the interaction between AlF$_3$ as a functional module and the amorphous part of LTOC using first-principles calculations. The interaction between the functional modules and the amorphous phase not only involves doping effects but also promotes interfacial conduction.

Theoretical calculations indicate that the introduction of AlF$_3$ modifies the local environment of LTOC. The cation doping effect results in a differentiation in the local lithium-ion migration energy barrier within the electrolyte. The vibration frequency of lithium ions in the channel adjacent to Al atoms is significantly higher than that of lithium ions situated farther from Al atoms. This observation suggests that the migration energy barrier for lithium ions in this position is lower, thereby facilitating ionic transport (**Fig. R33**).

To eliminate the interference of F ions on the interface, we constructed an interface model of LTOC-$AlCl_3$. Similar to LTOC-0.2La, it was found to enhance the transport. This highlights the importance of rapid conduction at the interface in designing functionalized modules for electrolytes. This greatly facilitates the connectivity of lithium-ion transport pathways between the bulk phase and the interface, forming a more complete, closed, and multidimensional lithium-ion transport channel, which effectively reduces the migration barrier of lithium ions and thus enhances lithium-ion conductivity at the interface (**Fig. R34**).

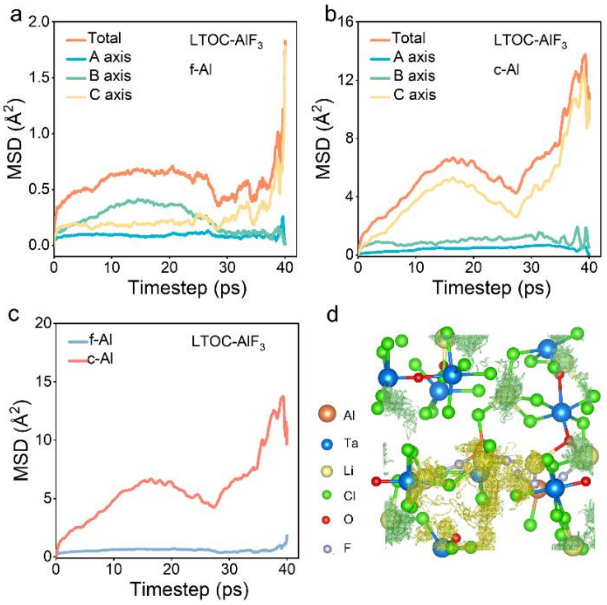

Fig. R33 | Ab initio molecular dynamics (AIMD) simulations of LTOC-AlF_3. **a-c** Mean Squared Displacement of Li^+ in LTOC-AlF_3. **d** The structure and potential isosurface of LTOC-AlF_3 are visualised using VESTA. (f-Al represents being far away from Al, c-Al represents being close to Al)

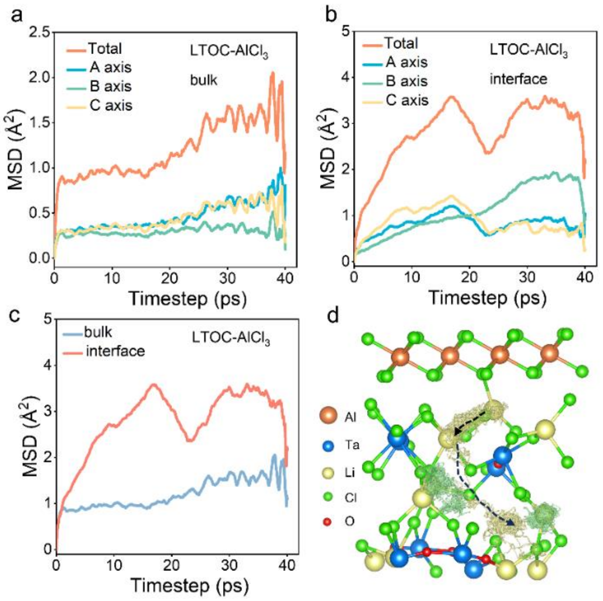

Fig. R34 | Ab initio molecular dynamics (AIMD) simulations of LTOC-$AlCl_3$. **a-c** Mean Squared Displacement of Li^+ in LTOC-$AlCl_3$. **d** The structure and potential isosurface of LTOC-$AlCl_3$ are visualised using VESTA.

2#-2. The direct evidence of the presence of intact inorganic fillers ($LaCl_3$, AlF_3, etc.) should be provide by either spectroscopy, diffractometer and/or microscopy. For example, the presence of $LaCl_3$ and AlF_3 has not been proved.

Response: Many thanks.

We found that the term "filler" was improperly used and could lead to ambiguity. Therefore, we have replaced "filler" with "functional module" in the text. The purpose of this work is to introduce a component that can maintain ionic transport (whether it enhances or minimally reduces ionic conductivity) while also adjusting other properties of the electrolyte. We believe that the term "functional module" is more appropriate, as it is based on a design perspective. This term aims to emphasise the introduction of substances with regulatory functions, similar to functional additives in electrolyte liquid design. Thus, the functional module and the amorphous part are not simply a physical mixture but interact with each other. The electrolyte can be entirely amorphous (LTOC-0.2La) or may contain some crystalline phases (for example, LOTC-xLa, where x = 0.4-1.0). In both cases, we refer to $LaCl_3$ as a "functional module."

The characteristic peaks of $LaCl_3$ and AlF_3 can be observed through X-ray diffraction (XRD), indicating the presence of $LaCl_3$ and AlF_3(**Fig. R35**). Additionally, lattice fringes corresponding to $LaCl_3$ and AlF_3 were also observed using HRTEM (**Fig. R36, R37**). The combination of XRD and HRTEM provides strong evidence for the existence of $LaCl_3$ and AlF_3.

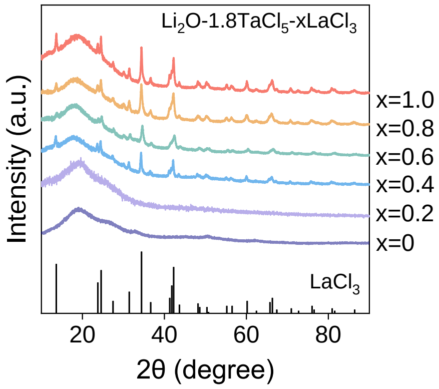

Fig. R35 | XRD patterns of LTOC-xLa with different compositions

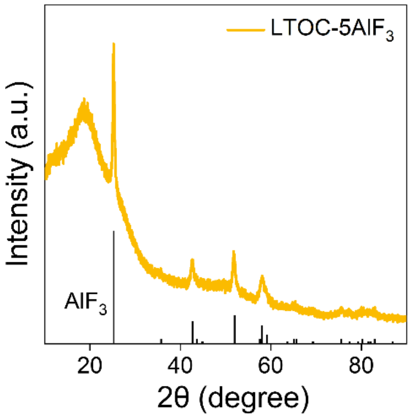

Fig. R36 | XRD patterns of LTOC-5AlF_3

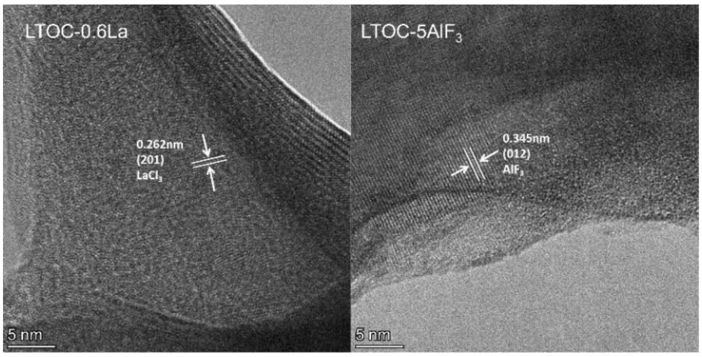

Fig. R37 | Cryo-HRTEM images of LTOC-0.6La and LTOC-5AlF$_3$

2#-4. The local environment of metal ions of the inorganic fillers (La, Al) should be verified. Does the inorganic fillers exist in intact or it form a part of amorphous matrix? XRD patterns (Fig.1d (x=0.2) and Fig. S18), and EDS mapping (Fig. S5, S6) seems to suggest that those are homogeneous amorphous materials rather than the mixtures of LTOC and inorganic fillers.

Response: Many thanks. We calibrated the amorphous content of LTOC-xLa and LTOC-5AlF$_3$ and further determined the presence of inorganic functional modules (LaCl$_3$, AlF$_3$) in the electrolyte using high-resolution transmission electron microscopy. The analyses confirm that a fraction of the functional modules enter the amorphous phase, and as their content increases, signs of saturation become evident. The combination of XRD and HRTEM provides strong evidence for the existence of LaCl$_3$ and AlF$_3$.

1. The microstructure of LTOC-xLa

LaCl$_3$ partially or fully integrates into the amorphous phase rather than simply forming a physical mixture. This is evidenced by the XRD pattern of LTOC-0.2LaCl$_3$, which displays no distinct characteristic peaks, and by TEM observations showing the absence of lattice fringes (**Fig. R38, R39**). At low concentrations of LaCl$_3$, it forms an amorphous structure with LTOC, exhibiting no distinct lattice fringes (LTOC-0.2La). As the concentration of LaCl$_3$ increases, a portion of LaCl$_3$ remains amorphous, while another portion exists in its crystalline form (LTOC-0.6La) (**Fig. R40**).

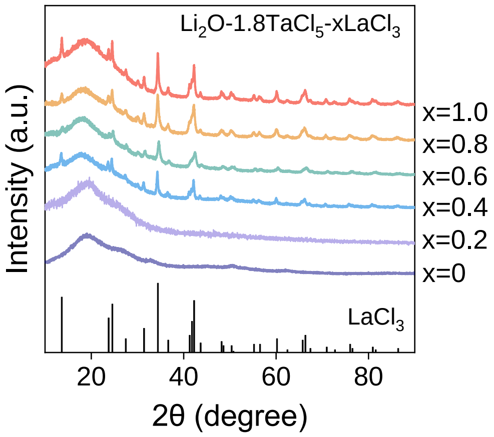

Fig. R38 | XRD patterns of LTOC-xLa with different compositions

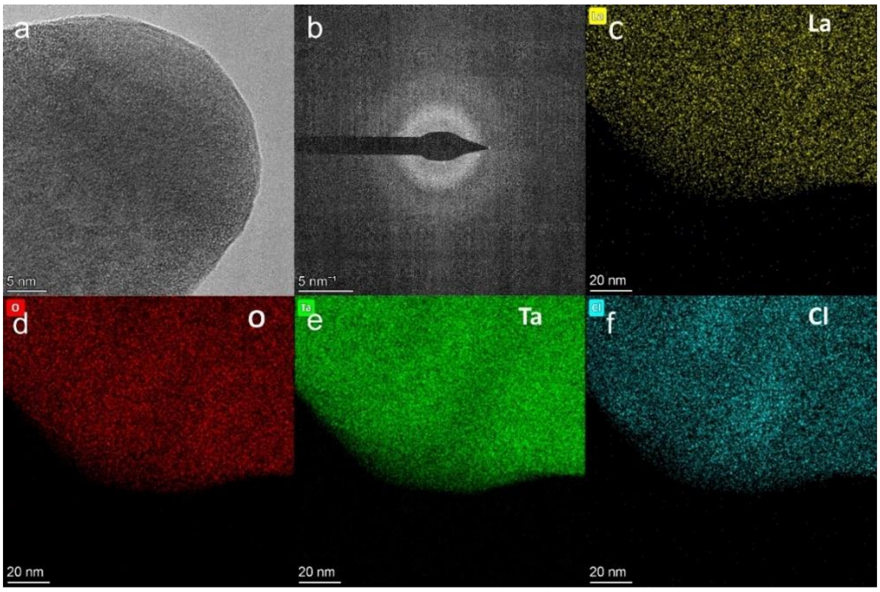

Fig. R39 | TEM images and element mapping of the image of LTLOC. **a, b** TEM image of the LTLOC. **c-f** The element mapping of the image of LTLOC.

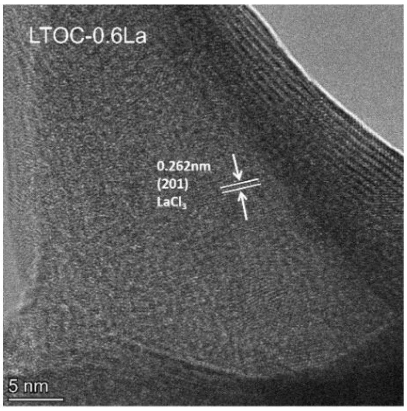

Fig. R40 | TEM images of LTOC-0.6La.

2. The microstructure of LTOC-5AlF_3

The relatively high proportion of AlF_3 introduced, coupled with its limited solubility in the amorphous phase, renders the presence of crystalline components inevitable. By comparing the characteristic peaks from XRD with standard PDF cards, it can be inferred that the crystalline phase is AlF_3 (**Fig. R41**). However, the proportion of the crystalline phase is slightly lower than the proportion of AlF_3 in the raw materials during synthesis, indicating that a portion of AlF_3 has formed an amorphous phase, while the other AlF_3 exists separately (**Fig.R42**). To verify this conclusion, we directly observed the presence of AlF_3 in LTOC-5AlF_3 through TEM (**Fig.R43**). These findings indicate that the functional modules preferentially integrate into the amorphous phase; however, when their proportion exceeds the solubility limit of the amorphous matrix, a crystalline phase begins to precipitate.

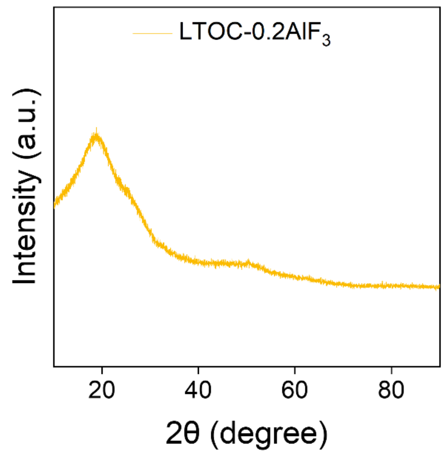

Fig. R41 | XRD patterns of LTOC-0.2AlF_3

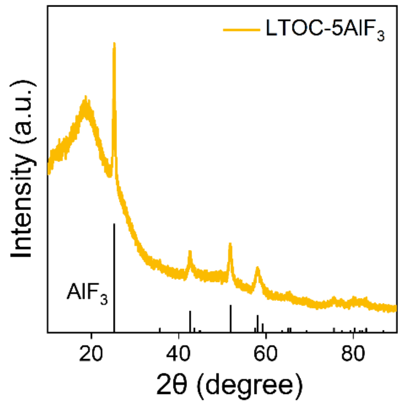

Fig. R42 | XRD patterns of LTOC-5AlF_3

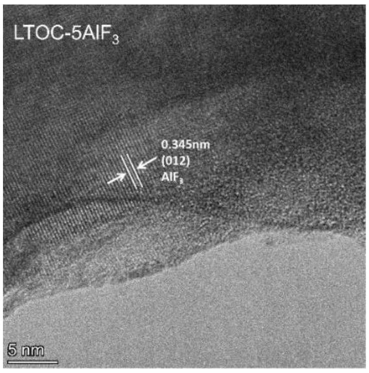

Fig. R43 | TEM images of LTOC-5AlF3.

3. Calibration of LTOC-xLa and LTOC-5AlF3 electrolyte amorphous content.

Through the calibration of the amorphous content, it can be observed that in LTOC-xLa (x=0.2, 0.4, 0.6, 0.8, and 1.0), the amorphous content gradually decreases while the proportion of the crystalline phase ($LaCl_3$) gradually increases. The proportion of the crystalline phase is slightly lower than the LaCl3/AlF3 ratio in the starting materials, suggesting that part of the LaCl3/AlF3 has incorporated into the amorphous phase, while the remainder persists as separate crystalline domains (**Fig. R44, R45**).

These findings indicate that the functional modules preferentially integrate into the amorphous phase; however, when their proportion exceeds the solubility limit of the amorphous matrix, a crystalline phase begins to precipitate.

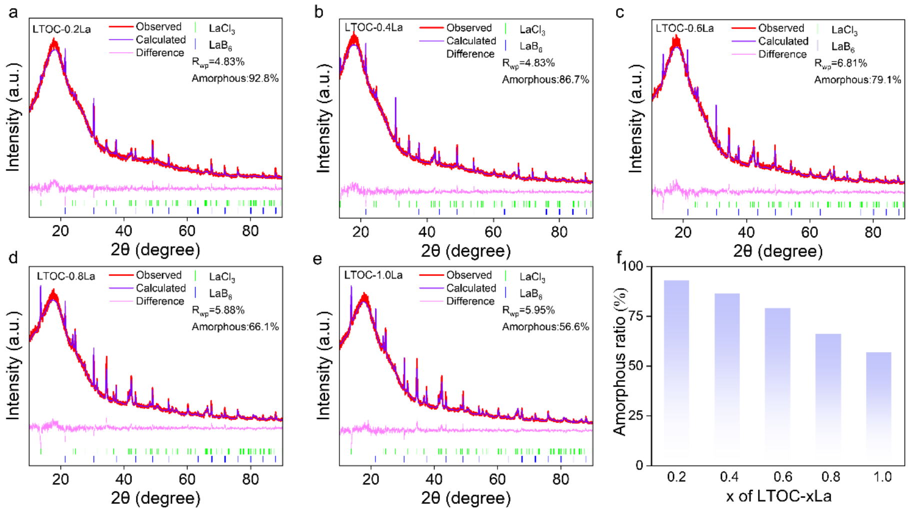

Fig. R44 | Analysis of amorphous content. a-e XRD refinement of LTOC-xLa, x = 0.2,

0.4, 0.6, 0.8 and 1.0.

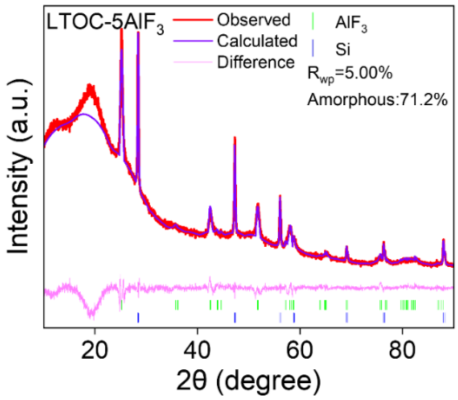

Fig. R45 | XRD refinement of LTOC-5AlF_3.

2#-6. The ^{7}Li NMR spectra of LTOC shown in Fig. 1g looks different form what shown in the reference [27]. Could you provide an explanation?

Response: Many thanks for the suggestions. We carefully analyzed the data and consider it to be accurate. The slight discrepancies compared with similar results reported in the literature are mainly attributable to differences in elemental ratio synthesis conditions and may also result from variations in the settings of the horizontal coordinate values[1].

Although the Li_2O-1.8$TaCl_5$ (LTOC) contains the same chemical elements as the xLi_2O-$TaCl_5$ ($1 \leq x \leq 2$) reported in the literature, the raw material ratios and synthesis times differ. We found that even minor changes in synthesis conditions during the process can lead to the precipitation of LiCl phases or a Li structure/chemical coordination environment similar to LiCl, although this has little impact on ionic conductivity. We found that even minor changes in synthesis conditions during the process can induce the precipitation of LiCl phases or a Li structure/chemical coordination environment similar to LiCl, although this has little impact on ionic conductivity. Owing to the limited resolution of XRD, the diffraction patterns do not display distinct characteristic peaks for these LiCl or similar to LiCl microcrystals, making it difficult to confirm the presence of trace amounts with certainty.

Additionally, the slight discrepancies from **Figure R46a** in the literature may be

due to differences in the horizontal coordinate settings. When the horizontal coordinate is aligned with that in the literature, the spectra appear consistent; however, we believe that **Figure R46b** has better readability, as the microscopic structure of the materials is a key focus of our study. To facilitate a clearer analysis of the local lithium environment, we adjusted the horizontal coordinate of the data to a smaller scale for better visualisation.

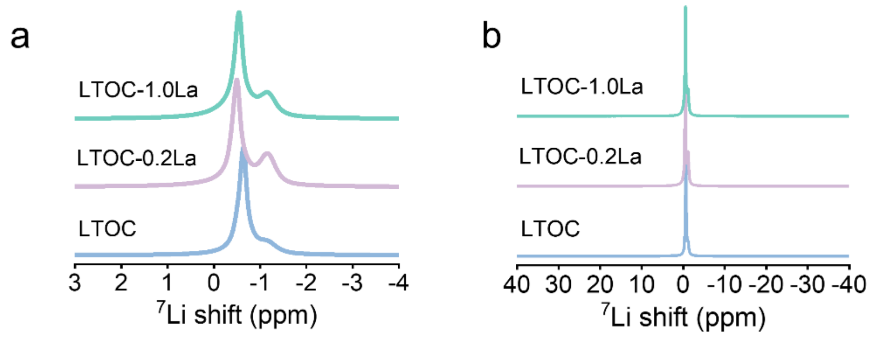

Fig. R46 | a, b ^{7}Li 1D NMR spectra of LTOC, LTOC-0.2La, and LTOC-1.0La.

2#-7. In the sentence "In the 1D ^{7}Li NMR spectra of LTOC…", the reference [27] is more suitable because the reference [20] is reporting Li NMR spectra of oxygen-free $Li_{0.5}TaCl_{5.5}$. In the sentence "The presence of a higher number of… La sites…", Is 'La' a typo?

Response: Thank you for your comments. We have corrected the format as suggested and marked them yellow.

The presence of a higher number of Cl^- ions surrounding Li sites led to lower electron density, resulting in a weaker shielding effect and downfield shifting of Li^+ resonance.

2#-8. In the sentence "The presence of a higher number of… La sites…", presumably, increasing less-electronegative Cl^- (vs. O^{2-}) may result in a stronger shielding effect. For example, the ^{7}Li NMR spectrum of LTOC-5AlF_3 (Fig. S23) shows a significant de-shielding compared to that of LTOC, possibly due to the presence of highly electro-negative F^-?

Response: Many thanks.

The shift of the ^{7}Li NMR peak in LTOC-5AlF_3 relative to LTOC is not solely due to differences in anionic electronegativity, but rather results from the combined influence of multiple factors. Any factor that alters the electron cloud density can affect the position of the ^{7}Li NMR peak.

We calibrated the amorphous content of LTOC-5AlF_3 and further determined the presence of inorganic functional modules (AlF_3) in the electrolyte using high-resolution transmission electron microscopy. The analyses confirm that a fraction of the functional modules enter the amorphous phase, and as their content increases, signs of saturation become evident. The two fillers do not simply exist as a physical mixture, as evidenced by XRD (LTOC-5AlF_3 shows no distinct characteristic peaks) (**Fig. R47**). AlF_3 interacts with the amorphous phase of LTOC, becoming part of the amorphous structure; however, the solubility of the functional modules in the amorphous phase is limited. As the filler content increases, some functional modules exist independently as a second phase, as can be observed by distinct characteristic peaks of AlF_3 in XRD (**Fig. R48, R49**). Additionally, lattice fringes corresponding to AlF_3 were also observed using HRTEM (**Fig. R50**). The combination of XRD and HRTEM provides strong evidence for the existence of AlF_3. Therefore, it is theoretically speculated that there will be Ta-F bonds. In addition, the existence of the Ta-F bond was also confirmed through the Ta L_3-edge EXAFS, indicating F exchange with Cl (**Fig. R51**).

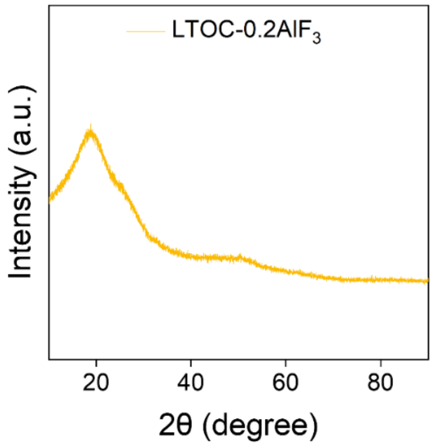

Fig. R47 | XRD patterns of LTOC-0.2AlF_3

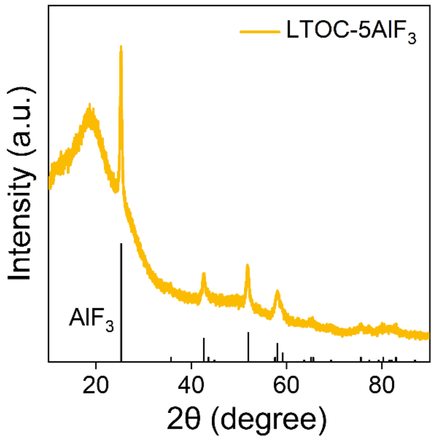

Fig. R48 | XRD patterns of LTOC-5AlF_3

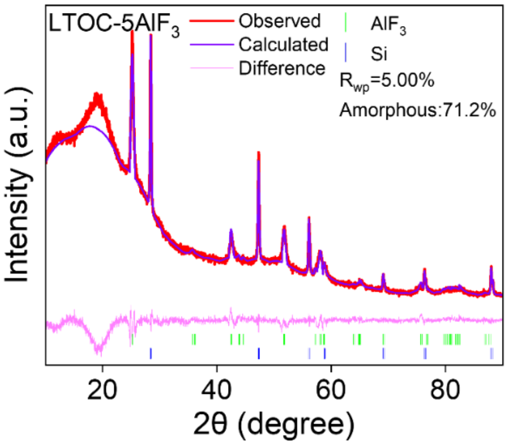

Fig. R49 | Cryo-HRTEM images of LTOC-0.6La and LTOC-5AlF_3

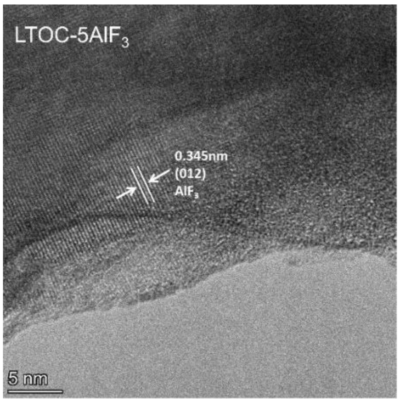

Fig. R50 | Cryo-HRTEM images of LTOC-5AlF_3

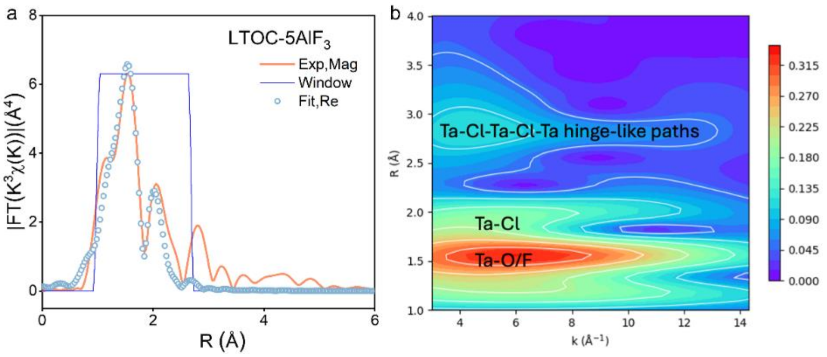

Fig. R51 | a Fourier transform of k^3-weighted Ta L_3-edge EXAFS curves of LTOC-5AlF_3, **b** WT-EXAFS of LTOC-5AlF_3 electrolyte at Ta L_3-edge. A k^3 weighting was used.

The local structure of LTOC-AlF_3 differs significantly from that of LTOC, involving the doping of cations and anions such as Al^{3+} and F^-, as well as the inability to form amorphous AlF_3. From the perspective of structure, it can be observed that there is a significant change in the Li environment (**Fig. R52**). The anion coordination near Al and Ta has changed, which will greatly affect the electron cloud density. The shift of the ^{7}Li NMR peak is caused by a combination of various factors.

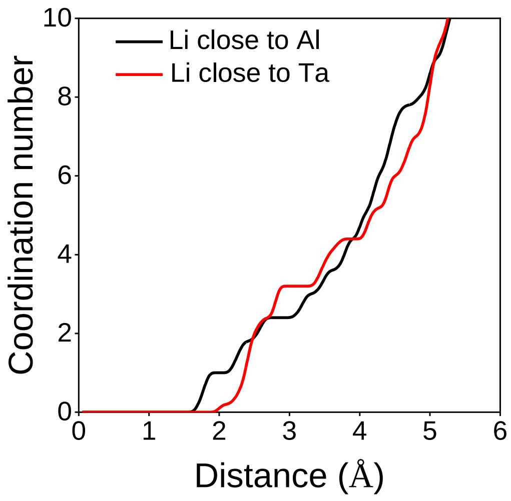

Fig. R52 | Coordination environment of Li

Reviewer 3#

3#-1. Wu et al. present a very interesting study on incorporating functional modules (“fillers”) into well-known amorphous SSE compositions to tune their properties and widen their applicability in battery systems. They present compelling cycling performance and extensive characterisation to support their materials engineering efforts, and I believe this work will spark wide interest in terms of how it enlarges the design space for SSE. However, it is also heavily phenomenological, and I have highlighted a few areas where I think more scientific insight would be helpful/of interest to readers not solely focused on battery performance.

3#-2. One primary “big-picture” question: what is the interplay between filler loading and particle size, especially for cases when the functional modules become part of a homogenous network in the composite particles? Could ‘zoning’ of the functional modules towards the exterior of, in particular, small/nanosized SSE particles impact the extent of interfacial contacts for conduction into the electrode? Correspondingly, how do the functional modules affect particle morphology? There is a series of SEM images for one LTLOC composition (Fig.S4-also, please label which one this is in the caption), but it would be helpful to also have a series spanning the full range of $LaCl_3$ incorporation levels. Especially as further nanostructuring in the composite cathode to improve conduction percolation is suggested as a future direction, and the ^7Li NMR demonstrates LiCl exsolvation, this seems worthy of addressing.

Response: Thank you for your comments. We appreciate your high recognition of the research content and the innovative aspects of this work. In this response, we address Reviewer Comments 1 and 2 together.

We conducted a detailed analysis of the interaction mechanisms between functional modules and amorphous materials, as well as the mechanisms by which functional modules enhance solid-state electrolyte performance, thereby providing deeper scientific insights. Furthermore, we explored the impact of functional modules'

proportion on the characteristics of the solid-state electrolyte (such as particle size, morphology, and Young's modulus), thereby expanding the dimensions of controllable design for functional modules.

1. The microstructure of LTOC-xLa

LaCl3 partially or fully integrates into the amorphous phase rather than simply forming a physical mixture. This is evidenced by the XRD pattern of LTOC-0.2LaCl3, which displays no distinct characteristic peaks, and by TEM observations showing the absence of lattice fringes (**Fig. R53, R54**). At low concentrations of LaCl3, it forms an amorphous structure with LTOC, exhibiting no distinct lattice fringes (LTOC-0.2La). As the concentration of LaCl3 increases, a portion of LaCl3 remains amorphous, while another portion exists in its crystalline form (LTOC-0.6La) (**Fig. R55**).

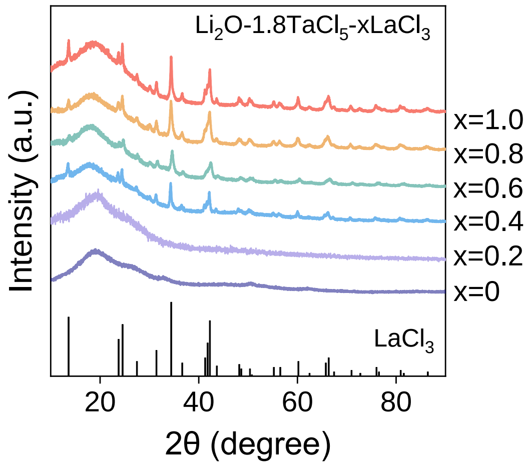

Fig. R53 | XRD patterns of LTOC-xLa with different compositions

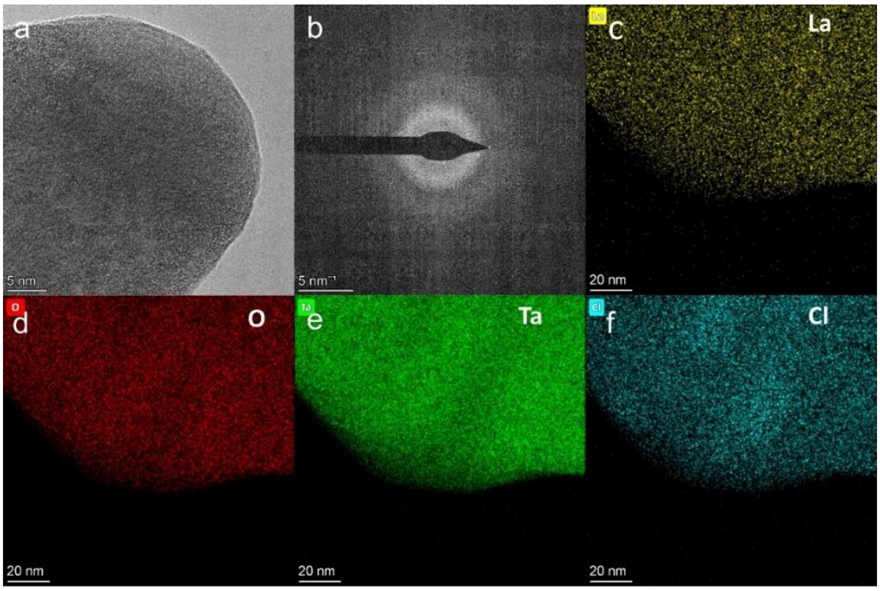

Fig. R54 | TEM images and element mapping of the image of LTLOC. **a, b** TEM image of the LTLOC. **c-f** The element mapping of the image of LTLOC.

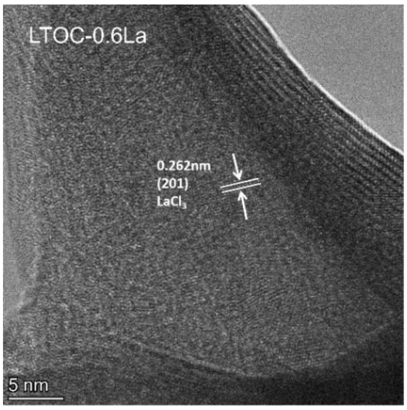

Fig. R55 | TEM images of LTOC-0.6La.

2. The ionic conduction mechanism of LTOC-xLa

We carried out a comprehensive investigation of the interaction between $LaCl_3$, serving as a functional module, and the amorphous component of LTOC by combining XRD, TEM, NMR, and first-principles calculations. The results reveal that this interaction not only entails doping effects but also enhances interfacial ionic conduction.

2.1 Doping effect

Characterisation results confirm that $LaCl_3$ doping effectively enhances the ionic conductivity. The doping effect of $LaCl_3$, particularly the introduction of La^{3+}, plays a

crucial role in enhancing ionic conductivity. However, excessive La^{3+} introduction may lead to $LaCl_3$ precipitation, which could reduce ionic conductivity. Theoretical calculations reveal that the introduction of La^{3+} alters the local environment of LTOC. The doping effect of cations leads to a differentiation in the local lithium-ion migration energy barrier of the electrolyte. The vibrational frequency of lithium ions in channels adjacent to La atoms is markedly higher than that of ions located further away, suggesting a reduced migration energy barrier in these regions, thereby facilitating ion transport (**Fig. R56**). Nevertheless, even with the introduction of $0.8LaCl_3$ (LTOC-0.8La), which exhibits an ionic conductivity of 8.7 mS/cm, this value remains higher than that of reported amorphous LTOC (7 mS/cm), indicating the persistence of the doping effect of $LaCl_3$. Thus, ion conductivity is closely related to doping.

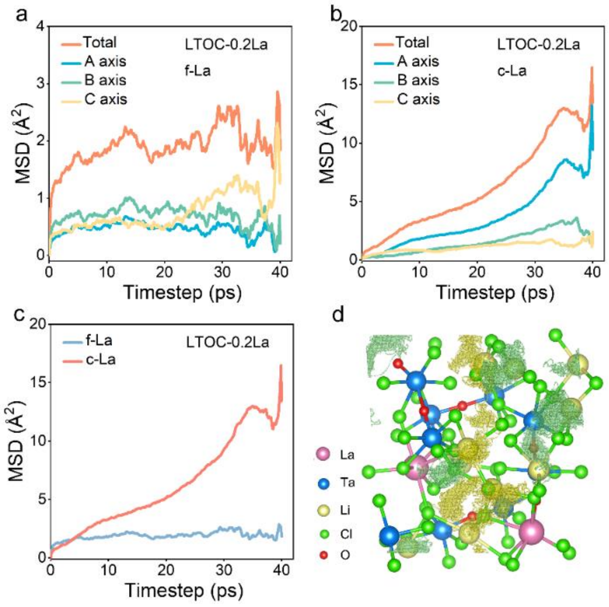

Fig. R56 | ab initio molecular dynamics (AIMD) simulations of LTOC-0.2La. **a-c** Mean Squared Displacement of Li^+ in LTOC-0.2La. **d** The structure and potential isosurface of LTOC-0.2La are visualised using VESTA. (f-La represents being far away from La, c-La represents being close to La).

2.2 Interface enhancement mechanism.

The findings indicate that, beyond the doping-induced improvement in ionic

conductivity, the interfacial enhancement arising from the incorporation of functional modules is also a critical factor that warrants attention. $LaCl_3$ interacts with the amorphous phase of LTOC, facilitating lithium-ion transport at the interface. Theoretical calculations further suggest that similar interfacial enhancement effects have been observed in systems such as AlOCl-nanoLiCl and ZrO_2-Li_2ZrCl_6[2, 3]. By analysing the mean square displacement, it was found that the introduction of $LaCl_3$ significantly promotes ion transport at the interface. This highlights the importance of rapid conduction at the interface in designing functionalized modules for electrolytes. This greatly facilitates the connectivity of lithium-ion transport pathways between the bulk phase and the interface, forming a more complete, closed, and multidimensional lithium-ion transport channel, which effectively reduces the migration barrier of lithium ions and thus enhances lithium-ion conductivity (**Fig. R57**).

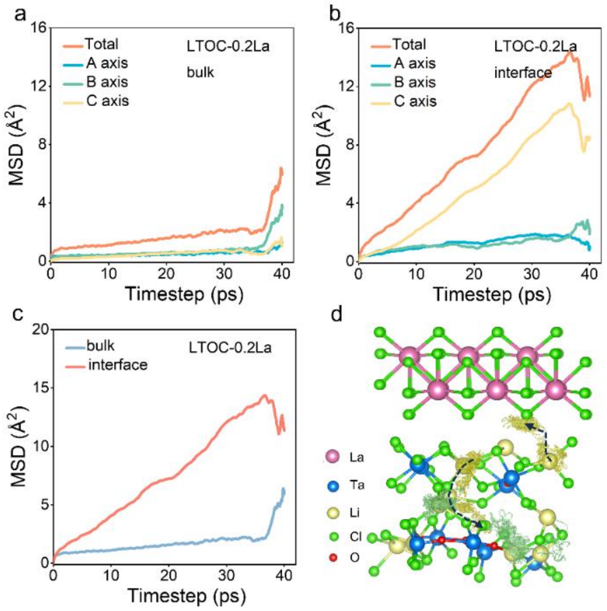

Fig. R57 | ab initio molecular dynamics (AIMD) simulations of LTOC-0.2La. **a-c** Mean Squared Displacement of Li^+ in LTOC-0.2La. **d** The structure and potential isosurface of LTOC-0.2La are visualised using VESTA.

In this work, the introduction of $LaCl_3$ serves merely as an example to illustrate the feasibility of the functional module design concept. The mechanism by which $LaCl_3$ enhances ionic conductivity is specific to the LTOC-x$LaCl_3$ system, and the effects of other functional modules on ionic transport must be evaluated individually.

Overall, the incorporation of $LaCl_3$ effectively improves ionic conductivity through the synergistic effects of promoting lithium-ion transport at the interface and the doping effect of La^{3+}.

3. The microstructure of LTOC-5AlF_3

The relatively high proportion of AlF_3 introduced, coupled with its limited solubility in the amorphous phase, renders the presence of crystalline components inevitable. By comparing the characteristic peaks from XRD with standard PDF cards, it can be inferred that the crystalline phase is AlF_3 (**Fig. R58, R59**). However, the proportion of the crystalline phase is slightly lower than the proportion of AlF_3 in the raw materials during synthesis, indicating that a portion of AlF_3 has formed an amorphous phase, while the other AlF_3 exists separately (**Fig.R60**). To verify this conclusion, we directly observed the presence of AlF_3 in LTOC-5AlF_3 through TEM (**Fig.R61**). These findings indicate that the functional modules preferentially integrate into the amorphous phase; however, when their proportion exceeds the solubility limit of the amorphous matrix, a crystalline phase begins to precipitate.

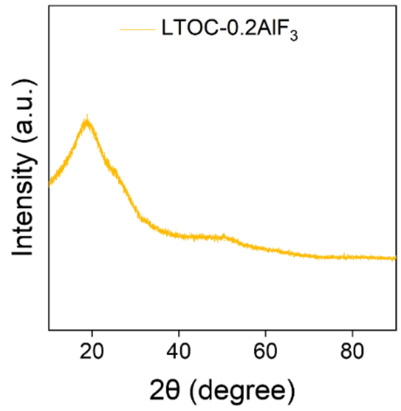

Fig. R58 | XRD patterns of LTOC-0.2AlF_3

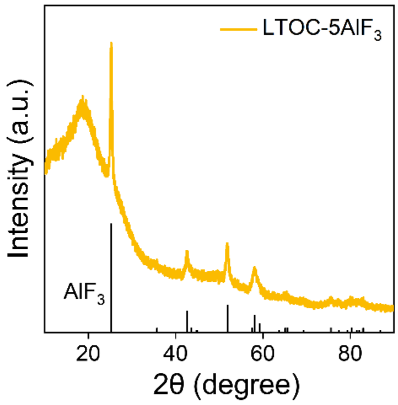

Fig. R59 | XRD patterns of LTOC-5AlF3

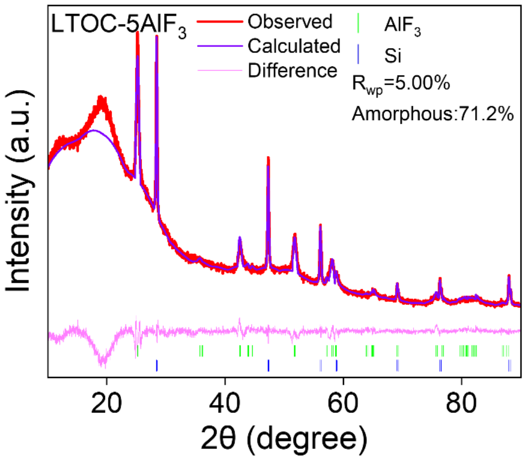

Fig. R60 | XRD refinement of LTOC-5AlF3.

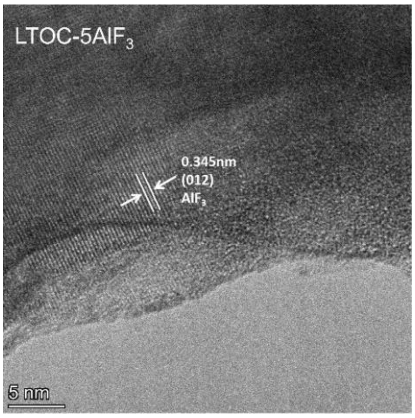

Fig. R61 | TEM images of LTOC-5AlF3.

4. The ionic conduction mechanism of LTOC-5AlF3

We conducted a comprehensive analysis of the interaction between AlF_3 as a functional module and the amorphous part of LTOC using first-principles calculations. The interaction between the functional modules and the amorphous phase not only

involves doping effects but also promotes interfacial conduction.

Theoretical calculations indicate that the introduction of AlF_3 modifies the local environment of LTOC. The cation doping effect results in a differentiation in the local lithium-ion migration energy barrier within the electrolyte. The vibration frequency of lithium ions in the channel adjacent to Al atoms is significantly higher than that of lithium ions situated farther from Al atoms. This observation suggests that the migration energy barrier for lithium ions in this position is lower, thereby facilitating ionic transport (**Fig. R62**).

To eliminate the interference of F ions on the interface, we constructed an interface model of LTOC-$AlCl_3$. Similar to LTOC-0.2La, it was found to enhance the transport. This highlights the importance of rapid conduction at the interface in designing functionalized modules for electrolytes. This greatly facilitates the connectivity of lithium-ion transport pathways between the bulk phase and the interface, forming a more complete, closed, and multidimensional lithium-ion transport channel, which effectively reduces the migration barrier of lithium ions and thus enhances lithium-ion conductivity at the interface (**Fig. R63**).

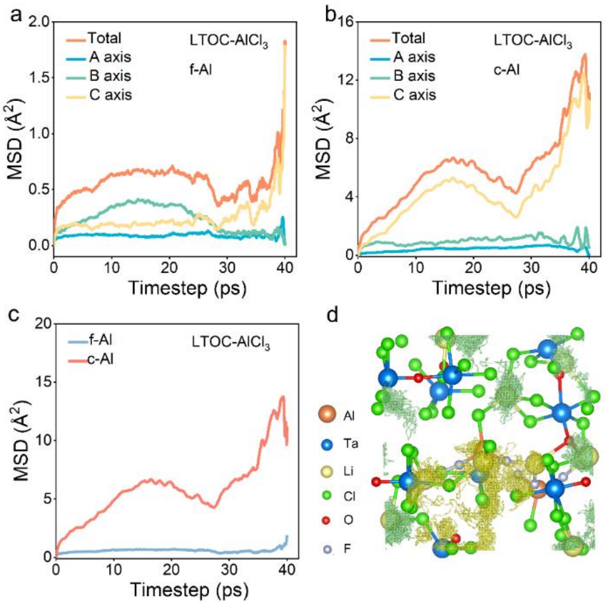

Fig. R62 | Ab initio molecular dynamics (AIMD) simulations of LTOC-AlF_3. **a-c** Mean Squared Displacement of Li^+ in LTOC-AlF_3. **d** The structure and potential

isosurface of LTOC-AlF_3 are visualised using VESTA. (f-Al represents being far away from Al, c-Al represents being close to Al)

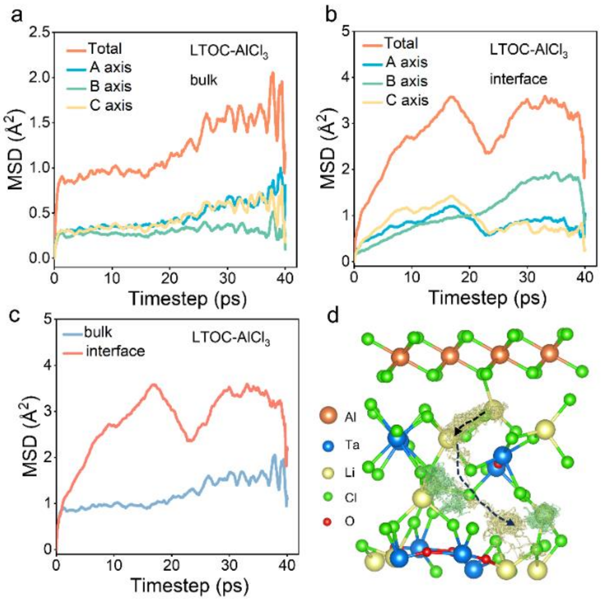

Fig. R63 | Ab initio molecular dynamics (AIMD) simulations of LTOC-$AlCl_3$. **a-c** Mean Squared Displacement of Li^+ in LTOC-$AlCl_3$. **d** The structure and potential isosurface of LTOC-$AlCl_3$ are visualised using VESTA.

5. The relationship between filler loading and electrolyte particle size remains unclear; however, it plays a critical role in determining the physical contact and chemical performance between the electrolyte and the cathode in a composite electrode. To investigate this, we characterized a series of solid-state electrolytes, LTOC-xLa (x = 0.2, 0.4, 0.6, 0.8, and 1.0), with varying proportions of functional filler ($LaCl_3$), focusing on their particle size distribution, morphology, Young's modulus, and the electrochemical performance of all-solid-state batteries assembled under identical conditions using these electrolytes. This aims to comprehensively understand the effects of different proportions of functional fillers on the solid-state electrolytes themselves, including particle size, morphology, and Young's modulus, as well as their impact on the contact and conductivity at the composite electrode interface. The particle size distribution indicates that the particle size of LTOC is slightly larger, and the introduction of $LaCl_3$ results in a slight reduction in particle size; however, the overall difference is minor, with D50 values ranging between 6-9

μm (**Fig. R64**). SEM observations reveal that the particles are irregular in shape, with no significant differences in morphology (**Fig. R65**).

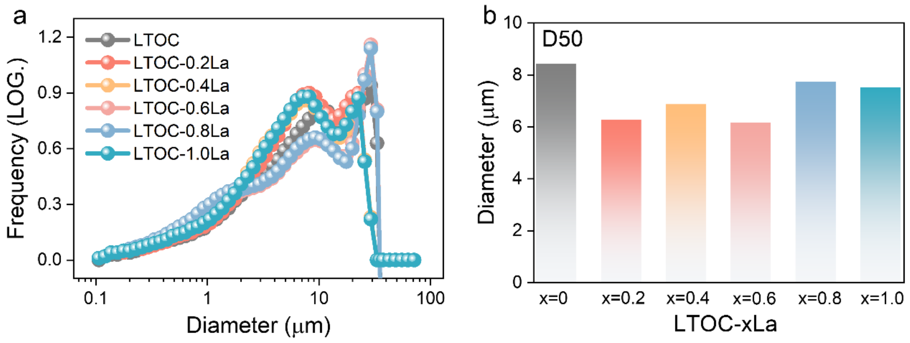

Fig. R64 | a The diameter of LTOC-xLa (x = 0, 0.2, 0.4, 0.6, 0.8, 1.0) SSEs. **b** Comparison of particle size distribution of LTOC-xLa (x = 0, 0.2, 0.4, 0.6, 0.8, 1.0) SSEs.

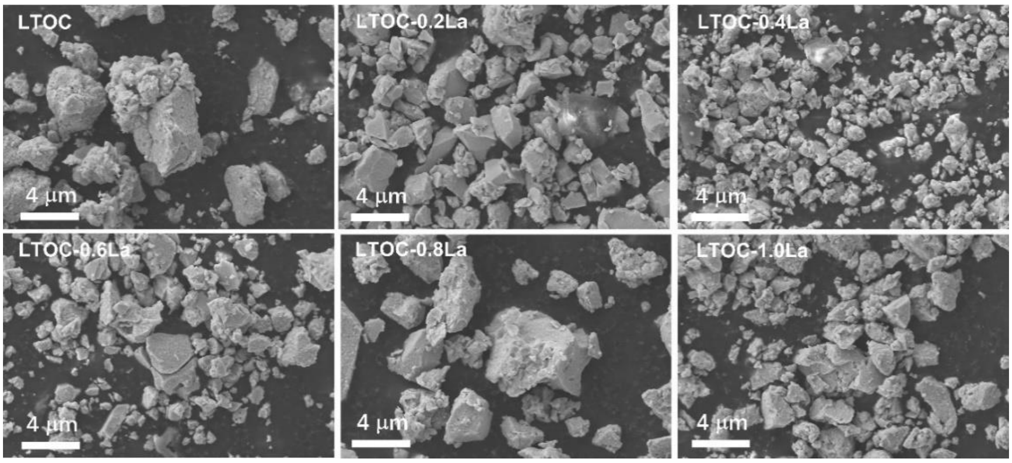

Fig. R65 | SEM images of LTOC-xLa (x = 0, 0.2, 0.4, 0.6, 0.8, 1.0) SSEs

As the value of x increases, the Young's modulus exhibits a trend of first decreasing and then increasing (**Table R1**). Additionally, the initial discharge specific capacity of the all-solid-state battery assembled with the electrolyte is close to 200 mAh/g (0.2 C). After 200 cycles, the capacity retention rate shows slight differences, with NCM88/LTOC-1.0La/LPSC/Li-In demonstrating the poorest capacity retention (**Fig. R66**). Although the experimental data do not yet allow definitive conclusions to be drawn, they suggest that the molar ratio of functional modules exerts a measurable influence on the properties of the electrolyte and the performance of all-solid-state batteries employing it. This represents a promising direction for further investigation.

Table R1. The Young's modulus of LTOC-xLa (x = 0, 0.2, 0.4, 0.6, 0.8, 1.0) SSEs

SSE	Density (g/cm^{-3})	P-wave velocity (m/s)	S-wave velocity (m/s)	Young's modulus (GPa)
LTOC	3.5287	2510.07	1157.89	12.914
LTOC-0.2La	3.4341	2885.24	1079.75	11.359
LTOC-0.4La	3.3794	2161.49	1104.76	10.915
LTOC-0.6La	3.3013	2210.52	1127.52	11.114
LTOC-0.8La	3.2078	2820.14	1187.88	12.603
LTOC-1.0La	3.0995	2684.21	1378.38	15.106

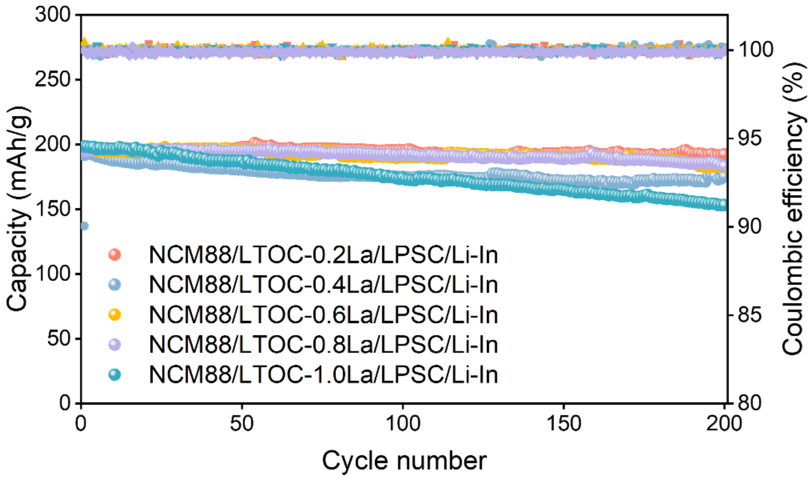

Fig. R66 | Electrochemical performance of NCM88/LTOC-xLa/LPSC/Li-In at 0.2 C

6. The functional module significantly influences the electrode interface. After prolonged cycling, the electrode interface continues to maintain close contact, which is a crucial factor in achieving long-term stable cycling (**Fig. R67**). Furthermore, the high proportion of highly active materials combined with this electrolyte and the high load of the battery contribute to its outstanding performance (**Fig. R68**). Although it remains uncertain whether the functional module is the sole influencing factor, it is evident that it plays a vital role.

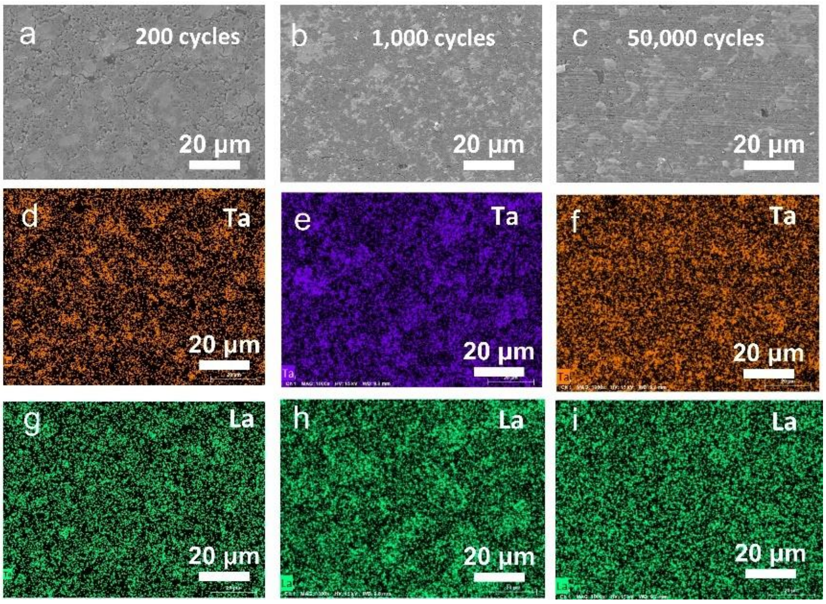

Fig. R67 | SEM and EDS images of NCM88/LTLOC ASSB after 200, 1,000, and 50,000 cycles. **a** The SEM image of NCM88/LTLOC ASSB after 200 cycles. **b** The SEM image of NCM88/LTLOC ASSB after 1,000 cycles. **c** The SEM image of NCM88/LTLOC ASSB after 50,000 cycles. **d, g** The EDS images of **a**. **e, h** The EDS images of **b**. **f, i** The EDS images of **c**. (**a, d, and g** at a rate of 0.2 C (discharge specific capacity of approximately 200mAh/g).

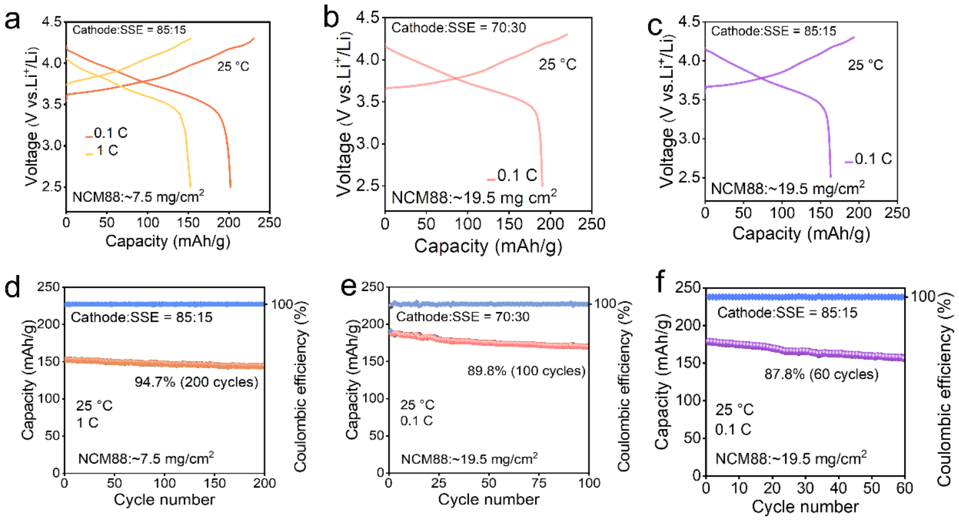

Fig. S68 | Electrochemical performance of ASSB of NCM88/LTLOC/LPSC/Li-In ASSB at 4.3 V (vs.Li^+/Li). **a** Discharge/charge curves of NCM88/LTLOC ASSB (85 wt% NCM88 and 15 wt% SSE, NCM88: ~7.5 mg/cm^2). **b** Discharge/charge curves of

NCM88/LTLOC ASSB (70 wt% NCM88 and 30 wt% SSE, NCM88: ~19.5 mg/cm^2). **c** Discharge/charge curves of NCM88/LTLOC ASSB (85 wt% NCM88 and 15 wt% SSE, NCM88: ~19.5 mg/cm^2). **d** Long-term cycling performance and coulombic efficiency of NCM88/LTLOC ASSB (85 wt% NCM88 and 15 wt% SSE, NCM88: ~7.5 mg/cm^2). **e** Long-term cycling performance and coulombic efficiency of NCM88/LTLOC ASSB (70 wt% NCM88 and 30 wt% SSE, NCM88: ~19.5 mg/cm^2). **f** Long-term cycling performance and coulombic efficiency of NCM88/LTLOC ASSB (85 wt% NCM88 and 15 wt% SSE, NCM88: ~19.5 mg/cm^2)

7. To analyse how functional modules affect the morphology of electrolytes, we characterised the morphology of a series of LTOC La electrolytes. SEM observations reveal that SEM observations reveal that the particle size of LTOC-xLa is slightly lower than that of LTOC (**Fig. R65**).

8. Figure 4 (original Figure 3) clearly indicates the composition of the electrolyte.

9. Thank you for your valuable suggestions. Your recommendation that "further nanostructuring in the composite cathode to improve conduction percolation is suggested as a future direction" is crucial for our future research. We will build upon this work to further investigate the critical role of nanostructuring in the composite cathode. Thank you once again.

3#-3. Line 65: “doped halide SSEs with superior characteristics” – a bit more specificity would be helpful, in the sense of which “superior characteristics” are intended to be highlighted (especially with four separate references cited towards this point).

Response: Many thanks. We have added the following content and revised the manuscript, and marked them yellow.

Doping with Ta^{5+} leads to the formation of a three-dimensional migration network within the one-dimensional Li^+ diffusion channels of the $LaCl_3$ structure, effectively enhancing ionic transport[5]. Furthermore, doping can regulate ionic conductivity by lowering the migration barrier. This can be achieved either by tuning

the mixing entropy of the material system or by modulating the covalency of the bonds between metals and halides.[10, 11]. In addition to improving ionic conductivity, doping is essential for regulating the chemical and electrochemical stability of the electrolyte. Research has demonstrated that the O-doping strategy can partially substitute Cl in Li_3InCl_6, thereby simultaneously enhancing its oxidative stability and interfacial stability with sulfide electrolytes[12].

3#-4. Line 89-101: This section of the final intro paragraph largely repeats similar content as the abstract; I think it would be improved if the authors instead offered additional details at this stage to rationalize why one would pick, e.g., an oxide versus a chloride functional module, and what drives the selection of one over another in trying to regulate a given property of the SSE (essentially, previewing the results a bit more specifically, more like the beginning of the paragraph where $LaCl_3$ is used as an example, in order to reduce the repetition a bit).

Response: Many thanks for the suggestions. The content has been modified as follows, and the manuscript and marked them yellow.

Amorphous SSEs offer several advantages, including reduced structural limitations, a broader range of component modifications, and an expanded material design space[13]. The compositional design of amorphous halide (or oxyhalide) SSEs with high ionic conductivities facilitates the optimisation of SSE performance. This study introduces a novel strategy for SSE design by categorising SSEs into two modules: the transport module (amorphous matrix) and the functional module (filler). This approach allows for functional customisation through fillers while preserving the high ionic conductivity of the amorphous component. The modular design enables diverse functionalities by incorporating various fillers. The introduction of $LaCl_3$, which possesses a large-sized UCl_3-type structure with a $P6_3/m$ space group, aims to validate the feasibility of the functional module design concept. Incorporating $LaCl_3$ enhances ionic transport at the interface between the amorphous region and the functional module, thereby sustaining high ionic conductivity. Furthermore, the Li_2O-$1.8TaCl_5$-$0.2LaCl_3$ (LTLOC) SSE containing La elements forms a highly stable

interfacial layer with excellent kinetics in situ with $LiNi_{0.88}Co_{0.09}Mn_{0.03}O_2$ (NCM88), allowing the ASSBs to demonstrate outstanding rate capability, cycling stability, and low-temperature performance. This study demonstrates the ability to achieve ultralong stable cycles (exceeding 50,000 cycles) and ultralow temperature operation (over 4,900 cycles at -30 ° C) in ASSBs. A variety of fillers, including chloride, oxide, and fluoride, can be integrated into the SSEs to preserve the amorphous state while maintaining high ionic conductivities. This finding further substantiates the universality of the design of amorphous functional modules. The inclusion of low-cost and low-density AlF_3 underscores the advantages of this design approach, as it permits a substantial proportion of fluoride to be introduced without compromising ionic conductivity. The composition Li_2O-1.8$TaCl_5$-5AlF_3 (LTOC-5AlF_3) demonstrates high ionic conductivity, stability under humid conditions, resistance to high voltage, and compatibility with lithium metal. This research utilises various functional modules in material design to effectively regulate multiple functions, including ionic conductivity, electrochemical stability, interface design, humidity stability, high-pressure stability, and lithium metal stability, thereby showcasing the progress and versatility of functional module design.

3#-5. Line 143: The site labelled as Li2 in the ^{7}Li MAS-NMR is most certainly exsolvated LiCl, which is commonly detected in halide SSE by ^{7}Li NMR. While the shift of the main amorphous peak with incorporation of $LaCl_3$ is most likely the result of the polarizability effect that the authors identify, it does also suggest that the incorporation of the functional module is simultaneously creating additional pockets of LiCl, as evidenced by the growth in the intensity of this site (either too small to be diffractable, or too poorly ordered). While it is possible that these sites are part of the conduction network and do contribute to chemical exchange that is detected by the 2D EXSY, the cross-peaks there could also be due to spin diffusion (as the EXSY experiment is functionally equivalent to NOESY). The primary way to distinguish these cases is to perform the EXSY at a series of temperatures; spin diffusion is relatively insensitive to increasing temperature, whereas chemical exchange will show

strong thermal activation (evidenced by increased cross peak volume at the same mixing time), especially as these are fast ion conductors. Further, there is an added dividend: the activation energy associated with the increased exchange rate will provide a complementary energy scale that is comparatively more local than the activation energy derived from macroscopic conductivity measurements. Also, it would be helpful if the authors marked the mixing times on Figures 1h,i (or included them in the caption).

Response: Many thanks.

1. After careful analysis in comparison with the literature, we found that the peak at this position can be attributed to LiCl or a Li structure/chemical coordination environment similar to LiCl, indicating that the addition of the functional module ($LaCl_3$) interacts with the amorphous part, leading to the precipitation of LiCl or similar to LiCl.

2. We sincerely apologise for not being able to schedule the variable-temperature NMR tests before responding to the review comments. We fully agree with your point that the EXSY at a series of temperatures could differentiate whether the phenomena observed in the figures are due to chemical exchange or spin diffusion. However, due to limited conditions in our laboratory, we do not have access to this equipment, and the scheduling times for other laboratories that meet the experimental conditions are quite lengthy, which has hindered our ability to respond to the review comments on time.

Indeed, as you mentioned, the sites appearing in the 2D NMR cannot be determined as resulting from chemical exchange or spin diffusion based solely on the existing data. To enhance the rigour of the article, we have moved the 2D NMR data to the supplementary information as an auxiliary reference, and it will no longer serve as the primary evidence supporting the core arguments of this article. We will use first-principles calculations as the main evidence to support the core arguments of the article. Thank you once again for pointing out this critical scientific issue; we will pay

special attention to the reasonable interpretation of 2D NMR in our future work.

3. The mixing time in 2D EXSY NMR is crucial. we have indicated the MAS rate and delay time in the figure caption to enhance the readability of the data.

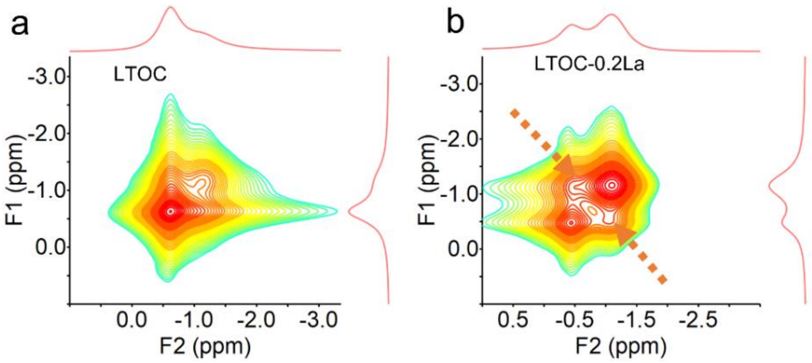

Fig. R69 | 2D EXSY NMR of LTOC and LTOC-0.2La recorded with a mixing time of 0.5 s and a spinning rate of 30 kHz

3#-6、 An additional point with the NMR results in Figure 1: the methods section states that the recycle delays are 0.5 s and 2 s (it's also unclear why there are two used?). At 600 MHz, this can be quite short relative to typical ^{7}Li relaxation times, especially for the LiCl component marked as Li2. Are the authors confident that there is no saturation in these measurements, which is affecting the relative proportions of Li1 and Li2? Also, it doesn't appear that the MAS rate is stated anywhere in the main text or the methods section; this is key for reproducibility

Response: Thank you for your comments. We carefully reviewed the data and identified an error on our part; the relaxation time is 0.5 s, and the 2 s was a typographical error that has been removed.

A delay time of 0.5 s is appropriate for LiCl and will not lead to supersaturation.[3] This selection of time is the result of multiple verifications by the testers in conjunction with the literature to ensure the accuracy and authenticity of the data.

The mixing time in 2D EXSY NMR is crucial. we have indicated the MAS rate and mixing time in the figure caption to enhance the readability of the data (**Fig R69**).

3#7. Line 223: What is ‘super-magnification’ referring to in this context? Phrasing

here is unclear.

Response: Many thanks. We replaced "Such excellent super magnification and ultralong cycle stability are unprecedented in halogen-based ASSBs" with "Such excellent rate capability and ultralong cycle stability are unprecedented in halogen-based ASSBs".

3#-8. Line 293: GITT is used to evaluate the diffusion coefficient and support the notion that there is a lower transmission barrier at the SSE/electrode interface on account of the higher diffusion coefficient. It's not clear what this is higher relative to. A control experiment with un-modulated LTOC would be necessary here to contextualise this statement (the poorer interfacial stability of LTOC at high rate relative to LTLOC would presumably be negligible with perturbative GITT pulses). Also, as this is recording the combined response of the diffusion in both the cathode and the SSE, it's difficult to make any direct inference about the SSE contribution to the performance, especially absent a control.

Response: Many thanks for the suggestions. We have incorporated comparative samples and revised the wording accordingly. Additionally, we have revised the manuscript and supporting information, marking them in yellow.

Assemble ASSBs using the same cathode material while varying the SSEs to investigate the effects of different SSEs on the composite cathode material interface and the overall battery performance. Compared to NCM88/LTOC ASSB, NCM88/LTLOC ASSB has higher ion diffusion.

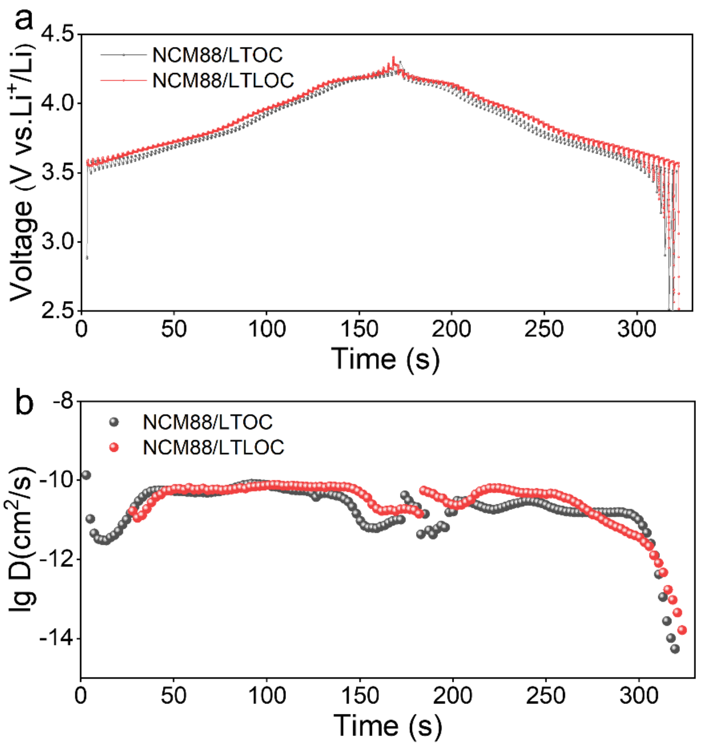

Fig. R70 | a The GITT curves of NCM88/LTOC and NCM88/LTLOC ASSBs. **b** The Li^+ diffusion coefficient of NCM88/LTOC and NCM88/LTLOC ASSBs

3#9. Figure 4: Panels b,c and e,f are essentially presenting the same information, and the figure is a bit cramped overall. Perhaps choosing one style for the DRT as a function of charge/discharge process and having larger panels overall would improve readability?

Response: Thank you for your comments. We have revised the manuscript and supporting information and marked them yellow.

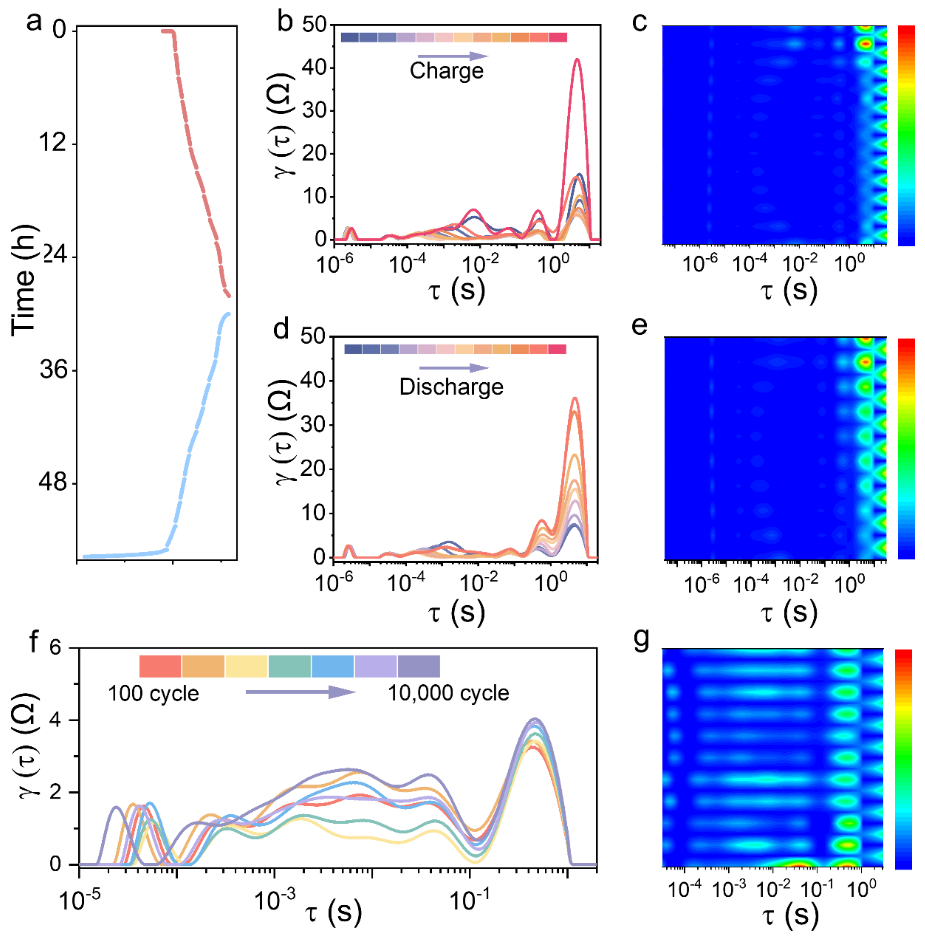

Fig. R71 | Dynamic analysis of NCM88/LTLOC by electrochemical impedance spectroscopy (EIS) and distribution of relaxation time (DRT). **a** The in-situ charge-discharge curve and the discontinuous point align with the EIS testing point. **b** DRT is calculated from the EIS measurements in charge. **d** DRT is calculated from EIS measurements during discharge. **f** DRT calculated from EIS measurements at different cycles. **c**, **e**, and **g** 2D intensity colour map of the charge-dependent DRT curves in (b), (d), and (f), respectively.

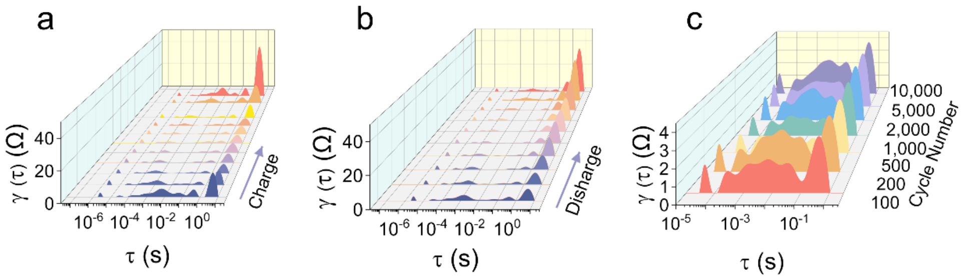

Fig. R72 | a DRT is calculated from the EIS measurements in charge. **b** DRT is calculated from EIS measurements during discharge. **c** DRT calculated from EIS

measurements at different cycles.

3#-10. Line 375: “When $ZrCl_4$ is introduced, only one peak is observed” I infer from later context (and units) that this is the NMR again, but the preceding discussion involved both XPS and TOF-SIMS (which also discuss peaks), so it would be helpful for readability to directly point this out in the text.

Response: Many thanks. We clarified the specific testing content during the discussion process and changed “When $ZrCl_4$ is introduced, only one peak is observed, which corresponds to lithium in an amorphous environment.” to “We utilised SSNMR to analyse the changes in the local environment of lithium following the introduction of various types of fillers. When $ZrCl_4$ is introduced, only one peak is observed in the NMR spectrum, which corresponds to lithium in an amorphous environment.”.We have revised the manuscript and marked it yellow.

3#-11. Line 377: “Lithium in isolation or in a less complex environment exhibits relatively weak signals.” Not sure what this is intended to mean; indeed, in a ‘less complex’ environment (say, a more symmetrical one like LiCl…) ^{7}Li will often have a stronger signal because there is less homogenous broadening and the peak is sharper, all things being equal with populations (indeed, this is part of why the Li2-LiCl-site doesn’t shift much with addition of functional modules, because the local coordination is already highly symmetrical). I think the primary conclusion of these Fig. S21 results is, as the authors point out, that the addition of the functional modules only induces small changes in the material susceptibility (the small shifts) and does not significantly disrupt the amorphous conduction network of the LTOC, so this paragraph should be revised to just reflect this point.

Response: Many thanks for the suggestions. The content has been modified as follows. The highlighted part is the added content.

The introduction of additional substances causes the lithium peak, indicative of the amorphous environment in LTOC, to shift to a higher position (approximately -0.62 ppm), while the position of the other peak remains nearly unchanged. The degree of

peak shift varies with the introduction of different substances, likely due to the differing interactions between these substances and LTOC. Fluoride exhibits the smallest deviation, followed by oxide and chloride. In summary, the NMR data indicate that the introduction of various substances has minimal impact on the local environment of lithium within the LTOC amorphous module, suggesting that the addition of the functional modules only induces small changes in the material susceptibility (the small shifts) and does not significantly disrupt the amorphous conduction network of the LTOC and the basic ion transport characteristics of the amorphous functional module are preserved (Fig. S35).

3#-12. Line 417: While the contrast between LTOC and LTOC-5AlF_3 is clear from the performance of the respective cells after humidity exposure is clear, what is the scientific hypothesis for the role of AlF_3 here? Is it basically just reducing the available surface area for moisture to attack the surface of LTOC particles without completely breaking the conduction network, in contrast to a coating? Mechanistically, this section in particular feels a bit unclear and largely phenomenological.

Response: Thank you for your comments.

1. What is the scientific hypothesis for the role of AlF_3 here?

The scientific hypothesis that AlF_3 can improve humidity stability is based on three main aspects:

(1). From a chemical perspective, fluorides have stronger chemical bonds containing fluorine compared to chlorides, making them less prone to hydrolysis and more stable in the presence of H_2O.

(2). The density of AlF_3 is significantly lower than that of LTOC, and it allows for a high proportion of AlF_3 to combine with LTOC while maintaining a high ionic conductivity.

(3). Based on the first two factors, the low-density AlF_3, which is stable in the presence of H_2O, can effectively reduce the exposed area of unstable components

(bonds containing Cl), thereby achieving a physical protection effect.

2. The reason why AlF_3 can improve the stability of solid-state electrolyte humidity is that it can simultaneously provide chemical and physical protection.

Chemical protection: It has been confirmed that AlF_3, as a functional module, partially interacts with the amorphous phase to form an amorphous structure in conjunction with LTOC. The Ta L_3 edge EXAFS analysis reveals the presence of a Ta-F bond, which is formed through the exchange of fluoride ions (F) from AlF_3 and chloride ions (Cl) from LTOC. This exchange leads to the formation of a stronger Ta-F bond (**Fig. R74**). This interaction results in the formation of stronger Ta-F bonds compared to Ta-O or Ta-Cl bonds, which can mitigate reactivity with water to some extent[8, 9].

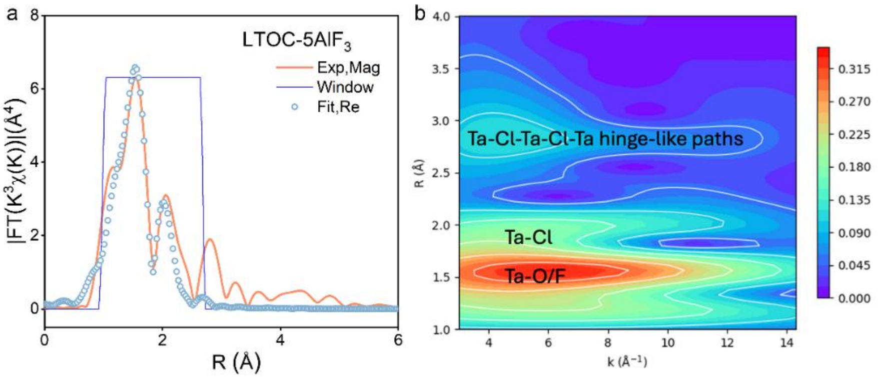

Fig. R73 | a Fourier transform of k^3-weighted Ta L_3-edge EXAFS curves of LTOC-5AlF_3, **b** WT-EXAFS of LTOC-5AlF_3 electrolyte at Ta L_3-edge. A k^3 weighting was used.

Physical protection: According to the chemical reaction equation, it can be inferred that $TaCl_5$ reacts spontaneously and violently with water, whereas AlF_3 does not react with water. Therefore, AlF_3 exhibits a greater tolerance to water compared to $TaCl_5$. Due to the high proportion of AlF_3 introduced, it cannot completely form an amorphous structure and will exist in the form of AlF_3. Coupled with the low density of AlF_3, the portions of AlF_3 exposed to the air surface effectively reduce physical contact between the internal amorphous phase and water in the air and reduce the

available surface area for moisture to attack the surface of LTOC particles without completely breaking the conduction network. Given that AlF_3 itself possesses good air stability, it can enhance the air stability of the electrolyte.

Equations for the Reaction of $TaCl_5$ and AlF_3 with H_2O (Data from MaterialProject):

$$0.25TaCl_5+0.75H_2O \longrightarrow 0.25TaCl_3O+0.5H_3ClO \quad -0.016\ \text{eV/atom}$$

$$AlF_3 + H_2O \longrightarrow AlF_3 + H_2O \quad \text{no reaction}$$

Based on these two reasons, it can be determined that AlF_3 can improve the stability of solid-state electrolyte humidity.

2. Coating is only a physical protection, and it requires extremely high requirements for the thickness and uniformity of the coating layer. However, the improvement of humidity stability by AlF_3 is attributed not only to its physical protection but also to its chemical protection. The synergistic effect of these two protective mechanisms significantly enhances the air stability of the electrolyte. However, it should be noted that this strategy does not fully resolve the issue of air stability in halide electrolytes; rather, it improves their stability to a certain extent.

3#-13. As a general point: I can’t help but think that referring to the functional modules as “filler” does a slight disservice to their role in expanding the capabilities of these materials. Perhaps a different term might be more effective? Or even just sticking with “functional modules” all the way through, rather than flipping back and forth between both terms?

Response: Many thanks for the suggestions. Uniform terminology is crucial in academic writing. Therefore, we have replaced all instances of 'fillers' with 'functional modules' to ensure consistency and rigour throughout the article. We have revised the manuscript and marked it yellow.

References

1. Zhang S, et al. A family of oxychloride amorphous solid electrolytes for long-cycling all-solid-state lithium batteries. *Nat.Commun*. 14, 3780 (2023).

2. Kwak H, et al. Boosting the interfacial superionic conduction of halide solid electrolytes for all-solid-state batteries. *Nat.Commun*. 14, 2459 (2023).

3. Duan H, et al. Amorphous AlOCl Compounds Enabling Nanocrystalline LiCl with Abnormally High Ionic Conductivity. *J. Am. Chem. Soc*. 146, 29335-29343 (2024).

4. Li F, et al. Amorphous Chloride Solid Electrolytes with High Li-Ion Conductivity for Stable Cycling of All-Solid-State High-Nickel Cathodes. *J. Am. Chem. Soc*. 145, 27774-27787 (2023).

5. Yin Y-C, et al. A $LaCl_3$-based lithium superionic conductor compatible with lithium metal. *Nature*. 616, 77-83 (2023).

6. Yu T, et al. Superionic Fluorinated Halide Solid Electrolytes for Highly Stable Li-Metal in All-Solid-State Li Batteries. *Adv. Energy Mater*. 11, 2101915 (2021).

7. Liang J, et al. Site-Occupation-Tuned Superionic Li_xScCl_{3+x} Halide Solid Electrolytes for All-Solid-State Batteries. *J. Am. Chem. Soc*. 142, 7012-7022 (2020).

8. Chen X, Jia Z, Lv H, Wang C, Zhao N, Guo X. Improved stability against moisture and lithium metal by doping F into Li_3InCl_6. *J. Power Sources*. 545, 231939 (2022).

9. Wu M, et al. Fluorinated amorphous halides with improved ionic conduction and stability for all-solid-state sodium-ion batteries. *Nat.Commun*. 16, 2808 (2025).

10. Wu Y, et al. Exploiting the Mixed Entropy Strategy for the Design of Fast Ion Conductors. *Adv. Energy Mater*. 14, 2401528 (2024).

11. Jin H, et al. Regulating Chemical Bonds in Halide Frameworks for Lithium Superionic Conductors. *ACS Nano*. 19, 6399-6411 (2025).

12. Luo Q, et al. O-doping strategy enabling enhanced chemical/electrochemical stability of Li3InCl6 for superior solid-state battery performance. J. Energy Chem. 99, 484-494 (2024).

13. Liu Y, Madanchi A, Anker AS, Simine L, Deringer VL. The amorphous state as a frontier in computational materials design. Nat. Rev. Mater. (2024).

Response to the Comments of Reviewers on " Building Blocks of Battery Innovation: Modular Design of Amorphous Electrolytes"(Research article, #NCOMMS-25-15945B)

We highly appreciate the reviewers' recommendation to publish this paper in **Nature Communications** and are also very grateful to them for providing constructive comments and insightful suggestions for further improving the quality of the manuscript. We have revised the manuscript with great effort. We carefully addressed the concerns and corrected any errors. The specific responses and revisions are listed below. We believe that the paper is now stronger and thank the referees for their constructive comments very much.

The most significant improvements in the revised version of the manuscript can be summarised as follows:

1. The battery performance data have been updated: the electrochemical data under low loading have been removed, and only the electrochemical data under high loading are retained in the article as supporting evidence. Additionally, relevant descriptions and the ordinates of Figure 3f and Figure S18 have been revised.

2. The concept of the functional module has been further elaborated; from the perspective of performance regulation, there is no essential difference between LTOC-0.2La and LTOC-xLa (where x=0.4, 0.6, 0.8, 1.0). Based on their functionality, the added $LaCl_3$ is collectively referred to as the functional module.

3. The explanation of the mechanism for the humidity stability of LTOC-5AlF_3 has been improved.

4. The NMR data have been supplemented and enhanced, with more appropriate references cited for auxiliary verification.

5. Other concerns have also been addressed.

Reviewer 1#

The author tied to answer some of the reviewer' s questions, but some of them still remain unclear.

1#-1. The authors mainly claim their superior electrochemical performance, though the cell performance is not as good as previous reported (Chem. Lett. 2023, 52, 237). claiming best performance is not supported with their data. Furthermore, all the cell loading are still only around 10mg/cm^2, way lower than practical requirement. Within the SI, some of the cells with ~5mg/cm^2 loading, those cell data should be removed. And Figure 2 and S17 a-c are the same set-up, but the low loading cell show worse rate performance than the high loading cell which make no sense and need to explain whether labeled wrong or why.

Response: Thank you for your valuable advice.

The battery performance data have been updated: the electrochemical data under low loading have been removed, and only those under high loading are retained in the article as supporting evidence.

The description of 'best performance' concerning electrochemical performance has been removed.

The subpar rate performance observed in the low-load data may be attributed to the relatively low ionic conductivity of the sulfide electrolyte utilized during that period, which adversely affected the rate performance. Given that the cell loading of this dataset is low, it has been excluded, retaining only the electrochemical data with a higher loading for further support.

1#-2. Figure 3f and S18 with y-axis starting from -50 is wrong.

Response: Thank you for your comments.

The ordinate of Figure 3f has been revised, with its starting coordinate adjusted to zero. Additionally, the data presented in Figure S18, which were collected under low

load conditions, have been removed. We have revised the manuscript and marked it in yellow.

1#-3. With the material LTOC-0.2La, all the data says LaC13 just dope into the materials, as the TEM and XRD show it is complete amorphous and all element evenly distributed, so it is just doping rather so called functional modules

Response: Thank you for your comments.

When the addition of the functional module is minimal, it can indeed be regarded as doping from a structural perspective. However, the functional module we are referring to is akin to a functional additive in electrolytes, where the emphasis is primarily on its functionality. Its structure is complex, potentially existing as either a homogeneous phase (when a small amount of the functional module is present, i.e., doping) or a heterogeneous phase (when an excessive amount of the functional module is present). Although these two types exhibit slight differences in crystal structure, the fundamental distinction lies in the quantity of the functional module added. Therefore, the functional module we discuss underscores that effective regulation of its properties (such as electrochemical performance and humidity stability) can be achieved by introducing additional substances (functional modules). From the perspective of performance regulation, we argue that there is no essential difference between LTOC-0.2La and LTOC-xLa (where x=0.4, 0.6, 0.8, 1.0), and $LaCl_3$ is collectively referred to as the functional module based on its functionality.

1#4. The question about LTOC-5AlF_3 show better moisture stability is somewhat answered partially. Replacing some Cl bonded to Ta with F is clear, with such mechanism $AlF_{3-x}Cl_x$ should be formed too, however will $TaCl_4$-xF_x and $AlF_{3-x}Cl_x$ exhibit better moisture tolerance. Or possible just mainly because much excess AlF_3 on the surface to act as a physical barrier to water.

Response: Many thanks.

We fully concur with your assertion that a significant amount of excess AlF_3 is present on the surface, serveing as a physical barrier to impede moisture ingress.

Concerning the humidity stability mechanism of LTOC-5AlF_3, we have elaborated on this in the supporting information, positing that it primarily hinges on two factors: firstly, a chemical effect, specifically the alteration of chemical bonds following the introduction of the fluorine element; secondly, a physical protective aspect, whereby the moisture-resistant AlF_3 functions as a physical barrier on the material's surface. Moreover, due to the exchange between fluorine and chlorine, the formation of species such as $AlF_{3-x}Cl_x$, $TaCl_{5-x}F_x$, and $AlF_{3-x}Cl_x$ may indeed occur. However, from a chemical bond perspective, the moisture resistance of these species surpasses that of LTOC and its precursor, $TaCl_5$.

Reviewer 2#

The revised manuscript resolves the most of the raised concerns and good to publish in this journal.

Response: Thank you very much for your recognition of the content of this work.

Reviewer 3#

Overall, the authors have done a very reasonable job of addressing the questions posed in the original review. The additional explanations provided are helpful in understanding the role of the functional modules, and the new calculations supporting the MSD increase in the presence of the functional modules greatly buttress the previous results. The flow and readability of the manuscript has also been improved, and as before, the main findings are compelling and likely to be of broad interest in the community.

While I do think that the VT-NMR results would be an asset and increase the rigour of the original EXSY experiments, I also recognize that it is challenging to schedule and conduct such experiments within the short turn-around time of a review response, and the new calculations do work well in demonstrating the key result that was being

inferred from the original, single-temperature EXSY experiments. One point I would still make with the NMR results regards the potential saturation of the ^{7}Li signals (particularly the LiCl component): a citation (#3) is given in the reviewer response to a different manuscript as support for the short recycle delay, but in that study, 5 s was utilized (i.e. an order of magnitude longer, and potentially still too short). Further, it does not follow that a similar error is not being propagated through the literature just because another example exists. The response also states "This selection of time is the result of multiple verifications by the testers in conjunction with the literature to ensure the accuracy and authenticity of the data." The nature of the 'multiple verifications' are not stated (there is really only one certain method: a formal T1 measurement on the sample). However, since the peak area quantifications are relative, not absolute, and (more importantly) primarily support qualitative arguments about the composition, I don't think this can be considered a fatal flaw (especially taking into account that the authors mention that they cannot easily conduct additional NMR experiments with their main facilities).

Correspondingly, I have no further criticisms/comments which have not been adequately addressed in the revisions/response.

Response: Thank you very much for your recognition of the content of this work.

We sincerely apologize for the mislabeling of test parameters, which arose from our conflation of two distinct concepts in NMR experiments: Mixing time and Relaxation delay time. Corresponding corrections to the Mixing time and Relaxation delay time have been implemented. Under the guidance of the reviewer's insightful comments, we have conducted a thorough review of key parameters in NMR measurements (Mixing time, Relaxation delay time, etc.). To ensure data reliability , a formal T1 measurement was performed on the samples; this measurement serves to validate the rationality the test parameters we selected.

We would like to reiterate our sincere gratitude to the reviewer for their valuable feedback. This feedback has not only enhanced the rigor of the data presented in our

manuscript but also deepened our understanding regarding the rational selection of NMR test parameters and the application of NMR techniques in data analysis.

Table R1. Relaxation Times, T1, of LTOC sample derived from ^{7}Li MAS-NMR

Peak name	F2 (ppm)	T1 (s)	error
1	0.472	0.384	0.01415
2	-0.167	1.36	0.3042

Table R2. Relaxation Times, T1, of LTOC-0.2La sample derived from ^{7}Li MAS-NMR

Peak name	F2 (ppm)	T1 (s)	error
1	0.436	0.362	0.01645
2	-0.178	1.35	0.3180
